# Oncogenic Activation of Nrf2, Though as a Master Antioxidant Transcription Factor, Liberated by Specific Knockout of the Full-Length Nrf1α that Acts as a Dominant Tumor Repressor

**DOI:** 10.3390/cancers10120520

**Published:** 2018-12-17

**Authors:** Lu Qiu, Meng Wang, Shaofan Hu, Xufang Ru, Yonggang Ren, Zhengwen Zhang, Siwang Yu, Yiguo Zhang

**Affiliations:** 1 The Laboratory of Cell Biochemistry and Topogenetic Regulation, College of Bioengineering and Faculty of Sciences, Chongqing University, No. 174 Shazheng Street, Shapingba District, Chongqing 400044, China; qiulu99999@163.com (L.Q.); 20151901005@cqu.edu.cn (M.W.); hufan2441@163.com (S.H.); ruxufang@163.com (X.R.); rygqfyy@nsmc.edu.cn (Y.R.); 2 Institute of Neuroscience and Psychology, School of Life Sciences, University of Glasgow, 42 Western Common Road, Glasgow G22 5PQ, Scotland, United Kingdom; zzhengwen@hotmail.co.uk; 3 State Key Laboratory of Natural and Biomimetic Drugs, Department of Molecular and Cellular Pharmacology, Peking University School of Pharmaceutical Sciences, No. 38 Xueyuan Rd., Haidian District, Beijing 100191, China; swang_yu@hsc.pku.edu.cn

**Keywords:** Nrf1α, Nrf2, Keap1, PTEN, COX1, COX2, AP-1, miR-22, proteasome, tumor repressor, tumor promoter, regulatory networks, non-alcoholic steatohepatitis, hepatoma, oxidative stress

## Abstract

Liver-specific knockout of Nrf1 in the mouse leads to spontaneous development of non- alcoholic steatohepatitis with dyslipidemia, and then its deterioration results in hepatoma, but the underlying mechanism remains elusive to date. A similar pathological model is reconstructed here by using human Nrf1α-specific knockout cell lines. Our evidence has demonstrated that a marked increase of the inflammation marker COX2 definitely occurs in *Nrf1α^−/^^−^* cells. Loss of Nrf1α leads to hyperactivation of Nrf2, which results from substantial decreases in Keap1, PTEN and most of 26S proteasomal subunits in *Nrf1α^−/^^−^* cells. Further investigation of xenograft model mice showed that malignant growth of *Nrf1α^−/^^−^*-derived tumors is almost abolished by silencing of Nrf2, while *Nrf1α^+/^^+^*-tumor is markedly repressed by an inactive mutant (i.e., *Nrf2^−/^^−^^ΔTA^*), but largely unaffected by *a priori* constitutive activator (i.e., *caNrf2^ΔN^*). Mechanistic studies, combined with transcriptomic sequencing, unraveled a panoramic view of opposing and unifying inter-regulatory cross-talks between Nrf1α and Nrf2 at different layers of the endogenous regulatory networks from multiple signaling towards differential expression profiling of target genes. Collectively, Nrf1α manifests a dominant tumor-suppressive effect by confining Nrf2 oncogenicity. Though as a tumor promoter, Nrf2 can also, in turn, directly activate the transcriptional expression of *Nrf1* to form a negative feedback loop. In view of such mutual inter-regulation by between Nrf1α and Nrf2, it should thus be taken severe cautions to interpret the experimental results from loss of Nrf1α, Nrf2 or both.

## 1. Introduction

The steady-state lipid levels are crucial for maintaining cellular and organismal homeostasis, not only in term of energy metabolism, but also to prevent potential cytotoxicity. Conversely, excessive nutrients (and metabolic stress) can culminate in a series of severe diseases, such as diabetes, obesity and fatty liver. Notably, non-alcoholic fatty liver disease (NAFLD) affects 25% of the global population, up to 80% of obese people having this disease [1,2]. NAFLD comprises a continuum of pathological conditions varying in severity of liver injury and exacerbation. Among them, non-alcoholic steatohepatitis (NASH) is defined as a serious pathological process along with inflammation and hepatocyte damage, and also hence regarded as a major cause of liver fibrosis, cirrhosis, and even cancer, i.e., hepatocellular carcinoma (HCC), particularly among those caused by unknown etiologies [3,4,5,6]. However, the axiomatic mechanisms underlying development of NASH and malignant transformation into hepatoma remain elusive.

The cumulative evidence obtained from distinct animal models resembling human NASH [6] demonstrates that homeostatic and nutrient-stimulated lipid metabolisms are tightly regulated by multiple layers of diverse signaling to transcription factor networks to monitor precision expression of different target genes [7,8]. Among them, sterol-regulatory element binding protein 1c (SREBP1c) is well established as a key marker and therapeutic target for hepatosteatosis, because transgenic over-expression of this bHLH-ZIP factor leads to hepatosteatosis, but not hepatoma [9]. Also, similar hyperactivation of SREBP1c by knockout of *GP78*, an endoplasmic reticulum (ER) membrane-bound E3 ligase, occurs with age-related obesity, NASH and HCC [10]. Conversely, hepatosteatosis is partially mitigated by deficiency of SREBP1c [11], but sufficiently ameliorated by blockage of SREBP processing by deletion of *SCAP* (SREBP cleavage-activating protein) [12]. These findings indicate an additive involvement of other factors beyond SREBPs in NASH-associated malignant pathology.

Interestingly, spontaneous NASH, concomitantly with massive hepatomegaly and hepatoma, also results from the hepatocyte-specific knockout of *PTEN* (phosphatase and tensin homolog, as a well-known tumor repressor) in mice [13]. Loss of PTEN leads to constitutive activation of the phosphatidylinositol 3-kinases (PI3K)-AKT-mTOR signaling pathway so as to augment expression of metabolic genes regulated by SREBP1c and PPARγ in cancer proliferative cells [14,15,16]. This process is accompanied by nuclear accumulation of Nrf2 (nuclear factor erythroid 2-like 2, thus also abbreviated NFE2L2) in *PTEN*-deficient cells [17,18]. Notably, both Nrf2 and Nrf1 are two principal members of the cap’n’collar (CNC) basic-region leucine zipper (bZIP) family to regulate expression of those antioxidant response element (ARE)-driven genes involved in detoxification, cytoprotection, metabolism and proliferation. Significantly, aberrant accumulation of Nrf2 and activation of target genes are significantly incremented by simultaneous deletion of *PTEN* (leading to a GSK3β-directed phosphodegron of Nrf2 targeting this CNC-bZIP protein to the β-TrCP-based E3 ubiquitin ligase Cullin 1-mediated proteasomal degradation) and *Keap1* (acting as an adaptor targeting Nrf2 to the Cullin 3-mediated proteasomal degradation), resulting in a deterioration of *PTEN^−/^^−^*-leading cancer pathology [19,20,21]. Conversely, malignant transformation of double *PTEN:Keap1* knockout mice is alleviated by additive deletion of Nrf2 [20], implying that Nrf2 promotes carcinogenesis. This is also supported by further observations that increased activity of Nrf2 is required for oncogenic KRAS- driven tumorigenesis [22] and this CNC-bZIP activation by antidiabetic agents accelerates tumor metastasis in xenograft models [23]. Furtherly, non-neoplastic lesions are also caused by constitutive active Nrf2 (caNrf2) mutants lacking the Keap1-binding sites in transgenic mice [24,25], albeit their cytoprotection against carcinogenesis is enhanced. Conversely, investigation of a dominant-negative dnNrf2 mutant (that also suppresses other CNC-bZIP factors, such as Nrf1) has demonstrated that the basal ARE-driven gene expression, but not their inducible expression, is crucial for anti-tumor chemoprevention against the chemical-induced carcinogenesis [26]. Yet, the underlying mechanism by which Nrf2 is determined to exert dual opposing roles in either tumor suppression or promotion remains unknown to date. 

More interestingly, another significant phenotype of spontaneous NASH and hepatoma is also manifested in conditional *Nrf1^−/^^−^* (but not in *Nrf2^−/^^−^* or *Keap1^−/^^−^*) mice, which display a bulk of lipid drops in the ER with dramatic morphological changes [27,28]. After global knockout of *Nrf1^−/^^−^* mice die of severe oxidative stress-induced damages and fetal liver hypoplasia during development [29,30]. By sharp contrast, global *Nrf2^−/^^−^* knockout mice are viable and fertile, without any obvious pathological phenotypes occurring during normal growth and development [31]. Such facts indicate that Nrf1 is not compensated by Nrf2, although both are widely co-expressed in various tissues and also have similar overlapping roles in coordinately regulating ARE-driven cognate genes. Further insights also reveal that Nrf1 exerts unique essential functions, which are distinctive from Nrf2, in maintaining cellular redox, lipid and protein homeostasis, as well as organ integrity, possibly through regulation of distinct subsets of target genes [32,33]. This notion is also reinforced by further investigation of other organ-specific *Nrf1* deficiency or its over-activation in mice, which exhibit distinct pathological phenotypes, such as type 2 diabetes, neurodegenerative and cardiovascular disease [34,35,36,37]. In addition to the functionality of Nrf1 as an indispensable CNC-bZIP transcription factor, it is also identified to act as a directly ER membrane-bound sensor to govern cholesterol homeostasis through the consensus recognition motifs (i.e., CRAC) [38,39] and lipid distribution in distinct tissues [40,41]. However, it is very regrettable that which isoforms of Nrf1 are required to execute its unique physio-pathological functions is unclearly defined, because almost all isoforms of the factor are disrupted to varying extents in the past experimental models described above.

Upon translation of Nrf1, its N-terminal ER-targeting signal anchor enables the nascent full- length protein (called Nrf1α) to be topologically integrated within and around the membranes, while other domains of the CNC-bZIP protein are partitioned on the luminal or cytoplasmic sides [38,42]. Subsequently, some luminal-resident domains of Nrf1α are dynamically repositioned across membranes through a p97-driven retrotranslocation pathway into extra-ER compartments [43,44,45]. In these topovectorial processes of Nrf1α, it is subjected to specific post-translational modifications (e.g., glycosylation, deglycosylation, ubiquitination), and also selective juxtamembrane proteolytic processing of the CNC-bZIP factor so as to yield multiple isoforms with different and even opposing activities, during its maturation into an activator [46,47,48]. In addition, distinct variants of Nrf1, including its long TCF11, short Nrf1β/LCR-F1 and small dominant-negative Nrf1γ/δ, are also generated by alternative translation from various lengths of alternatively-spliced mRNA transcripts [49]. However, each Nrf1 isoform-specific physiological function virtually remains obscure.

Notably, specific gene-editing knockout of Nrf1α leads to a significant increase in the malignant proliferation of *Nrf1α^−/^^−^*-derived hepatoma and the tumor metastasis to the liver in xenograft model mice [50]. This work had revealed that Nrf1α may act as a tumor suppressor, but the underlying mechanism remains unclear. Herein, our present work further reveals that Nrf1 and Nrf2 have mutual opposing and unified inter-regulatory cross-talks towards downstream genes. For instance, aberrant hyperactivation of Nrf2 leads to a constitutive increase of its target cycloxygenase-2 (COX2) in *Nrf1α^−/^^−^* cells. Such hyperactivation of Nrf2 by knockout of Nrf1α is accompanied by substantial decreases in Keap1, PTEN and most of 26S proteasomal subunits. The malignant growth of *Nrf1α^−/^^−^*- derived tumor is significantly prevented by knockdown of Nrf2, while *Nrf1α^+/^^+^*-bearing tumor is also markedly suppressed by knockout of Nrf2, but appears to be unaffected by *a priori* constitutive activator of Nrf2 (i.e., caNrf2^ΔN^). Such distinct phenotypes of these animal xenograft tumors are also determined by differential transcriptomic expression of different subsets of genes regulated by Nrf1α or Nrf2 alone or both. These collective findings have convincingly demonstrated that Nrf1α manifests as a dominant tumor-suppressor to confine Nrf2 oncogenicity. Conversely, although Nrf2 is defined as a tumor promoter, it also directly mediates the transcriptional expression of *Nrf1* so as to form a negative feedback loop.

## 2. Results

### 2.1. The Human Nrf1α^−/^^−^- and Nrf2^−/^^−ΔTA^ -Driven Cell Models are Established

**Figure 1:**
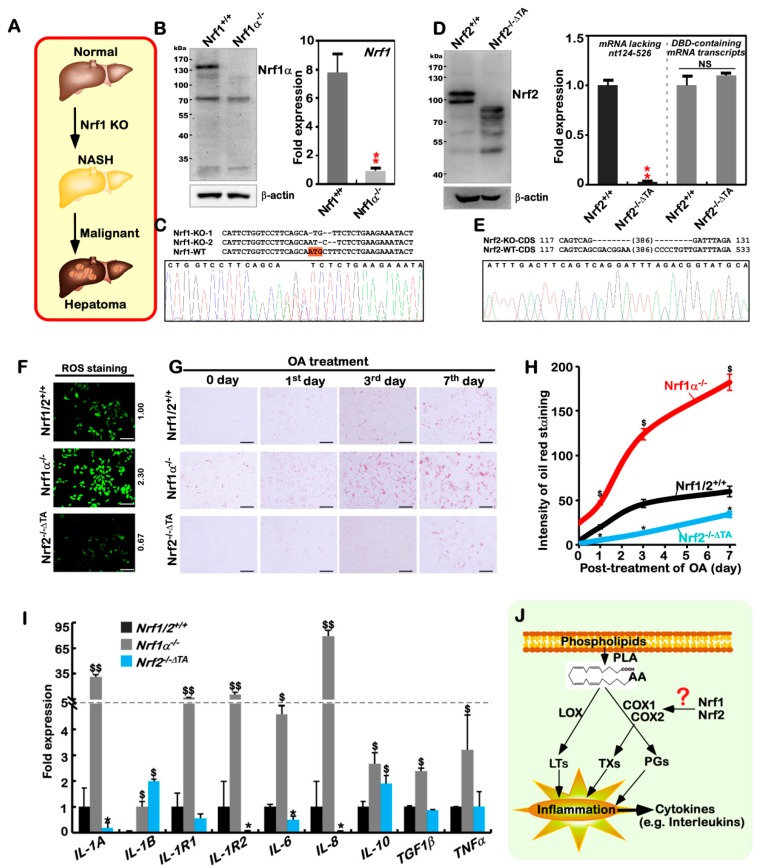
Establishment of Nrf1α-specific knockout cell models with the NASH phenotype. (**A**) Schematic diagrams for the liver-specific *Nrf1^−^^/^^−^* knockout mice that develop spontaneous NASH and deteriorate into hepatoma eventually. (**B**) Both Western blotting (WB, *left*) and real-time quantitative PCR (qPCR, *right*) were employed to identify the protein and mRNA levels of Nrf1 in a monoclonal *Nrf1α^−^^/^^−^* knockout cell line. The data are shown as mean ± SEM (*n* = 3 × 3, * *p* < 0.01). (**C**) Sequencing peaks of the genomic DNA fragments across *Nrf1α*-specific knockout site, as indicated by alignment with wild type (WT) standard sequence. (**D**) Expression of inactive Nrf2 mutant protein and mRNA levels in a monoclonal *Nrf2^−^^/^^−Δ^^TA^* cell line was identified by WB (*left*) and qPCR with distinct primer pairs (*right*). The data are shown as mean ± SEM (*n* = 3 × 3, * *p* < 0.01; NS = no statistical difference). (**E**) Sequencing peaks of the genomic DNA fragments across the *Nrf2*-specific knockout site, as indicated by alignment with normal (WT) sequence. (**F**) ROS staining of *Nrf1/2^+^^/^^+^*, *Nrf1α^−^^/^^−^* and *Nrf2^−^^/^^−Δ^^TA^* cells. They had been treated with 5 μM of 2′,7′-dichlorodihydrofluorescein diacetate (DCFH-DA) for 30 min, before being photographed under a fluorescence microscope. Scale bar = 100 μm. (**G**) Lipid staining of *Nrf1/2^+^^/^^+^*, *Nrf1α^−^^/^^−^* and *Nrf2^−^^/^^−Δ^^TA^* cells, that were or were not treated with 200 μM oleic acid (OA), before being stained with the oil red O agent, and then photographed under a microscope. Scale bar = 25 μm. (**H**) Statistical analysis of the above lipid-stained (**G**) intensity, that was quantified and shown graphically. The data are represented as mean ± SEM (*n* = 3), with significant increases ($) or decreases (*), *p* < 0.01, compared with wild-type values. (**I**) The expression of inflammation-related genes in *Nrf1/2^+^^/^^+^*, *Nrf1α^−^^/^^−^* and *Nrf2^−^^/^^−Δ^^TA^* cells. The data obtained from transcriptome, and FPKM are shown as mean ± SEM (*n* = 3; $, *p* < 0.01; $$, *p* < 0.001 and * *p* < 0.01, by comparison with wild-type). (**J**) Diagrammatic representation of a proposed model for Nrf1 and Nrf2 to regulate COX1 and COX2, essential for arachidonic acid metabolism and relevant inflammatory response.

Since the phenotypes of liver-specific *Nrf1^−/^^−^* mice resemble the human pathogenesis of hepatic steatosis, NASH and HCC [27,28,51], this is thus inferred available for exploring the underlying mechanisms whereby NASH is transformed for malignant progression towards hepatoma (Figure 1A). However, it is unknown whether human Nrf1α exerts similar effects to those obtained from the aforementioned mouse models. For this end, a similar pathological model was here recapitulated by genome-editing knockout of *Nrf1α* from human HepG2 cells, aiming to elucidate the mechanism by which a non-resolving NASH-based inflammation is exacerbated. To achieve the genomic locus- specific knockout of human *Nrf1α*, we herein created a pair of TALEN-directed constructs to yield a specific deletion of Nrf1α-derived isoforms from the single *Nfe2l1* gene, but with shorter variants *Nrf1β* to *Nrf1δ* being unaffected (Figure 1B and see Figure S1A). In the parallel experiments, another pair of CRISPR/Cas9-mediated constructs were also engineered to delete the Nrf2-specific codons (covering its internal 42–175 amino acids within the essential Keap1-binding region and most of its transactivation domains) from the *Nef2l2* gene so as to yield an inactive mutant *Nrf2^−/^^−ΔTA^* (Figure 1D,E and Figure S1B). Consequently, two monoclonal hepatoma cell lines of *Nrf1α^−/^^−^* and *Nrf2^−/^^−ΔTA^* were, respectively, established and also confirmed to be true by sequencing their genomic DNAs, and Western blotting with specific antibodies (Figure 1B–E).

Further real-time qPCRs, with specific primers that recognized distinct nucleotide fragments, showed that knockout of *Nrf1α* substantially abolished expression of total *Nrf1* mRNAs in *Nrf1α^−/^^−^* cells (Figure 1B, *right panel*). Similar results were also obtained from other clones of *Nrf1α^−/^^−^* cell lines [50]. Notably, the *Nrf2^−/^^−ΔTA^* cells gave rise to an inactive mutant lacking nt124-526 of Nrf2, but no alterations in basal expression of its DNA-binding domains (DBD)-containing mRNA transcripts were measured (Figure 1D, *right panel*). Thereby, the resulting inactive mutant *Nrf2^ΔTA^* polypeptides may still, theoretically, be recruited to bind Nrf2-target genes. Such binding activity might allow the *Nrf2^ΔTA^* to circumvent the potential competitive occupancy with other complementary factors, upon comparison with loss of the prototypic Nrf2. This distinction is based on the fact that its nuclear localization signal (NLS) within its DBD was deleted to yield *Nrf2^−/^^−Δ^^DBD^* as previously reported [20]. 

A plausible explanation of NASH pathogenesis is preferred to the classic two-hit hypothesis, in which the first hit is hepatosteatosis (caused by the accumulation of cholesterol and lipids), and the second hit is a non-resolving inflammation (induced by oxidative stress) [6,15]. Such being the case, we examined whether *Nrf1α^−/^^−^* cells act accordingly. As anticipated, it is illustrated by measuring the intracellular hydrogen peroxide (H_2_O_2_) as a representative of reactive oxygen species (ROS), that endogenous oxidative stress was strikingly induced in *Nrf1α^−/^^−^* cells, but, to our surprising, slightly relieved by inactive *Nrf2^−/^^−ΔTA^*, when compared with wild-type *Nrf1/2^+/^^+^* progenitor cells (Figure 1F). Subsequently, a significant accumulation of lipids was seen after staining of *Nrf1α^−/^^−^* cells, by comparison with *Nrf2^−/^^−ΔTA^* and *Nrf1/2^+/^^+^* cells (Figure 1G). 

By increasing the time of oleic acid (OA) treatment to 7 days, the lipid accumulation was significantly incremented in *Nrf1α^−/^^−^* cells to a maximum ~182-fold estimated. While compared with ~60-fold accumulation of lipids in *Nrf1/2^+/^^+^* cells, such lipid overload appeared to be substantially alleviated by the inactive *Nrf2^−/^^−ΔTA^* mutant to ~34-fold (Figure 1G,H). In addition to lipid metabolic disorders resulting from loss of Nrf1’s function to regulate *LPIN1*, *PGC-1β* and other metabolic genes [51,52], NASH has a not-yet-identified characteristic of refractory inflammation. Accordingly, we herein determined transcriptional expression of key genes encoding cytokines and their relevant receptors involved in putative inflammatory responses. As expected, the expression of all nine examined genes, encoding IL-1A, IL-1B, IL-1R1, IL-1R2, IL-6, IL-8, IL-10, TGF1α, and TGF1β, was significantly elevated in *Nrf1α^−/^^−^* cells (Figure 1I). By contrast with *Nrf1/2^+/^^+^* cells, mRNA expression of IL-1A, IL-1R2, IL-6 and IL-8 was markedly down-regulated by the inactive *Nrf2^−/^^−ΔTA^*, while only IL-1B and IL-10 expression was still marginally up-regulated, but no changes in other genes were examined (Figure 1I). Collectively, our findings convincingly demonstrate that the NASH-prone phenotypes are recapitulated by employing human *Nrf1α^−/^^−^*-driven cells, in which Nrf2 may also be critical for this pathogenesis.

### 2.2. The Inflammation Marker COX2 Is Up-Regulated, while COX1 Is Down-Regulated, in Nrf1α^−/^^−^ Cells

Development of inflammation (e.g., NASH) and malignant transformation into carcinogenesis has clear relevance to lipid peroxidation, and particularly degradation metabolites of arachidonic acid (AA), such as prostaglandins (PGs), thromboxanes (TXs) and leukotrienes (LTs) [53,54,55]. Within the AA metabolism network, cyclooxygenase 1 (COX1) and COX2 are the two rate-limiting enzymes that convert AA into PGs, of which COX2 is considered as a key inflammation marker [56] and was also identified as a direct target of Nrf2 [57,58]. Since Nrf2 (and Nrf1) is recruited to directly bind the ARE-containing promoters of *COX2* and *COX1* before transactivating both genes, it is thereby hypothesized that hyper-expression of inflammation-related genes in *Nrf1α^−/^^−^* cells is attributable to overstimulation of PG and TX products from the catalyzation by COX2 and COX1 (Figure 1J). To address this, we herein examined whether (and how) the key rate-limiting enzymes in AA metabolism are influenced by loss of *Nrf1α* or *Nrf2* functions. 

**Figure 2:**
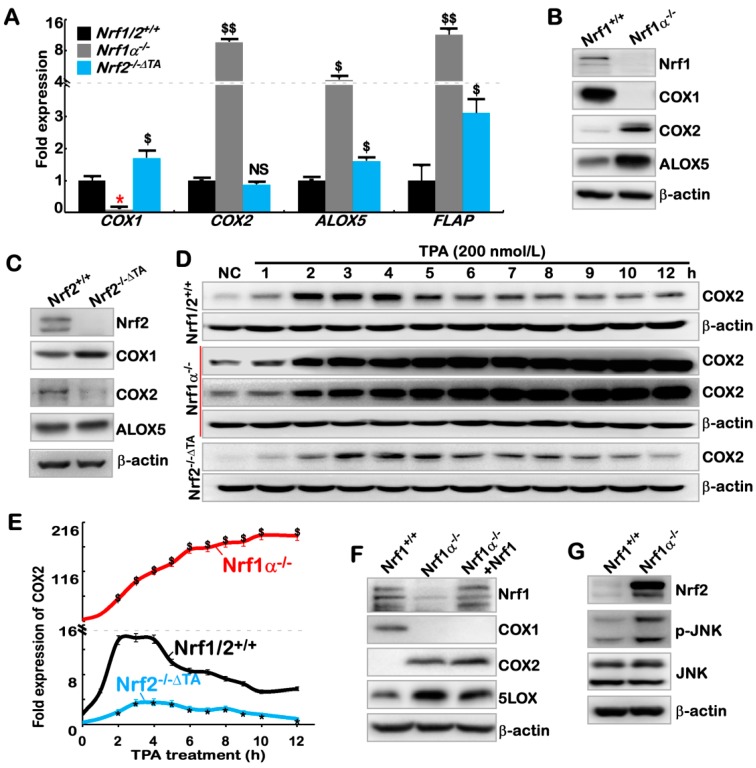

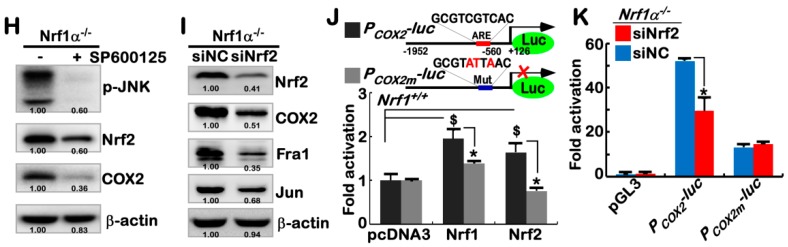
Differential or opposing roles of Nrf1α and Nrf2 in regulating *COX2* and *COX1* genes. (**A**) The mRNA levels of *COX1, COX2*, *ALOX5*, and *FLAP* were determined by real-time qPCR in *Nrf1/2^+^^/^^+^*, *Nrf1α^−^^/^^−^* and *Nrf2^−^^/^^−^^Δ^^TA^* cells. The data are shown as mean ± SEM (*n* = 3 × 3, * *p* < 0.01; $, *p* < 0.01; $$, *p* < 0.001). (**B**) The protein levels of COX1, COX2, ALOX5, Nrf1, and β-actin (as a loading control) in *Nrf1α^−^^/^^−^* and *Nrf1/2^+^^/^^+^* cells were visualized by Western blotting. (**C**) Western blotting of *Nrf1α^−^^/^^−^* and *Nrf2^−^^/^^−^^Δ^^TA^* cells to determine protein levels of COX1, COX2, ALOX5, Nrf1, and β-actin. (**D**) Time-course analysis of COX2 in *Nrf1/2^+^^/^^+^*, *Nrf1α^−^^/^^−^* and *Nrf2^−^^/^^−^^Δ^^TA^* cells, that had been treated for 1 h to 12 h with 100 nM TPA (12-*O*-tetradecanoyl phorbol-13-acetate), before being examined by Western blotting. (**E**) The intensity of the above anti-COX2 immunoblots (*D*) was quantified by normalizing the untreated value, which is shown graphically. The data are shown as mean ± SEM (*n* = 3, * *p* < 0.01; $, *p* < 0.01). (**F**) After the restoration of Nrf1 into *Nrf1α^−^^/^^−^* cells by packaging with the Lentivirus, changed abundances of Nrf1, COX1, COX2, and ALOX5 among *Nrf1/2^+^^/^^+^*, *Nrf1α^−^^/^^−^* and *Nrf1α^−^^/^^−^*+Nrf1-restored cell lines were examined by Western blotting. (**G**) Differences in Nrf2, p-JNK and JNK expression between *Nrf1/2^+^^/^^+^* and *Nrf1α^−^^/^^−^* cells were unraveled by Western blotting. (**H**) The changes in p-JNK, Nrf2 and COX2 were examined, following 24 h treatment of *Nrf1α^−^^/^^−^* cells with 20 μM of SP600125. (**I**) Alterations in Nrf2, COX2, Fra1, and Jun by siRNA interference with Nrf2 in *Nrf1α^−^^/^^−^* cells were determined by Western blotting. (**J**) The human *COX2* promoter-driven reporter *P_COX2_-luc* and its mutant (*upper*) were constructed before the luciferase assay. *Nrf1/2^+^^/^^+^* cells were co-transfected with either *P_COX2_-luc* or mutant, together with an internal control reporter *pRL-TK*, plus an expression construct for Nrf1 or Nrf2, or empty pcDNA3 plasmid, and allowed for 24-h recovery before the *P_COX2_-luc* activity was calculated (*lower*). The data are shown as mean ± SEM (*n* = 3 × 3, * *p* < 0.01; $, *p* < 0.01 compared to the pcDNA3 values). (**K**) Each of *P_COX2_-luc,* its mutant *P_COX2__m_-luc* and the empty vector pGL3 was co-transfected with the control reporter pRL-TK, along with siNrf2 or siNC (as a negative control), into *Nrf1α^−^^/^^−^* cells as described above. The cells were allowed for 24 h recovery from transfection before the luciferase activity was measured. The fold changes in the Nrf2 -mediated activity were calculated as mean ± SEM (*n* = 3 × 3, * *p* < 0.01, compared to controls).

As anticipated, a real-time qPCR analysis revealed that mRNA levels of *COX1* were almost completely abolished in *Nrf1α^−/^^−^* cells, but obviously increased in *Nrf2^−/^^−ΔTA^* cells by comparison to those obtained from wild-type *Nrf1/2^+/^^+^* cells (Figure 2A). Contrarily, the expression of *COX2* was substantially augmented in *Nrf1α^−/^^−^* cells, but almost unaffected by the inactive *Nrf2^−/^^−ΔTA^* mutant when compared to the value measured from *Nrf1/2^+/^^+^* cells. Furthermore, expression of *ALOX5* and *FLAP* in *Nrf1α^−/^^−^* cells was up-regulated at much higher levels than those measured in *Nrf2^−/^^−ΔTA^* cells at considerable levels (Figure 2A).

Next, whether such differences in expression of these AA metabolism genes are attributable to differential and even opposing regulation by Nrf1 and Nrf2 was further examined. Consistently, almost no protein expression of COX1 was detected in *Nrf1α^−/^^−^* cells, while abundances of COX2 and ALOX5 proteins were significantly increased, when compared with *Nrf1/2^+/^^+^* cells (Figure 2B). However, COX1 was highly expressed in *Nrf2^−/^^−ΔTA^* cells at a greater level than that obtained from wild-type cells (Figure 2C). Conversely, COX2 protein expression was substantially diminished or abolished by the inactive *Nrf2^−/^^−ΔTA^* mutant, whereas ALOX5 was almost unaffected (Figure 2C). 

Since it is known that COX1 is constitutively essential for normal physiological homeostasis, while COX2 is an inducibly expressed enzyme to be stimulated by inflammatory stress [59,60], the changing trends of COX2 induction by 12-O-tetradecanoylphorbol-13-acetate (TPA) stimulation are evaluated. As a result, stimulation of *Nrf1/2^+/^^+^* cells by TPA caused an obvious induction of COX2 protein expression to a ~14-fold maximum at 2 h after treatment; this value was being maintained to 4 h, before being gradually decreased to a ~5-fold level by 10-h treatment with TPA (Figure 2D,E). Notably, in *Nrf1α^−/^^−^* cells, the constitute up-expression of COX2 was set to 18-fold as its starting point, the subsequent incremental abundances of this enzymatic protein were further induced to a maximum of ~190-fold by 10-h TPA treatment and maintained until the experiment was terminated (Figure 2E, *red curve*). Relatively, a weak response of COX2 to TPA stimulation of *Nrf2^−/^^−ΔTA^* cells was also observed from 2 h to 8 h, only with a smooth inducible peak at 4 h after treatment (Figure 2E, *blue curve*). Further assays of the luciferase reporter *P_COX2_-Luc* (in which the 2078-bp promoter of human *COX2* gene was constructed) revealed that transcriptional expression of the reporter gene was significantly induced at 4 h after TPA treatment of *Nrf1α^−/^^−^* cells, and such TPA-stimulated increases were continuously maintained until 24 h TPA treatment (*red columns and curve*, Figure S2A,B). However, no obvious changes in the *P_COX2_-Luc* activity were detected in TPA-treated *Nrf2^−/^^−ΔTA^* cells (*blue columns and curve*) when compared with those obtained from *Nrf1/2^+/^^+^* cells (Figure S2A,B). These collective findings imply a striking disparity in the Nrf1α- and Nrf2-mediated induction of COX2 by TPA.

### 2.3. Hyper-Expression of COX2 Results from Increased Nrf2 and JNK-Mediated AP-1 in Nrf1α^−/^^−^ Cells

Intriguingly, the abundance of COX2, as a well-known direct target of Nrf2, was not decreased, but rather marginally increased by ectopic expression of Nrf1 that had been restored into *Nrf1α^−/^^−^* cells (Figure 2F), in which the repressed expression of COX1 was also not rescued, albeit both genes encompass the ARE sites recognized by Nrf1 and Nrf2 [57,58]. These seemingly paradoxical results, along with the above-described data from *Nrf1α^−/^^−^* cells, suggest that Nrf1α may have an ambivalent relationship with Nrf2 in regulating both *COX1* and *COX2* genes. Rather, this confusing but exciting finding arouses our *de facto* curiosity to explore which possible pathways enable Nrf1 to indirectly regulate COX2 (Figure S3A), although this enzyme has been shown to be monitored by CREB, NF-κB, STAT1, FOXM1, ETS1, ELF3 and JNK-regulated AP1 [61,62,63,64,65,66]. Consequently, the real-time qPCR analysis revealed that mRNA levels of only *RELB,* but not other members of the NF-κB family (that regulates cellular responses to inflammation), were significantly up-regulated in *Nrf1α^−/^^−^* cells (Figure S3B). This may be coincident with the notion that ablation of an IκB (inhibitor of NF-κB) kinase IKKγ in liver parenchymal cells causes spontaneous pathology of NASH and HCC [67]. However, Figure S3C showed that abundances of over-expressed COX2 in *Nrf1α^−/^^−^* cells were unaltered by the caffeic acid phenethyl ester (CAPE, as a potent specific inhibitor of NF-κB [68]), and also nor significantly diminished by JSH-23 (as a broad spectrum inhibitor of NF-κB [69]). Thus, it is inferable there implicates an NF-κB-independent pathway to up-regulate expression of COX2 in *Nrf1α^−/^^−^* cells. In addition, it may be not necessary for the modest inducible expression of *ETS1* (one of the E26 transformation-specific transcription factors), because this was accompanied by substantial down-regulation of another family member *ELF3* (Figure S3B).

Further treatments of *Nrf1α^−/^^−^* cells with either of the two CREB inhibitors H-89 and BAPTA- AM [63,70] demonstrated that the elevated expression of COX2 was also unaffected (Figure S3D). However, it is, to our surprise (Figure S3E), found that the forced abundance of COX2 in *Nrf1α^−/^^−^* cells was sufficiently abolished by a JNK-specific inhibitor SP600125 [71]. Further investigations revealed no changes in both total mRNA and protein levels of JNK (Figure 2G and Figure S3B), but the phosphorylated JNK abundance was significantly increased in *Nrf1α^−/^^−^* cells, when compared with those obtained from *Nrf1/2^+/^^+^* cells (Figure 2G). Therefore, it is initially postulated that *Nrf1α^−/^^−^* cells gave rise to the forced expression of COX2 possibly mediated by activation of JNK signaling. Next, in-depth insights into JNK signaling towards downstream target genes unraveled that expression of only *c-Jun*, but not other examined genes, was significantly elevated in *Nrf1α^−/^^−^* cells (Figure S4A,B). Further assays of two luciferase reporter genes *P_COX2_-Luc* and *P_TRE_-Luc* (in which TRE indicates TPA-responsive element inserted within the reporter gene promoter region) verified that AP-1 (a functional heterodimer of Jun and Fos) is also favorably required for the transactivation of COX2 in *Nrf1α^−/^^−^* cells (Figure S4C). By defining distinct AP-1 components (e.g., Jun, Fos, Fra1) at mRNA and protein levels, it is validated that AP-1 was activated in *Nrf1α^−/^^−^* cells, but not in *Nrf2^−/^^−ΔTA^* cells (Figure S4D–F). However, Figure S4G illustrated that hyper-expression of COX2 in *Nrf1α^−/^^−^* cells was not suppressed by the AP-1 inhibitor SR11302 [72]. By stoichiometric analysis of knockdown of Jun or Fra1, only modest decreases of COX2 were no proportional to the silencing of Jun or Fra1 at considerably lower levels (Figure S4H). The latter notion is supported by no dose-dependent effects of silencing Jun on down-regulation of COX2 (Figure S4J, *lower three panels)*. Taken altogether, AP-1 activation by JNK signaling is involved in, but not essential for, making a significant contribution to the reinforced expression of COX2 in *Nrf1α^−/^^−^* cells.

Fortunately, the evidence that expression of Nrf2 and its nuclear translocation are attenuated by the JNK inhibitor SP600125 [73,74] implicates there exists a direct linkage between JNK and Nrf2. Consistently, abundance of Nrf2 protein was surprisingly augmented in *Nrf1α^−/^^−^* cells, which was accompanied by an increase in the phosphorylated JNK (Figure 2G). Similarly, great increases in expression of both COX2 and Nrf2 were caused by knockout of Nrf1α in HL7702^Nrf1^^α^*^−/^^−^* (established on the base of the non-cancerous HL7702 hepatocyte line) (Figure S5A,B). Further examinations of *Nrf1α^−/^^−^* cells unraveled that high-expression of COX2 was substantially suppressed by treatment of the JNK inhibitor SP600125 (Figure 2H) or transfection with Nrf2-targeting siRNA (Figure 2I). The dose-dependent response of silencing Nrf2 to down-regulation of COX2 was determined (Figure S4J, *upper three panels*). Collectively, these findings indicate that the hyper-expression of COX2 in *Nrf1α^−/^^−^* cells is principally caused by increased Nrf2 protein, and the latter CNC-bZIP factor is also monitored by its upstream JNK signaling. This conclusion is supported by *P_COX2_-Luc* reporter assays of *Nrf1α^−/^^−^* cells showing that Nrf2 mediated transactivation of the *COX2* gene driven by its ARE enhancer, but such transactivation was significantly diminished by its ARE mutant (i.e., *P_COX2m_-Luc*) (Figure 2K). Similarly, transactivation of the *P_COX2_-Luc* reporter mediated by ectopic Nrf1, like Nrf2, in wild-type *Nrf1/2^+/^^+^* cells (Figure 2J). This finding, together with above-described data, indicates that Nrf1α also possesses one hand to exert a minor positive effect on COX2 expression by directly binding its ARE enhancer, but also this effect appears to be sufficiently counteracted by the another hand of Nrf1α to elicit a dominant-negative role by indirect inhibitory pathways.

Of note, some of AP-1 abundances (e.g., Jun and Fra-1) (Figure 2I), besides known Nrf2-target genes encoding HO-1 and GCLM (Figure S4I), were obviously suppressed by silencing of Nrf2 in *Nrf1α^−/^^−^* cells (Figure 2I); they were also strikingly prevented by inactive *Nrf2^−/^^−ΔTA^*, by comparison with equivalent controls (Figure S4F). Together with the above-described results, these imply that AP-1 is dominantly repressed by Nrf1α, but positively regulated by Nrf2. However, no available evidence has been presented here to support the notion that AP-1 activates transcription of Nrf2 as reported previously [75]. It is of importance to notice that expression of COX2 in mouse embryonic fibroblasts (MEFs) is co-regulated by both Nrf1 and Nrf2, because its abundance was significantly abolished by global knockout of Nrf1 or Nrf2 (Figure S5C). Here, it should also be noted that global knockout of all the mouse Nrf1 or Nrf2 DNA-binding domain-containing fragments was achieved by their respective gene-targeting manipulations (to yield *Nrf1^−/^^−Δ^^DBD^ or Nrf2^−/^^−Δ^^DBD^*) [28,51]. These resulting mutants are totally distinctive from site-specific knockout by their gene-editing to delete the designed portions of human Nrf1α or Nrf2 (Figure 1 and Figure S1). Moreover, knockout of Keap1 in MEFs (Figure S5D) and human HepG2 (Figure S5E) caused a remarkable increase in the expression of Nrf1, Nrf2, COX2, and HO-1 to different extents as detected. Overall, the precision regulation of COX2 by Nrf1 and/or Nrf2, along with Keap1, in distinct manners, is much preferable to depend on distinctive cell types in different species.

### 2.4. Nrf1α and Nrf2 Transactivate the ARE-Driven miR-22 Signaling to PTEN, but Not to COX1

**Figure 3:**
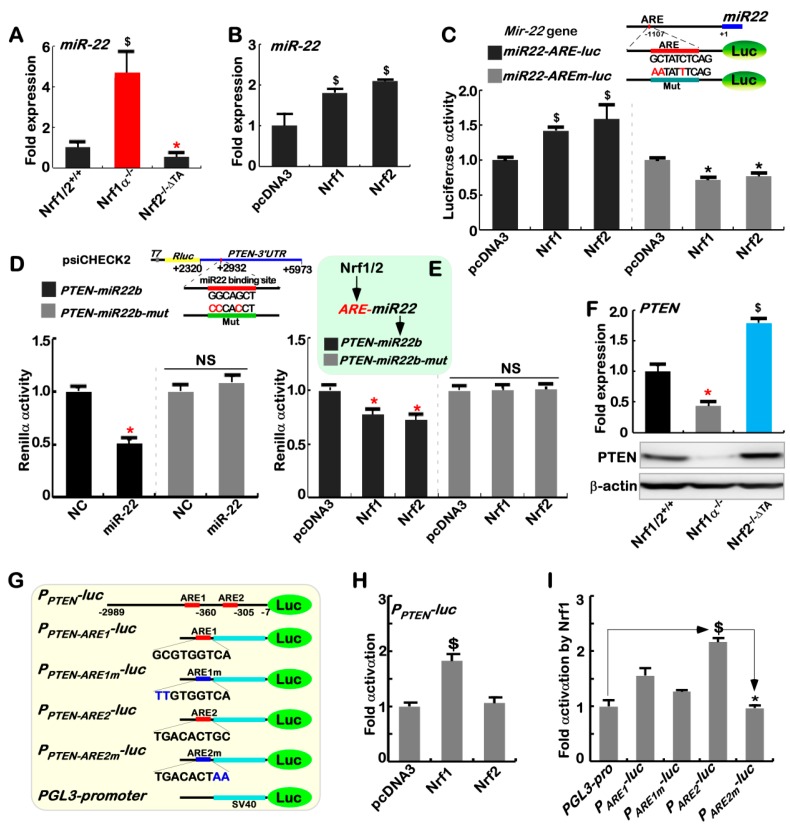
Different regulation of PTEN by Nrf1α and Nrf2 occurs through miR-22. (**A**) The content of miR-22 in *Nrf1/2^+^^/^^+^*, *Nrf1α^−^^/^^−^* and *Nrf2^−^^/^^−^^Δ^^TA^* cells was determined by qPCR with miR-22 specific primers. The data are shown as mean ± SEM (*n* = 3×3; * *p* < 0.01; $, *p* < 0.01 compared to wild-type values). (**B**) The miR-22 expression was altered by transfection of an expression construct for Nrf1 or Nrf2, or an empty pcDNA3 control, into *Nrf1/2^+^^/^^+^* cells. The qPCR data are shown as mean ± SEM (*n* = 3 × 3; $, *p* < 0.01). (**C**) The *miR22-ARE-luc* reporter driven by an ARE enhancer site in the mir-22 gene promoter, and its mutant reporter *miR22-AREm-luc* were constructed. Either of reporter genes as indicated, together with *pRL-TK,* plus each of pcDNA3, Nrf1, or Nrf2 expression constructs, were co-transfected into *Nrf1/2^+^^/^^+^* cells and then allowed for 24-h recovery before the luciferase activity measured. The data are shown as mean ± SEM (*n* = 3 × 3; * *p* < 0.01; $, *p* < 0.01). (**D**) There exists a miR-22 binding site in the PTEN’s 3’UTR region (which was constructed in the dual fluorescent psiCHECK2 vector to yield the *PTEN-miR22b* reporter). Either of *PTEN-miR22b* and *PTEN-miR22b- mut* was co-transfected with miR-22 expression plasmid or a negative control (NC) into *Nrf1/2^+^^/^^+^* cells, and then allowed for 24-h recovery, before the fluorescent activity was determined. The data are shown as mean ± SEM (*n* = 3 × 3; * *p* < 0.01; NS = no statistical difference). (**E**) Either *PTEN-miR22b* or *PTEN-miR22b-mut* was co-transfected with each of pcDNA3, Nrf1, or Nrf2 expression constructs *Nrf1/2^+^^/^^+^* cells and allowed for 24-h recovery, before the fluorescent activity was measured. The data are shown as mean ± SEM (*n* = 3 × 3; * *p* < 0.01). (**F**) Both the mRNA (*upper*) and protein (*lower*) levels of PTEN in *Nrf1/2^+^^/^^+^*, *Nrf1α^−^^/^^−^* and *Nrf2^−^^/^^−^^Δ^^TA^* cells were determined by qPCR and Western blotting, respectively. The data are shown as mean ± SEM (*n* = 3 × 3; * *p* < 0.01; $, *p* < 0.01). (**G**) Schematic representation of the PTEN promoter-containing *P_PTEN_-luc* plasmid, its distinct ARE-driven reporters (*P_ARE1_-luc* and *P_ARE2_-luc*) and indicated mutants, which were constructed into the PGL3-Promoter (i.e., *PGL3-Pro*) vector. (**H**) The *P_PTEN_-luc* and *pRL-TK*, along with an expression construct for Nrf1 or Nrf2, or pcDNA3 were co-transfected into *Nrf1/2^+^^/^^+^* cells and then allowed for 24-h recovery before being measured. The luciferase activity data are shown as mean ± SEM (*n* = 3 × 3; $, *p* < 0.01). (**I**) *Nrf1/2^+^^/^^+^* cells were subject to co-transfection with an indicated luciferase reporter, together with *pRL-TK* and Nrf1 expression construct or pcDNA3 for 24 h before being determined. The data are shown as mean ± SEM (*n* = 3 × 3; $, *p* < 0.01; * *p* < 0.01).

On the contrary to COX2, the isoenzyme COX1 was highly expressed in *Nrf2^−/^^−ΔTA^* cells (Figure 2C), but its expression was almost completely abolished in *Nrf1α^−/^^−^* cells (Figure 2B) and also not rescued by restoration of ectopic Nrf1 into *Nrf1α^−/^^−^* cells (Figure 2F), albeit Nrf2 was up-regulated (Figure 2G). Thus, it is inferable that no matter how Nrf1α and Nrf2 have opposing or overlapping roles in regulating COX1 expression, Nrf2 exerts a dominant inhibitory effect on COX1, but this effect is fully contrary to regulating COX2. In view of this, we speculate that the putative inhibition of COX1 by Nrf2 (and possibly Nrf1α) may be achieved through an indirect miRNA-regulatory pathway, except for directly ARE-binding to this target gene. Fortunately, a candidate miR-22 was selected by predicting its potential miRNA-binding sites within the *COX1* 3′-UTR region (also see http://www.targetscan.org/vert_72/). As expected, relevant real-time qPCR analysis unraveled that miR-22 expression was significantly increased in *Nrf1α^−/^^−^* cells, but this increase was completely attenuated in *Nrf2^−/^^−ΔTA^* cells (Figure 3A). Forced expression of ectopic Nrf1 or Nrf2 also caused an obvious increase in miR-22 expression in wild-type *Nrf1/2^+/^^+^* cells (Figure 3B). Further analysis of the *miR-22*-coding gene revealed there exists a consensus ARE site within its promoter (Figure 3C, *upper panel*). The promoter-driven luciferase reporter (i.e., miR22-ARE-Luc) was created herein, so as to assay for its transcriptional activity. The results showed that the *miR22-ARE-Luc* reporter gene was significantly transactivated by Nrf1 and Nrf2 (Figure 3C), and the transactivation was, rather, diminished by the ARE mutant of *miR22-AREm-luc*. Taken together, these imply direct and indirect transactivation of miR-22 possibly by Nrf1α and Nrf2. 

Since the negative regulation of PTEN by miR-22 had been reported [76,77], a *Renilla* reporter gene containing the 3’UTR region of *PTEN* (i.e. *PTEN-miR22b*) was here constructed, together with a mutant of miR-22-binding site (i.e., *PTEN-miR22b-mut*, Figure 3D, upper panel). If miR-22 would bind the 3’-UTR region of *PTEN* transcripts, the *PTEN-miR22b*-driven *Renilla* reporter activity was significantly reduced by miR22 (Figure 3D, lower panel), and also partially decreased by ectopic expression of either Nrf1 or Nrf2 (Figure 3E). These negative effects were sufficiently abrogated by *PTEN-miR22b-mut*. Consistently, both mRNA and protein levels of PTEN (Figure 3F) were indeed significantly reduced in *Nrf1α^−/^^−^* cells (with hyper-expression of Nrf2, see Figure S6), but strikingly increased by inactive *Nrf2^−/^^−ΔTA^.* Such opposing changes in endogenous PTEN levels are inversely correlated with those relevant values of miR22 measured in the same cell lines (Figure 3A). Thus, transactivation of miR-22 by Nrf1α and Nrf2 leads to putative inhibition of PTEN. 

To further determine whether such miR-22 pathway is involved in the regulation of COX1 by Nrf1 and Nrf2, the luciferase reporter gene was constructed by cloning the 3’-UTR sequence of *COX1* (i.e., *COX1-miR22b*), along with a mutant of the putative miR-22 binding site so as to yield a *COX1-miR22b*-mut reporter (Figure S7A, upper panel). As unexpected, the *COX1-miR22b*-driven *Renilla* activity was roughly unaffected by miR-22, Nrf1 and Nrf2, when compared with that of *COX1-miR22b*-mut (Figure S7A1,A2). The another luciferase reporter gene (i.e., *P_COX1_-Luc*) was engineered by inserting the 1413-bp *COX1* gene promoter, but the *P_COX1_-Luc* activity was also almost unaltered by forced expression of Nrf1 and Nrf2 (Figure S7B1). However, the responsiveness of this ARE-driven *P_COX1_-Luc* reporter to TPA was induced (Figure S7B2), albeit it was relatively weak, when compared to the *P_COX2_-Luc* reporter (Figure S2A). Intriguingly, the *P_COX1_-Luc* activity was also modestly mediated by Jun, but almost unaffected by a canonical AP-1 dimer (Figure S7B3). This is consistent with the notion from a previous report [78], but this is required for further insights into the detailed mechanisms underlying the regulation of COX1 by Nrf1α and Nrf2.

### 2.5. Nrf1α and Nrf2 Have Mutual Inter-Regulatory Effects on Downstream Genes

**Figure 4:**
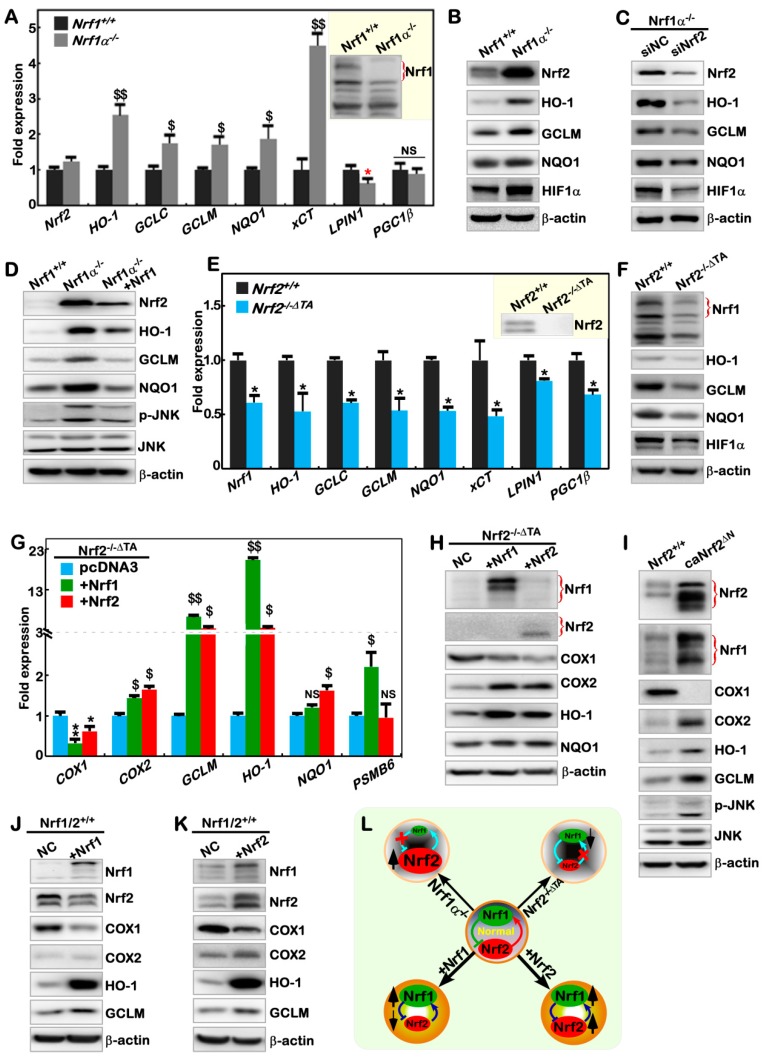
Opposing and unified cross-talks between Nrf1α and Nrf2. (**A**) Real-time qPCR determined the mRNA levels of *Nrf2, HO-1, GCLC, GCLM, NQO1, LPIN1,* and *PGC1β* expressed in *Nrf1/2^+^^/^^+^* and *Nrf1α^−^^/^^−^* cells. The data are shown as mean ± SEM (*n* = 3 × 3, * *p* < 0.01; $, *p* < 0.01; $$, *p* < 0.001. NS = no statistical difference). (**B**) The protein levels of Nrf1, HO-1, GCLM, NQO1 and HIF1α in *Nrf1/2^+^^/^^+^* and *Nrf1α^−^^/^^−^* cells were visualized by Western blotting. (**C**) *Nrf1α^−^^/^^−^* cells were interfered by siNrf2 (at 60 nM) to knock down Nrf2, and then allowed for 24-h recovery for 24 h, before abundances of HO-1, GCLM, NQO1 and HIF1a were examined by Western blotting. (**D**) After Nrf1 was allowed for restoration into *Nrf1α^−^^/^^−^* cells, changed protein levels of *Nrf2, HO-1, GCLM, NQO1, p-JNK and JNK* were determined in *Nrf1/2^+^^/^^+^* , *Nrf1α^−^^/^^−^* cells and *Nrf1α^−^^/^^−^* +Nrf1-restored cells. (**E**) Expression of *Nrf1, HO-1, GCLC, GCLM, NQO1, LPIN1* and *PGC1β* genes in *Nrf1/2^+^^/^^+^* and *Nrf2^−^^/^^−^^Δ^^TA^* cells were analyzed by real-time qPCR. The data are shown as mean ± SEM (*n* = 3 × 3, * *p* < 0.01). (**F**) The protein levels of Nrf1, HO-1, GCLM, NQO1 and HIF1α in *Nrf1/2^+^^/^^+^* and *Nrf2^−^^/^^−^^Δ^^TA^* cells were seen by Western blotting. (**G**) *Nrf2^−^^/^^−^^Δ^^TA^* cells, that had been transfected with an expression construct for Nrf1 or Nrf2 or pcDNA3, were subject to real-time qPCR analysis of *COX1, COX2, GCLM, HO-1, NQO1* and *PSMB6* expression. The data are shown as mean ± SEM (*n* = 3 × 3, * *p* < 0.01, ** *p* < 0.001; $, *p* < 0.01; $$, *p* < 0.001. NS= no statistical difference). (**H**) Western blotting unraveled the changed abundances of Nrf1, Nrf2, COX1, COX2, GCLM, HO-1 and NQO1 proteins in *Nrf2^−^^/^^−^* cells as transfected with an expression construct for Nrf1 or Nrf2. NC = a negative control transfected with empty pcDNA3. (**I**) Alterations in protein levels of Nrf2, Nrf1, COX1, COX2, HO-1, GCLM, p-JNK and JNK in *Nrf1/2^+^^/^^+^* and *caNrf2^ΔN^* (containing the constitutive active Nrf2) cells were determined by Western blotting. (**J**,**K**) *Nrf1/2^+^^/^^+^* cells were transfected with an expression construct for Nrf1 or Nrf2 or pcDNA3 (i.e., NC) and then allowed for a 24-h recovery, before being examined by Western blotting to determine the changes in abundances of Nrf1, Nrf2, COX1, COX2, HO-1 and GCLM. (**L**) A model is proposed to explain there exists an opposing and unifying inter-regulatory cross-talk between Nrf1 and Nrf2.

Since an unusual increase in Nrf2 protein is accompanied by relative higher levels of ROS in *Nrf1α^−/^^−^* cells (Figure 1F, Figure 2G and Figure S6), it is inferable that Nrf1α-deficient hepatoma cells are growing under severe redox stress conditions redefined at a new higher steady-state level that leads Nrf2 to become hyperactive. As anticipated, mRNA expression levels of *HO-1*, *GCLC*, *GCLM*, *NQO1* and *xCT* (though these co-target genes mediated by both Nrf1 and Nrf2 [28,51,79]) were significantly increased in *Nrf1α^−/^^−^* cells (Figure 4A). Meanwhile, a marked decrease in *LPIN1,* but no significant reduction in *PGC-1β* (both were identified as Nrf1-specific target genes by [52]), was determined by comparison of *Nrf1α^−/^^−^* cells with their equivalents of wild-type *Nrf1/2^+/^^+^* cells. Despite no obvious alterations in the mRNA levels of Nrf2 (Figure 4A), Western blotting revealed significant increases in the abundance of Nrf2 protein and typical downstream gene products HO-1, GCLM, NQO1 and HIF1α in *Nrf1α^−/^^−^* cells, by contrast with *Nrf1/2^+/^^+^* cells (Figure 4B). Further determination of Nrf2 subcellular locations showed that its abundance was increased and existed as three polypeptides in the nucleus of *Nrf1α-/-* cells, of which major middle-sized polypeptide was strikingly accumulated in the cytoplasm (Figure S6A,B). All four protein levels of HO-1, GCLM, NQO1 and HIF1α were, however, markedly reduced, in accordance with Nrf2 knockdown by siRNA-targeting interference within *Nrf1α^−/^^−^* cells (Figure 4C). Silencing of Nrf2 also led to decreased mRNA expression levels of *HO-1*, *GCLM* and *xCT* (Figure S5F). Conversely, restoration of ectopic Nrf1 expression into *Nrf1α^−/^^−^* cells caused obvious decreases in abundances of Nrf2, HO-1, GCLM and NQO1 to different extents as detected (Figure 4D). Collectively, it is demonstrated that in *Nrf1α^−/^^−^* cells, hyper-active Nrf2 has a potent ability to mediate a subset of their co-target genes. Furthermore, the phosphorylated JNK, but not its total, protein levels were markedly decreased, as Nrf2 protein was reduced by ectopic expression of Nrf1 after transfecting into *Nrf1α^−/^^−^* cells (Figure 4D). This finding, together with the evidence that Nrf2 is repressed by JNK inhibitor treatment of *Nrf1α^−/^^−^* cells (Figure 2H), implicates that Nrf2 might also govern transcription of a not-yet-identified upstream kinase to phosphorylate JNK through a positive feedback loop. 

By contrast, inactivation of Nrf2 led to strikingly decreases in both mRNA and protein levels of Nrf1 in *Nrf2^−/^^−ΔTA^* cells (Figure 4E,F). This was also accompanied by significant diminishments in the expression of their co-regulated downstream genes *HO-1*, *GCLM*, *NQO1* and *HIF1α* in *Nrf2^−/^^−ΔTA^* cells (Figure 4E,F), in addition to a modest reduction in both mRNA levels of *LPIN1* and *PGC-1β*. Thereby, such marked decreases in the expression of *Nrf1*, *HO-1*, *GCLM*, *NQO1* and *HIF1α* resulting from loss of Nrf2 function demonstrate that *Nrf2^−/^^−ΔTA^* cell line could provide a favorite model to determine the changing downstream genes regulated by Nrf1, Nrf2 alone or both. Next, to address this, *Nrf2^−/^^−ΔTA^* cells were allowed for ectopic expression of Nrf1 or Nrf2 in order to estimate specific downstream genes. As expected, it is validated that Nrf1-specific target gene *PSMB6* was increased by forced expression of Nrf1, but not of Nrf2, allowed for restoration in *Nrf2^−/^^−ΔTA^* cells (Figure 4G). Conversely, expression of *NQO1* was modestly induced by ectopic Nrf2, rather than Nrf1, after being transfected into *Nrf2^−/^^−ΔTA^* cells. This implies that *NQO1* is Nrf2-dependent, but insensitive to Nrf1, in *Nrf2^−/^^−ΔTA^* cells. In fact, Nrf1 and Nrf2 have overlapping roles in mediating transactivation of *HO-1* and *GCLM* (Figure 4G,H). Intriguingly, both CNC-ZIP factors also enhanced expression of *COX2*, but reduced *COX1* expression (Figure 4G,H). This seems fully consistent with additional examinations, revealing that silencing of Nrf2 in *Nrf1α^−/^^−^* cells consequently gave rise to a relative higher expression of *COX1*, as accompanied by a decrease in *COX2* (Figure S5F). Nevertheless, such co-inhibition of COX1 by two transcriptional activators Nrf1 and Nrf2 is much puzzling, albeit it is known that transcriptional expression of downstream genes is mediated by each of their functional heterodimers with a partner of small MAF or other bZIP proteins through directly binding the *cis*- regulatory ARE sites within their target gene promoters [32,80]. Taken together with the above data (Figure 2 and Figure 3), these collective findings indicate that Nrf1 and Nrf2 might also act as two indirect players in the transcriptional regulation of COX1 by an unidentified pathway. 

To determine which specific target genes are constitutively activated by Nrf2, thus a dominant constitutive active mutant caNrf2^ΔN^-expressing cell line was here established by the gene-editing to delete the N-terminal Keap1-binding portion of Nrf2 (Figure S5A). The resulting *caNrf2^ΔN^* cells, indeed, gave rise to a higher expression of Nrf2, as well as Nrf1, when compared to wild-type cells (Figure 4I and Figures S5G and S6C). Interestingly, expression of COX1 almost disappeared as accompanied by significant increases of COX2 in *caNrf2^ΔN^* cells (Figure 4I and Figure S5G). This finding further supports the above-described evidence obtained from inactivation of Nrf1α and Nrf2. Constitutive presence of caNrf2^ΔN^ also led to increases in abundances of both HO-1 and GCLM (Figure 4I), in addition to an enhanced expression of *xCT* and *Lpin1* (Figure S5G). Furthermore, phosphorylated JNK was also significantly induced by caNrf2^ΔN^, with no changes in total JNK protein (Figure 4I), implying there may exist a putative upstream kinase transcriptionally monitored by Nrf2. 

To further assess a mutual regulatory relationship between Nrf1 and Nrf2, we here examined whether one of the endogenous proteins was influenced by the another of both proteins that were allowed for ectopic over-expression in wild-type *Nrf1/2^+/^^+^* cells. As shown in Figure 4J, endogenous Nrf2 protein was obviously decreased by ectopic Nrf1. Consequently, abundances of HO-1 and GCLM were markedly increased, whereas COX2 was weakly enhanced, but COX1 was significantly decreased following over-expression of Nrf1 (Figure 4J). By contrast, over-expression of ectopic Nrf2 caused an enhancement in endogenous Nrf1 (Figure 4K). This was accompanied by striking increases of COX2, HO-1 and GCLM, along with a remarkable decrease of COX1 (Figure 4K). Taken altogether, we assume there exists a mutual inter-regulatory relationship between Nrf1α and Nrf2, as summarized in Figure 4L. This may be an important strategy for a precision regulation of distinct downstream genes, in order to meet the needs for different cell processes.

### 2.6. Nrf1α and Nrf2 Transactivate the Nrf1/Nfe2l1 Gene Promoter-Driven Reporter at Different Sites

**Figure 5:**
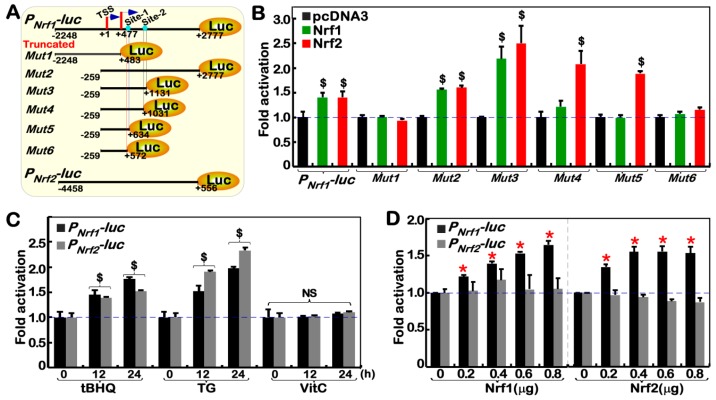

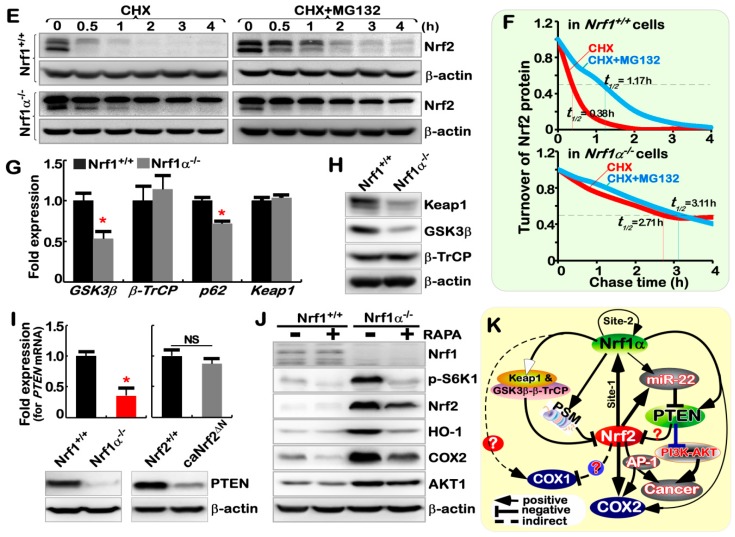
Both Nrf1α and Nrf2 have inter-regulatory cross-talks at distinct levels. (**A**) Schematic representation of *P_Nrf1_-luc* and *P_Nrf2_-luc* reporters (driven by the human *Nrf1* and *Nrf2* gene promoters), along with various lengths of truncated *P_Nrf1_-luc* mutants as indicated, which were constructed in the PGL3-Basic vector. There exist two transcriptional starting sites (i.e., TSS1, TSS2) within the *Nrf1* gene promoter, which contains two *Nrf1/Nef2l1*-regulatory locus sites (i.e. Site-1, Site-2, and also see Figure S8A). (**B**) Each of the *P_Nrf1_-luc* and indicated mutants, together with *pRL-TK*, plus an expression construct for Nrf1 or Nrf2, or empty pcDNA3, were co-transfected into *Nrf1/2^+^^/^^+^* cells and allowed for 24-h recovery, before the luciferase activity was measured. The data are shown as mean ± SEM (*n* = 3 × 3; $, *p* < 0.01 compared with the co-transfection with *P_Nrf1_-luc* and pcDNA3). (**C**) *Nrf1/2^+^^/^^+^* cells were co-transfected with either *P_Nrf1_-luc* or *P_Nrf2_-luc,* together with *pRL-TK*, and allowed for 24-h recovery, before being treated with 50 μM tBHQ (*tert*-butylhydroquinone), 1 μM TG (thapsigargin) or 200 μM VC (vitamin C) for additional 24 h, respectively. The data are shown as mean ± SEM (*n* = 3 × 3; $, *p* < 0.01. NS = no statistical difference). (**D**) Either *P_Nrf1_-luc* or *P_Nrf2_-luc,* plus *pRL-TK*, and an expression construct for Nrf1 or Nrf2 at the concentrations as indicated, were co-transfected into *Nrf1/2^+^^/^^+^* cells and then allowed for 24-h recovery before being determined. The data are shown as mean ± SEM (*n* = 3 × 3; * *p* < 0.01). (**E**) The pulse-chase analysis of Nrf2 in *Nrf1/2^+^^/^^+^* and *Nrf1α^−^^/^^−^* cells were carried out after treatment of the cells with 50 μg/mL of cycloheximide (CHX) alone or plus 5 μM of proteasomal inhibitor MG132 for various lengths of time as indicated. (**F**) The stability of Nrf2 was determined with its half-life in *Nrf1/2^+^^/^^+^* and *Nrf1α^−^^/^^−^* cells as treated above (**E**). (**G**) Expression of *GSK3β*, *β-TrCP*, *p62* and *Keap1* at their mRNA levels in *Nrf1/2^+^^/^^+^* and *Nrf1α^−^^/^^−^* cells were examined. The qPCR data are shown as mean ± SEM (*n* = 3 × 3, * *p* < 0.01). (**H**) The protein abundances of Keap1, GSK3β and β-TrCP in *Nrf1/2^+^^/^^+^* and *Nrf1α^−^^/^^−^* cell lines were determined by Western blotting. (**I**) The mRNA (*upper column*) and protein (*lower panel*) levels of PTEN in *Nrf1/2^+^^/^^+^*, *Nrf1α^−^^/^^−^* and *caNrf2^ΔN^* cells were determined. The data are shown as mean ± SEM (*n* = 3 × 3, * *p* < 0.01, NS = no statistical difference). (**J**) *Nrf1/2^+^^/^^+^* and *Nrf1α^−^^/^^−^* cells had been treated with 100 nM rapamycin (RAPA) for 24 h, before being visualized by Western blotting to detect the changes of p-S6K1, AKT1, Nrf1, Nrf2, HO-1, and COX2 proteins. (**K**) An inter-regulatory model is proposed to explain mutual opposing and unifying cross-talks between Nrf1 and Nrf2 at distinct levels.

To gain insights into the direct relationship between Nrf1 and Nrf2, we here constructed their specific luciferase reporters by cloning the promoter regions of *Nrf1* and *Nrf2* genes and evaluated their activity by transfection into HepG2 cells (Figure 5A,B). As anticipated, the results showed that both *P_Nrf1_-luc* and *P_Nrf2_-luc* reporter genes were significantly induced by thapsigargin (TG, a classic ER stressor), or *tert*-Butylhydroquinone (tBHQ, a typical oxidative inducer), but not vitamin C (VC, a dual redox inducer) (Figure 5C). Thereby, either *P_Nrf1_-luc* or *P_Nrf2_-luc* reporters is available to assess transcriptional expression of *Nrf1* and *Nrf2*, respectively. Subsequent co-transfection of expression constructs for Nrf1 or Nrf2, together with *P_Nrf1_-luc* or *P_Nrf2_-luc* reporters, revealed that transcription of *P_Nrf1_-luc*, but not *P_Nrf2_-luc*, reporter genes was markedly induced by Nrf1 and Nrf2 (Figure 5D). 

Although none of canonical ARE sequences (5′-TGACxxxGC-3′) exist within the 5025-bp *Nrf1* gene promoter, an attempt to identify which sites are located in the promoter enabling for specific transactivation mediated by Nrf1 and Nrf2 was made here, in order to yield a series of truncated mutants from the *P_Nrf1_-luc* (Figure 5A). Fortunately, the resulting luciferase assays uncovered that several reporters containing the first exon region of *Nrf1* were activated by Nrf1 and Nrf2 possibly through different regulatory sites (Figure 5B). From various lengths of the *P_Nrf1_-luc* and mutants, it is inferable that the *Nrf1*/*Nfel1*-regulatory locus site-1 (i.e., Site-1) specific for Nrf2 is located in a 62-bp range between +572 bp and +634 bp, and the *Nrf1*/*Nfel1*-regulatory locus site-2 (i.e., Site-2) specific for Nrf1 *per se* is situated in another 100-bp range from +1031 bp to +1131 bp (Figure S8A).

### 2.7. Nrf1α^−/^^−^-Leading Accumulation of Nrf2 Results from Decreased Keap1

The putative inter-regulation between Nrf1α and Nrf2 was further investigated to interpret the rationale underlying an abnormal accumulation of Nrf2 protein with no changes in its mRNA expression in *Nrf1α^−/^^−^* cells (Figure 2G and Figure 4A). Based on this finding, combined with the notion that Nrf1, but not Nrf2, acts as a primary transcriptional regulator of 26S proteasomal subunits [81,82], thereby it is hypothesized that aberrant accumulation of Nrf2 results from loss of Nrf1α’s function leading to an imbalance between Nrf2 protein synthesis and degradation processing. As shown in Figure S8B,C, total protein ubiquitination was significantly accumulated in *Nrf1α^−/^^−^* cells, but not in *Nrf2^−/^^−ΔTA^* cells, when compared with wild-type cells. Further analysis of mRNA expression levels revealed that 21 of 36 genes encoding all 26S proteasomal subunits and relevant regulatory proteins were significantly reduced by knockout of Nrf1α (Figure S8D1–D4), with only an exception of *PSMB10* enhanced (Figure S8D3). By contrast, no marked changes in transcriptional expression of 24 of the aforementioned 36 genes were determined in *Nrf2^−/^^−ΔTA^* cells (Figure S8D1–D4). Of note, only 8 genes including *PSMB3, PSMB5, PSMB6, PSMB7, PSMB10, PSMC5, PSMD3* and *PSMD11* were marginally down-regulated by *Nrf2^−/^^−ΔTA^* (with decreased Nrf1 expression), but the remaining 4 genes *PSMC2, PSMC6, PSMD1* and *PSME1* were significantly up-regulated (Figure S8D2–D4). Overall, such proteasomal dysfunction by loss of Nrf1α may result in an accumulation of Nrf2 by impaired 26S proteasomal degradation pathway, while it is important that some of the proteasomal regulatory subunits might be, rather, suppressed by Nrf2 to form a positive feedback loop. 

To address this, turnover of Nrf2 protein was further determined by pulse-chase analysis of its half-life in *Nrf1α^−/^^−^* cells (Figure 5E). Surprisingly, it was herein found that *Nrf1α^−/^^−^* cells gave rise to relatively stable protein of Nrf2 with a prolonged half-life to 2.71 h (= 163 min) after treatment with cycloheximide (CHX, an inhibitor of newly-synthesized polypeptides), but such a longevity of Nrf2 was largely unaffected by the proteasome inhibitor MG132 (Figure 5F, *lower panel*). As controls, *Nrf1^+/^^+^* cells displayed a rapid turnover of Nrf2 with a short half-life of 0.38 h (= 23 min) after CHX treatment, and this lifetime was also significantly extended to 1.17 h (= 70 min) by MG132 (Figure 5F, *upper panel*). These collective findings convincingly demonstrate that aberrant accumulation of Nrf2 in *Nrf1α^−/^^−^* cells results from impaired 26S proteasome-mediated degradation of this protein. 

Next, several upstream regulators of Nrf2 were examined, so as to determine which pathways are impaired towards its protein turnover in *Nrf1α^−/^^−^* cells. Intriguingly, abundance of Keap1 protein was significantly decreased (Figure 5H), even though its mRNA expression levels were unaltered, along with its turnover regulator *p62* was strikingly reduced in *Nrf1α^−/^^−^* cells (Figure 5G). Thereby, the turnover of Keap1 in *Nrf1α^−/^^−^* cells may also occur through a p62-independent pathway. As such, impairment of Keap1-mediated proteasomal degradation of Nrf2, in particular oxidative stress [83], is likely to contribute to an accumulation of Nrf2 by loss of Nrf1α. However, aberrant accumulation of Nrf2 is also attributable to impairment of GSK3β-phosphorylated β-TrCP-mediated proteasomal degradation of the CNC-bZIP protein. This is due to a marked decrease of GSK3β at its mRNA and protein levels in *Nrf1α^−/^^−^* cells (Figure 5G,H). The resulting consequence is that Nrf2 is markedly accumulated in both the cytoplasm and nucleus of *Nrf1α^−/^^−^* cells (Figure S6A,B).

### 2.8. Nrf1α and Nrf2 Exert Opposing and Unifying Roles in the Regulation of PTEN Signaling

More importantly, we found that both protein and mRNA levels of PTEN, which acts as a key master versatile regulator of Nrf2, Keap1, PI3K, AKT and GSK3β [17,19,20,84], were significantly diminished or even abolished in *Nrf1α^−/^^−^* cells (retaining high expression of Nrf2) (Figure 5I, *left panel*). In contrast, inactivation of Nrf2 caused a striking increase in PTEN mRNA, but not its protein, levels in *Nrf2^−/^^−ΔTA^* cells (albeit with a low expression level of Nrf1) (Figure 3F and Figure 4F). On the contrary, *caNrf2^ΔN^* cells (yielding high expression of Nrf2 and Nrf1, Figure 4I) caused a significant decrease in expression of PTEN protein, but not its mRNA levels (Figure 5I). Collectively, together with the data (as shown in Figure 3), both Nrf1α and Nrf2 are much likely to exert opposing and unifying roles in the precision regulation of PTEN by both miR-22-dependent and -independent pathways, in which Nrf2 is preferably dominant-negative, whereas Nrf1α has a limited positive role.

Further analysis of the *PTEN* gene unraveled that there exist two typical ARE sites within its promoter region (Figure 3G). The resulting luciferase assay demonstrated that transcription activity of *PTEN* promoter-driven luciferase reporter *P_PTEN_-luc* was significantly induced by Nrf1, but not by Nrf2 (Figure 3H). Mutagenesis analysis uncovered that the second ARE2 site made a primary contribution to transactivation activity of the *P_PTEN_-luc* reporter mediated by Nrf1, whilst the first ARE1 site also gained a minor contribution to Nrf1-mediated transactivation of *P_PTEN_-luc* (Figure 3I). 

Based on the fact that loss of PTEN function leads to constitutive activation of the PI3K-AKT signaling pathway to augment the nuclear accumulation of Nrf2 and its resulting activation [17,18], we determined whether the PI3K-AKT signaling is activated by abolishment of PTEN in *Nrf1α^−/^^−^* cells (where Nrf2 is aberrantly accumulated). The results demonstrated *Nrf1α^−/^^−^*-leading increased abundances of Nrf2, AKT1, COX2 and HO-1, but their increases were also significantly suppressed by rapamycin (RAPA, a classic mTOR inhibitor) (Figure 5J). This implies that mTOR signaling may be constitutively activated in *Nrf1α^−/^^−^* cells. Accordingly, the increased abundances of both AKT and phospho-S6K1 in *Nrf1α^−/^^−^* cells were markedly blocked by the mTOR inhibitor RAPA. This appears inversely correlative with the consequence that over-expression of Nrf1 suppresses AKT induction [36]. Taken together, this further indicates mutual opposing and unifying cross-talks between Nrf1α and Nrf2 to regulate the PTEN-mTOR-AKT signaling towards the Nrf2-COX2 pathway. 

From these findings, we here summarized an endogenous inter-regulatory network of between Nrf1α and Nrf2 (Figure 5K). Consistently, the conclusion is further validated by a series of similar experimental evidence obtained from additional seven distinct monoclonal cell lines of *Nrf1α^−/^^−^*; they had been established by gene-editing knockout of this gene from two progenitor cell lines of HepG2 and HL7702 (Figure S9).

### 2.9. Blockage of Nrf1α^+/^^+^-Bearing or Nrf1α^−/^^−^-Derived Tumor Growth by Nrf2 Deficiency

**Figure 6:**
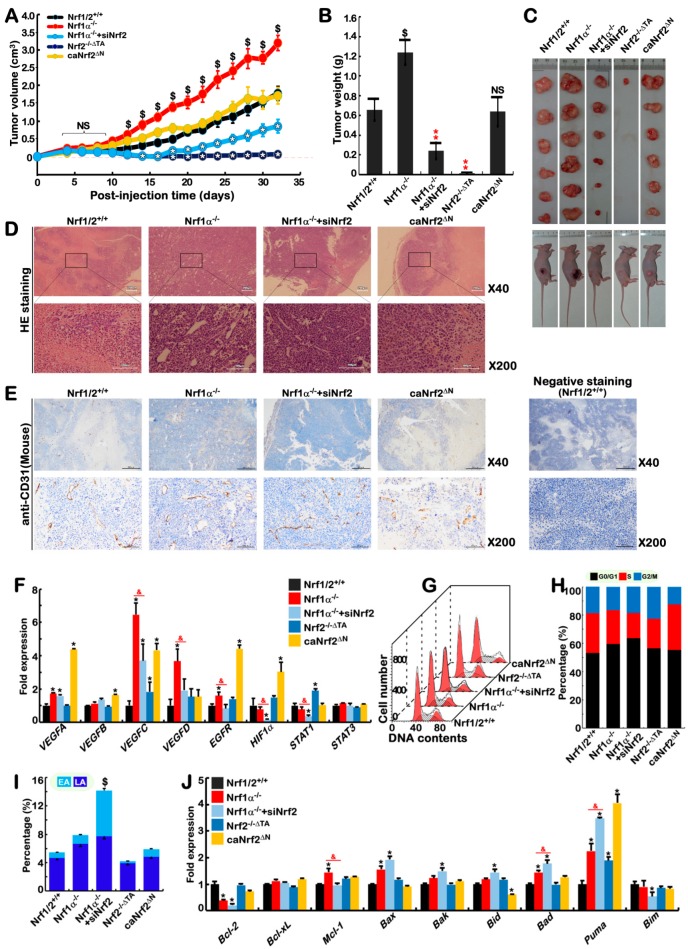
Distinctive animal tumor phenotypes of *Nrf1α^−/^^−^*, *Nrf1α^−/^^−^*+siNrf2, *Nrf2^−/^^−^^Δ^^TA^* and *caNrf2^ΔN^* derived from *Nrf1/2^+/^^+^* cells. (**A**) Differences in mouse subcutaneous xenograft tumors derived from *Nrf1/2^+/^^+^*, *Nrf1α^−/^^−^ Nrf1α^−/^^−^*+siNrf2, *Nrf2^−/^^−^^Δ^^TA^* and *caNrf2^ΔN^* cells were measured in size every two days, before being sacrificed on the 32^nd^ day. The data are shown as mean ± SEM (*n* = 6 per group, * *p* < 0.01; $, *p* < 0.01, NS = no statistical difference at the early incubation phase). (**B**) The final tumor weights of all groups were calculated and the data are shown as mean ± SEM (*n* = 6, ** *p* < 0.001; $, *p* < 0.01, NS = no statistical difference, when compared to the wild-type). (**C**) Distinctive animal xenograft tumors derived from *Nrf1/2^+/^^+^*, *Nrf1α^−/^^−^ Nrf1α^−/^^−^*+siNrf2, *Nrf2^−/^^−^^Δ^^TA^* and *caNrf2^ΔN^* cells. (**D**) The histological photographs of indicated tumors were achieved by HE (hematoxylin & eosin) staining. The scale bar = 200 μm in ×40 pictures, or = 100 μm in ×200 pictures. (**E**) Evaluation of tumor angiogenesis by immunohistochemical staining with a specific marker CD31 antibody. The negative staining was set up by the non-immune serum to replace the primary antibody. Scale bar = 500 μm (×40) or = 100 μm (×200). (**F**) The qPCR analysis of some angiogenesis-related genes in distinct cells as indicated was validated by transcriptome. The data are shown as mean ± SEM (*n* = 3 × 3, * *p* < 0.01, ** *p* < 0.001; $, *p* < 0.01; $$, *p* < 0.001). (**G**,**H**) The flow cytometry analysis of distinct cell cycle was indicated. The data (*n* = 3) are shown in two different fashions. (**I**) The early apoptosis (EA) and late apoptosis (LA) of five distinct cell lines were examined by flow cytometry. The data are shown as mean ± SEM (*n* = 9; $, *p* < 0.01). (**J**) Expression of some apoptosis-related genes in indicated cells was transcriptomically analyzed. The data are shown as mean ± SEM (*n* = 3, * *p* < 0.01, ** *p*< 0.001; $, *p* < 0.01; $$, *p* < 0.001).

Our previous work revealed that the *in vivo* malignant growth of *Nrf1α^−/^^−^*-derived hepatoma is accompanied by metastasis to the liver in xenograft mice [50]. Herein, to elucidate what effects have been elicited by Nrf1α and Nrf2 on tumor repression or promotion, we further investigate distinct genotypic tumors derived from *Nrf1/2^+/^^+^*, *Nrf1α^−/^^−^*, *Nrf1α^−/^^−^+siNrf2*, *Nrf2^−/^^−ΔTA^* and *caNrf2^ΔN^* cells in xenograft mice. Their tumorigenicity was evaluated by measuring tumor volumes and weights. As illustrated in Figure 6A–C, *Nrf2^−/^^−ΔTA^* cells were inoculated in nude mice, but did not form more than one solid tumor. This strongly implies that the tumorigenicity of *Nrf1/2^+/^^+^* cells, as controls, is almost completely abolished by inactivation of Nrf2. Conversely, constitutive activation of Nrf2 did not obviously influence the resulting *caNrf2^ΔN^*-driven tumorigenicity, by comparison to that of *Nrf1/2^+/^^+^*. This indicates that Nrf2-prone cancer promotion is dominantly confined by the presence of Nrf1α. Just such loss of Nrf1*α* function in *Nrf1α^−/^^−^* cells (albeit hyper-active Nrf2 is accumulated), enabled the resultant tumorigenicity to become significantly higher than that of *Nrf1/2^+/^^+^*-tumor, but rather was much strikingly suppressed by silencing of Nrf2 (in the *Nrf1α^−/^^−^+siNrf2*-derived tumors) to the much less extent than that of *Nrf1/2^+/^^+^* control cells (Figure 6A–C). Collectively, these findings demonstrate that Nrf2 acts as a tumor promoter, but it is efficiently confined by Nrf1α serving as a dominant tumor repressor, implying both are a pair of mutual antagonizing twin factors. Overall, malignant transformation of *Nrf1α^−/^^−^*-derived cells is attributable to hyper-activation of Nrf2. 

Histological examination showed that a considerable number of blood vessels were markedly formed in *Nrf1α^−/^^−^* tumors, but were reduced by Nrf2 knockdown in *Nrf1α^−/^^−^+siNrf2*-derived tumors (Figure 6D,E). However, no marked differences in the vascularity of between *caNrf2^ΔN^*- and *Nrf1/2^+/^^+^*-bearing tumors were observed. Further insights into angiogenesis-related genes revealed that mRNA expression levels of *VEGFA*, *VEGFC*, *VEGFD*, *EGFR,* but not of *HIF1α* or *STAT1* were strikingly elevated by knockout of Nrf1α, but the increased expression of *VEGFC*, *VEGFD and EGFR* was significantly reduced by silencing of Nrf2 (Figure 6F). Notably, knockdown of Nrf2 almost completely abolished expression of *HIF1α* and *STAT1* in *Nrf1α^−/^^−^+siNrf2* cells, but no changes in these two factors were observed in *Nrf1α^−/^^−^* cells, as compared to those obtained from *Nrf1/2^+/^^+^* cells. Rather, all other angiogenesis genes except *VEGFD* were up-regulated in *caNrf2^ΔN^* cells (giving high expression of Nrf1 and Nrf2), while only *STAT1* but not other genes were up-regulated by inactive *Nrf2^−/^^−ΔTA^* mutant (Figure 6F). Altogether, both Nrf1α and Nrf2 are diversely involved in regulating the expression of angiogenesis genes except for *STAT3* as examined above. 

Intriguingly, the vascularity of *Nrf1α^−/^^−^+siNrf2*-derived tumors seemed to be higher than that of *Nrf1/2^+/^^+^*-bearing tumors (Figure 6D), but such angiogenetic changes cannot serve to explain the observation that the *Nrf1α^−/^^−^+siNrf2*-tumor volumes and weights were significantly less than those obtained from the *Nrf1/2^+/^^+^*-tumors. This implicates other rationales beyond angiogenesis. Thus, we employed flow cytometry to determine changes in the cell cycle and apoptosis in five distinct cell lines. As shown in Figure 6G,H, the S-phase of *Nrf1α^−/^^−^+siNrf2* cells was significantly shortened. Such a severe S-phase arrest of cell cycle is also further supported by quantitative analysis of gene expression, revealing that significant up-regulation of *p16*, *p19*, *p21 p53* and *CDK4* was accompanied simultaneously by down-regulation of *RB1, CDK1, CyclinA2, CyclinB2, E2F3, E2F5,* and *E2F6* in *Nrf1α^−/^^−^+siNrf2* cells, when compared with its progenitor *Nrf1/2^+/^^+^* or *Nrf1α^−/^^−^* cells (Figure S10A). 

In addition to the S-phase arrest, the G0/G1-phase was relatively extended in *Nrf1α^−/^^−^+siNrf2* cell cycle (Figure 6H). Consistently, apoptosis of *Nrf1α^−/^^−^+siNrf2* cells was significantly enhanced, when compared with other cell lines (Figure 6I and Figure S10B–F). This is also supported by further analysis of apoptosis-related genes, unraveling that *Bax, Bak, Bid, Bad*, and *Puma* were significantly up-regulated, while anti-apoptotic *BCL-2* gene was down-regulated, with no changes in *BCL-xL* and *Mcl-1* in *Nrf1α^−/^^−^+siNrf2* cells (Figure 6J).

Although no significant differences in both growth and vascularity of between *caNrf2^ΔN^*- and *Nrf1/2^+/^^+^*-bearing tumors, the G2/M-phase of the *caNrf2^ΔN^* cell cycle was shortened, along with the S-phase extended (Figure 6H). This implies that G2/M-phase arrest is likely caused by constitutive activation of Nrf2, in agreement with the supportive evidence that inactivation of Nrf2 markedly prolonged the G2/M-phase of *Nrf2^−/^^−ΔTA^* cells (Figure 6H). Relevant gene expression analysis also revealed that *p15, p21* and *Puma* were significantly up-regulated, but *p18*, *CDK1*, *E2F2* and *Bid* were down-regulated by *caNrf2^ΔN^* (Figure 6J and Figure S10A). Conversely, the inactive *Nrf2^−/^^−ΔTA^* mutant still up-regulated expression of *RB1*, *CDK1, E2F3*, and *Cyclin D1* (Figure S10A), but strikingly down- regulated *FTH1* and *FTL* (both encode ferritin heavy and light chains, that are involved in both iron-dependent lipid peroxidation and ferroptosis, in Figure S10G). Overall, these demonstrate that Nrf1α and Nrf2 coordinately regulate certain key genes involved in cell cycle and apoptosis.

### 2.10. Different Subsets of Genes Are Finely Regulated by Nrf1α, Nrf2 Alone or Both

**Figure 7:**
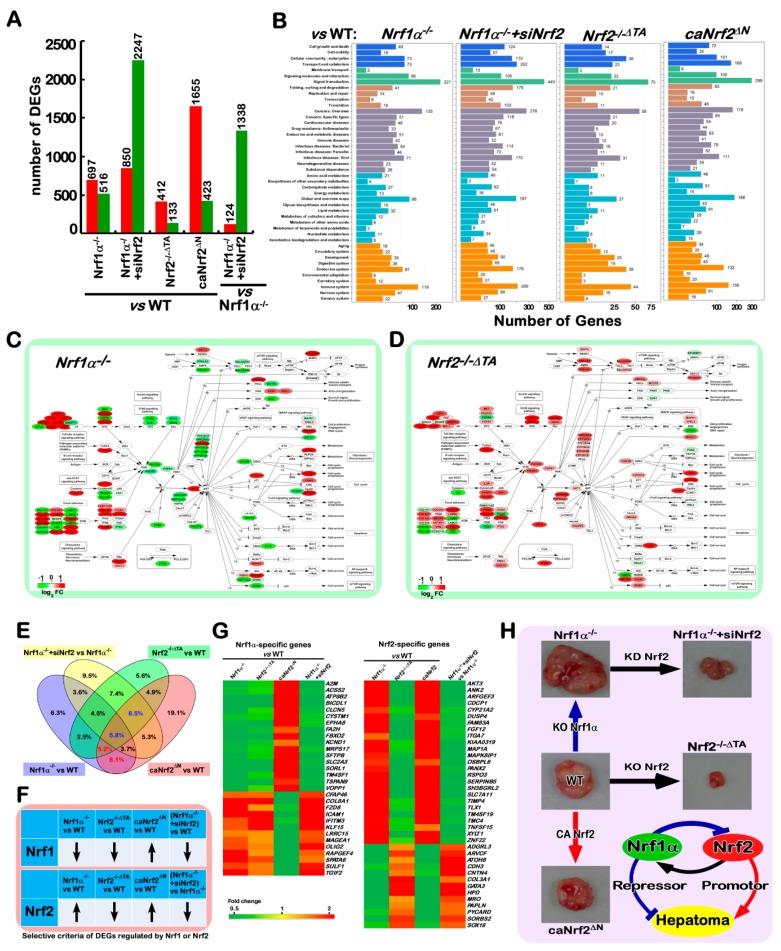
An axiomatic rationale underlying distinct animal xenograft tumor phenotypes. (**A**) Differentially expressed genes (DEGs) in all distinctive cell lines were analyzed by transcriptome sequencing. The differences in the number of DEGs are shown after being compared with wild-type or indicated cell lines. Those increased or decreased DEGs were represented by red or green columns, respectively. The DEGs were selected according to the following criteria: fold change ≥ 2 or ≤ 0.5 and diverge probability ≥ 0.8 (as compared to the control group). (**B**) KEGG classification of DEGs for each pairwise. The X-axis shows the number of DEGs, while the Y-axis represents distinct second- grading KEGG pathways. The top pathways are shown in different colors, such as cellular processes (*blue*), metabolism (*light blue*), environmental information processing (*green*), genetic information processing (*brown*), human disease (*purple*), and organism system (*orange*). (**C**,**D**) Significant differences in the DEGs enriched responsible for the PI3K-AKT signaling pathway in Nrf1α^−^^/^^−^ and Nrf2^−^^/^^−^^Δ^^TA^ cell lines. (**E**) The Venn diagram shows the DEGs in four single variable group. To expand the screening range, the DEG is redefined as a fold change greater than 1.5 or less than 0.66. Nrf1α- or Nrf2-specific downstream genes were indicated by red and green numbers, respectively. (**F**) Distinct changes in abundances of Nrf1α and Nrf2 were illustrated after both protein levels present in each of indicated cell lines was compared with the equivalent values from wild-type cells. (**G**) The Heat maps of particularly Nrf1α- and Nrf2-specific downstream genes, which were screened from the transcriptome data in this experimental setting herein. (**H**) An explicit model is proposed to decipher the axiomatic rationale underlying such distinct animal xenograft tumor phenotypes, demonstrating significant differences in the cancer pathobiology of between Nrf1α and Nrf2.

Nrf1 and Nrf2 are two important CNC-bZIP transcription factors that are widely expressed in various tissues and also regulate seemingly similar expression patterns of ARE-driven downstream genes that have been identified [33,85]. Notably, the ever-accumulating evidence demonstrates that Nrf1 and Nrf2 also exert many different and even opposing functions and, in particular, the unique indispensable functions of Nrf1 are not substituted by Nrf2 [32]. Accordingly, the above-described data unraveled that both CNC-bZIP factors have elicited mutual synergistic and antagonistic roles in regulating the precision expression of cognate genes in distinct cell processes, aiming to maintain the normal cellular homeostasis. Herein, to further evaluate the functional similarities and differences between Nrf1α and Nrf2, the genome-wide expression of genes in *Nrf1/2^+/^^+^*, *Nrf1α^−/^^−^*, *Nrf1α^−/^^−^+ siNrf2*, *Nrf2^−/^^−ΔTA^* and *caNrf2^ΔN^* cells was determined by transcriptome sequencing. Those detectable genes with a fold change ≥ 2 and another diverge probability ≥ 0.8 were defined as differentially expressed genes (DEGs), by comparison with equivalents measured from *Nrf1/2^+/^^+^* cells (Figure 7A). 

Consequently, *Nrf1α^−/^^−^* cells gave rise to 1213 of DEGs (i.e., 697 up-regulated plus 850 down- regulated), but the number of DEGs in *Nrf1α^−/^^−^+siNrf2* cells was significantly increased to 3097 genes, 2247 of which were rather down-regulated by siNrf2 (Figure 7A). Intriguingly, only 545 of DEGs were detected in *Nrf2^−/^^−ΔTA^* cells, implying that many genes are silenced or prevented by the inactive *Nrf2^−/^^−ΔTA^* mutant (distinctive from simple knockout of this factor). These data suggest that, in this regulatory system by the cooperation of Nrf1 and Nrf2, a single change of both has only limited effects on overall gene expression, and thus both changes will have a greater impact. For instance, when compared to those of *Nrf1α^−/^^−^* cells, silencing of Nrf2 caused 124 genes to be up-regulated, and still led 1338 genes to be down-regulated in *Nrf1α^−/^^−^+siNrf2* cells (Figure 7A, last column), such that malignant growth of *Nrf1α^−/^^−^*-derived tumor was repressed by knockdown of Nrf2. Conversely, reinforced expression of Nrf2 (and Nrf1) in *caNrf2^ΔN^* cells led to up-regulation of 1655 genes, albeit 423 genes were still down-regulated. Thus, these findings indicate that Nrf2 is a dominant activator to regulate many genes, while Nrf1α appears to exert dominant negative effects on some genes.

Enrichment analysis revealed that DEGs of *Nrf1α^−/^^−^* cells were subject to 16 pathways (*p* < 0.001), of which 9 are responsible for human disease and 4 are involved in the environmental information processing (Table S1). By contrast, most of cellular processes were significantly changed in *Nrf2^−/^^−ΔTA^* and *Nrf1α^−/^^−^+siNrf2* cells (Tables S2 and S3). Thus, loss of Nrf1α relevant to the disease suggests that its function is essential for maintaining cellular homeostasis, while Nrf2 exerts its greater roles in regulating most of cellular physiological processes. For example, the above-described alterations in the cells cycle of *Nrf1α^−/^^−^+siNrf2* were also validated by transcriptome (Table S2). Further calculation of the DEGs distribution unraveled that signal transduction, cancer-relevant, immune system and metabolism were the most abundant secondary KEGG pathways in Nrf1- or Nrf2-deficient cells (Figure 7B). An insight into the cellular signaling transduction uncovered that the most DEGs are involved in the PI3K-AKT pathway (Table S1). In this pathway, a key tumor suppressor PTEN was significantly and oppositely altered in both *Nrf1α^−/^^−^* and *Nrf2^−/^^−ΔTA^* cell lines (Figure 3F and Figure 7C,D). Based on these specific findings, much-focused DEGs in *Nrf1α^−/^^−^* and *Nrf2^−/^^−ΔTA^* cells were mapped according to the KEGG pathway. The results illustrated that both cell lines displayed significant opposing changes in the PI3K-AKT pathways (Figure 7C,D and Figure S11A). As interested, knockout of *Nrf1α* (with accumulated Nrf2) caused a general reduction in transcription of most AKT-signaling molecules, but they were thus generally increased by inactivation of *Nrf2*. Such striking disparity is dictated by the distinction of Nrf2 proteins in between these two cell lines (Figure 2 and Figure S6). 

Notably, although seemingly similar downstream genes are regulated by Nrf1 and Nrf2, *de facto* activation of Nrf2 by knockout of *Nrf1α* can inevitably cause their opposite effects on some genes against theoretic expectations. This is further evidenced by the results from *Nrf1α^−/^^−^+siNrf2* cells, revealing that many of those accumulated Nrf2’s effects on downstream genes by *Nrf1α^−/^^−^* were strikingly reduced by knockdown of Nrf2. Therefore, by comparison of the DEGs between *Nrf1α^−/^^−^* and *Nrf1α^−/^^−^+siNrf2* cell lines, an opposite expression profiling of 87 genes was uncovered by Nrf2 knockdown (Figure S11B–D). About 24% of these genes are responsible for the metabolism-related enzymes. This implies that the function of Nrf2 is closely related to cellular metabolism, particularly in the absence of Nrf1α. This is further approved by another opposite expression profiling of other 83 DEGs in between *Nrf2^−/^^−ΔTA^* and *caNrf2^ΔN^* cell lines (Figure S12). Still 16% of differential expression genes are related to cellular metabolism, but the other 24% of these genes are involved in signaling transduction. This observation indicates that in the presence of Nrf1α, Nrf2 acts as a major player in cellular signaling cascades, but its role in metabolism appears to be restricted possibly by Nrf1α.

The Venn diagrams illustrated that distinct subsets of DEGs were regulated by Nrf1, Nrf2 alone or both (Figure 7E). The common genes regulated by Nrf1α and Nrf2 were seen by comparison of DEGs in either *Nrf1α^−/^^−^* or *caNrf2^ΔN^* with wild-type. In the intersection of *Nrf1α^−/^^−^* and *caNrf2^ΔN^*, the remaining portions after excluding *Nrf1α^−/^^−^+siNrf2* with *Nrf1α^−/^^−^* were composed of the (*red numbered*) genes closely correlated to regulation by Nrf1α. The genes regulated by Nrf2 were also found by comparison of DEGs in *Nrf1α^−/^^−^+siNrf2* with *Nrf1α^−/^^−^*, as well as *Nrf2^−/^^−ΔTA^* or *caNrf2^ΔN^* with wild-type, such the intersection of these three sets comprised the (*blue numbered)* genes preferably regulated by Nrf2. Furtherly, based on the changes in Nrf1 and Nrf2 proteins detected in distinct cell lines (Figure 7F), we also screened which portions of highly-relevant downstream genes were consistent with or opposite to the changing trends of Nrf1 or Nrf2, respectively. Consequently, 30 of Nrf1α-specific downstream genes were shown (in Figure 7G, *left panel*), amongst which 17 genes were up-regulated and 13 genes were down-regulated. Meanwhile, 38 of Nrf2-specific downstream genes were also found herein, of which 25 were up-regulated and 13 were down-regulated (*right panel*). Collectively, our findings provide an axiomatic rationale for differential expression of different subsets of genes to dictate distinct phenotypes of animal xenograft tumors (Figure 7H). Significantly, the malfunction of Nrf2 is defined as a potent tumor promoter, but it can be efficiently confined or suppressed by Nrf1α that acts as a dominant tumor repressor.

## 3. Discussion

The ever-accumulating evidence has demonstrated that Nrf1 is a key player in the pathogenesis of NASH and HCC, as well as other relevant neurodegenerative diseases and type 2 diabetes [32,33]. However, it should be noted that these experimental mouse genomes were manipulated to delete all Nrf1 isoforms from the single *Nrf1/Nef2l1* gene. In this study, human *Nrf1α*-specific knockout was achieved by its gene-editing so as to create the frameshift mutation. The phenotypes of NASH and malignancies were reconstructed by using the monoclonal *Nrf1α^−/^^−^* cell lines. Thereby, this provides an available model for the follow-up study to elucidate the relevance of Nrf1α with NASH and its malignant transformation into HCC. In the *Nrf1α^−/^^−^*-leading model, the inflammation marker COX2 is constitutively increased, which thus entails a non-resolving feature. By contrast, the development- related COX1 expression was almost completely abolished by *Nrf1α^−/^^−^*. The resultant metabolites of arachidonic acid by the rate-limiting enzyme COX2, that also serves as a direct target of Nrf2 [57,58], are much likely to play a crucial role in development and progression of inflammation, particularly NASH and hepatoma caused by knockout of *Nrf1α^−/^^−^*. 

Further examinations revealed that the *Nrf1α^−/^^−^*-caused increase of COX2 expression occurred by accumulated Nrf2 protein, but both were effectively diminished by two inhibitors of JNK (i.e., SP600125) and mTOR (i.e., rapamycin). Hence, the Nrf2-COX2 pathway is inferable to be regulated by both JNK and mTOR signaling pathways, albeit the detailed mechanisms remain unclear. Herein, we also found that inhibition of the Nrf2-COX2 pathway is accompanied by decreases in AKT, S6K1 and GSK3β. This is consistent with the notion that Nrf2 is regulated by the mTOR-AKT-GSK3β pathway [86]. Our findings also unravel that Nrf2 may be monitored by JNK signaling towards AP-1 pathway, but in turn, some AP-1 components (i.e., Jun, Fra-1) are mediated by Nrf2 insofar as to form a feedback regulatory loop. Contrary to *Nrf1α^−/^^−^*, MEFs of *Nrf1^−/^^−^*^(^^ΔDBD)^, in which almost all DBD (DNA-binding domain)-containing Nrf1 isoforms are disrupted [27,28,51,52], exhibited marked decreases in total COX2 and most of Nrf2 to considerably lower levels, that are roughly similar to those determined in *Nrf2^−/^^−^*^(^^ΔDBD)^ MEFs (Figure S5C). This difference between human *Nrf1α^−/^^−^* and mouse *Nrf1^−/^^−^*^(^^ΔDBD)^ demonstrates Nrf1 isoform-dependent regulation of the Nrf2-COX2 pathway in distinct species as experimented.

Notably, accumulation of free radicals in *Nrf1^−/^^−^*^(^^ΔDBD)^ MEFs results from decreased expression of ARE-driven genes involved in glutathione synthesis, antioxidant and detoxification [87]. Similar, but different, stress caused by liver-specific knockout of *Nrf1^−/^^−^*^(^^ΔDBD)^ activates a subset of Nrf2-dependent ARE-battery genes in mice, but Nrf2 cannot still compensate for the loss of Nrf1’s function leading to NASH and HCC [27,28]. The inducible liver-specific knockout of *Nrf1^−/^^−^*^(^^ΔDBD)^ in mice also increased glutathione levels; this was considered to result from up-regulation of *xCT* (as a component of the cystine/glutamate antiporter system *X_C_^−^*), but with no changes in glutathione biosynthesis enzymes [51]. In the present work, human *Nrf1α^−/^^−^* leads to substantial increases in both ROS and lipid levels, also accompanied by high expression of *xCT* and other ARE-driven genes (e.g., *HO-1, GCLC, GCLM, NQO1*). These genes are Nrf2-dependent because their expression is reduced by inactive *Nrf2^−/^^−ΔTA^* mutant and also repressed by silencing of Nrf2 (in *Nrf1α^−/^^−^+siNrf2* cells). In addition to COX1 and COX2, both Alox5 and FLAP (both also involved in arachidonic acid metabolism) are significantly up-regulated in *Nrf1α^−/^^−^* cells, and also modestly increased in *Nrf2^−/^^−ΔTA^* cells. However, liver-specific *Nrf1^−/^^−^*^(^^ΔDBD)^ mice display no changes in the expression of *COX1, COX2* and *Alox5* [51]. Overall, these discrepancies are likely attributed to the variations of which Nrf1 isoforms have two-sided effects on Nrf2 and diverse downstream genes, depending on different cell types in distinct species. Hence, it is crucially important to determine the *bona fide* effects of Nrf1 and Nrf2 alone or in combination on distinct cognate genes within inter-regulatory networks (Figure 5K).

Albeit Nrf1 and Nrf2 are recruited for directly binding the ARE sites in the promoter regions of *COX1* and *COX2* genes [57,58], our evidence unravels that both CNC-bZIP factors have different or opposing roles in bi-directional regulation of *COX1* and *COX2* by distinct interrelated positive and negative pathways. In particular, Nrf1α may have a two-handed potency to execute as an activator or repressor, depending on distinct cognate genes such as *COX1* and *COX2*, through different regulatory pathways. For example, the regulation of *COX2* is contributed positively by Nrf2 and also negatively by Nrf1α, albeit its promoter-driven *P_COX2_-Luc* reporter is transactivated by Nrf1 and Nrf2. Such direct transactivation by Nrf1 (as well as Nrf2) may be neutralized or counteracted by its dominant-negative effects triggered by other indirect mechanisms (as shown in Figure 5K).

Just contrary to *COX2*, expression of *COX1* is regulated positively by Nrf1α but negatively by Nrf2, albeit no direct activation of its promoter-driven *P_COX1_-Luc* reporter by ectopic Nrf1 and Nrf2 was detected here. In an attempt to explore the mechanisms by which COX1 is indirectly regulated by Nrf1/2, we found that both CNC-bZIP factors can directly activate the expression of miR-22 driven by its ARE site. The miR-22, along with Nrf1 and Nrf2, all inhibit the *PTEN-miR22b-Renilla* reporter activity, implying that these two factors possess an intrinsic ability to suppress the tumor suppressor PTEN through activating miR-22, as consistent with previous reports [76,88]. However, *de facto* endogenous PTEN expression at mRNA and protein levels is almost completely abolished in *Nrf1α^−/^^−^* cells (retaining hyper-active Nrf2), but also dramatically increased in *Nrf2^−/^^−ΔTA^* cells (with decreased Nrf1). Collectively, these findings demonstrate that Nrf2 is a dominant negative to inhibit PTEN; this is further evidenced by a significant reduction of PTEN by a priori constitutive activator of Nrf2 in *caNrf2^ΔN^* cells (also with enhanced Nrf1). By contrast, Nrf1α has the ’Ying-Yang’ two-sided effects on PTEN. On one side, Nrf1α acts as a major positive regulator of PTEN, while on the other side of Nrf1α, it is enabled to exert a minor negative role in PTEN, but this negation could also be concealed by dominant negative Nrf2 or counteracted by the major positive action of Nrf1α *per se*.

Since PTEN is well known to act as the most critical inhibitor of the PI3K-AKT pathway [76,88], thereby, inactivation of PTEN by ROS provokes activation of its downstream PI3K-AKT signaling cascades so as to promote cell survival [89,90]. Notably, the ever-increasing evidence demonstrates that PTEN can direct an inhibitory effect on the expression of ARE-driven genes by inhibiting Nrf2 [17,19]. Taken together with our results from this study, it is demonstrated that the intracellular ROS levels are monitored by Nrf1α- and/or Nrf2-mediating ARE-battery genes, but in turn, expression of Nrf1/2-target genes is also negatively regulated by ROS-activated miR22-PTEN signaling to form a feedback regulatory circuit. Of note, activation of Nrf1/2 by ROS can promote the miR-22 expression, which may serve as an important approach to regulate the PTEN-PI3K-AKT pathway. Thereby, the quantitative regulations of cellular ROS levels are achieved by close cooperation of Nrf1α and Nrf2 coordinately through direct and indirect mechanisms, so as to maintain normal redox homeostasis. Conversely, dysfunction of Nrf1α and Nrf2 (particularly its malfunction) leads to severe redox stress and resultant cancer development possibly by the aberrant PTEN-PI3K-AKT signaling pathway.

It is inferable that almost abolishment of PTEN in malignantly growing *Nrf1α^−/^^−^*-derived tumor cells results principally from an aberrant accumulation of Nrf2 protein, because rescue of PTEN expression occurs after Nrf2 is silenced, so that the existing *Nrf1α^−/^^−^+siNrf2*-derived tumor growth is dramatically repressed by Nrf2 knockdown. In turn, aberrant accumulation of Nrf2 in *Nrf1α^−/^^−^* cells is caused by impaired PTEN expression. This is consistent with the pathology of *PTEN^−/^^−^*-leading cancer, in which the abnormal nuclear accumulation of Nrf2 is caused by an impairment of GSK3β- directed β-TrCP-based proteasome-mediated degradation, as described by [19,20,21]. In addition to an impairment of the GSK3β-directed β-TrCP pathway, aberrant accumulation of Nrf2 is augmented by inhibition of the Keap1-based proteasome-mediated degradation in *Nrf1α^−/^^−^*-derived tumor cells. Noticeably, the Keap1 protein, rather than mRNA, levels are significantly reduced in *Nrf1α^−/^^−^* cells, albeit its binding partner p62, acting as a major regulator of Keap1 to the autophagic degradation [91], is strikingly down-regulated in *Nrf1α^−/^^−^* cells. From this, we postulate that a p62-independent mechanism may account for the Keap1 protein degradation and also is reinforced in *Nrf1α^−/^^−^* cells. However, it cannot be ruled out that the biosynthesis of Keap1 polypeptides may also be retarded during these conditions. 

Several lines of evidence presented herein demonstrate that Nrf2 is predominantly negatively regulated by Nrf1α because *Nrf1α^−/^^−^* enables Nrf2 to be liberated from the confinements by both the PTEN-GSK3β-directed β-TrCP-based and Keap1-based proteasomal degradation pathways (Figure 5K). Consequently, accumulation of Nrf2 leads to aberrant activation of ARE-driven cytoprotective genes (e.g., *HO-1*, *GCLM*, *NQO1*) in so much as to shelter or promote *Nrf1α^−/^^−^*-driven tumor cells. In fact, These ARE-battery genes can be directly activated by Nrf1α, but some of these downstream genes could also be inhibited through braking control of the Nrf2 activity. Overall, distinct levels of Nrf1 alone or in cooperation with Nrf2 finely tune and also quantitatively regulate the expression of diverse downstream genes in order to meet different cellular needs (Figure 4L and Figure 5K). Thereby, these resulting collective effects determine distinctions in phenotypes of animal xenograft tumor models as deciphered in this study (Figure 7H). Consistently, malignant growth of *Nrf1α^−/^^−^*-derived tumor is substantially suppressed by knockdown of Nrf2, by comparison with *Nrf1α^−/^^−^+siNrf2*-derived tumor. Conversely, almost no solid tumor is formed in those nude mice that have been inoculated by injecting the inactive *Nrf2^−/^^−ΔTA^*-derived cells, albeit Nrf1 is modestly decreased along with loss of Nrf2’s function. These demonstrate that Nrf1α acts as a dominant tumor suppressor principally by confining the oncogenicity of Nrf2. In turn, albeit Nrf2 exerts a dominant tumor-promoting role in tumorigenesis and malignant growth, it can also directly mediate the *Nrf1* gene transcription to form a feedback regulatory loop. This is validated by further evidence revealing that, upon the presence of Nrf1 in *caNrf2^ΔN^*-derived tumor cells, its growth is almost unaffected by constitutive activation of Nrf2, as well as antioxidant and detoxifying genes, when compared with equivalents of wild-type *Nrf1/2^+/^^+^*-bearing tumor. 

In an attempt to clarify those seemingly contradictory results obtained from loss of Nrf1α and its functional gain (i.e., ectopic over-expression), we have also surprisingly found that there exists a mutual inter-regulatory relationship between Nrf1α and Nrf2, thereby enabling both factors to elicit opposing and unifying roles in regulating distinct subsets of downstream genes (particularly ARE- driven cognate genes). Importantly, we have discovered that that forced expression of Nrf1 enables the Nrf2 protein to be reduced, whereas loss of Nrf1α leads to a significant increase in Nrf2 protein, but not its mRNA levels (Figure 4L). By sharp contrast, both mRNA and protein levels of Nrf1 are increased by over-expression of Nrf2 or its constitutive activation of *caNrf2^ΔN^*, but also repressed by inactivation of Nrf2. Further experimental evidence has unraveled no activation of the human *Nrf2* promoter-driven *P_Nrf2_-Luc* reporter mediated by Nrf1 or Nrf2, albeit mouse *Nrf2* gene was activated by its ARE sites as described [92]. Notably, the human *Nrf1* promoter-driven *P_Nrf1_-luc* reporter is *trans*-activated by Nrf1 (at the locus Site-2) and Nrf2 (at the locus Site-1), respectively (Figure 5 and Figure S8A). These findings demonstrate there are, at least, two distinct levels (i.e., transcript and protein abundances) at which Nrf1α and Nrf2 have cross-talks with each other to influence the expression of ARE-driven genes. Thereby, synergistic or antagonistic effects of Nrf1α and Nrf2 depend on mutual competition or somehow coordination with spatiotemporally binding to the same or different ARE enhancers within their downstream genes. Overall, such inter-regulatory cross-talks between Nrf1α and Nrf2 may be a vitally important strategy for the precision regulation of distinct downstream genes. This rationale can provide a better explanation of those complicated physio- pathological functions with distinct disease phenotypes exhibited in different models, as described [32,93]. 

Importantly, a hot controversy surrounds dual opposing roles of Nrf2 in the pro- or anti-cancer contexts, termed ‘the Nrf2 paradox’ [93,94]. This study has defined that function of Nrf2 is dictated by activation or inactivation of Nrf1α. This is because deterioration of *Nrf1α^−/^^−^*-tumor results from hyper-active Nrf2, along with decreased PTEN and activation of downstream PI3K-AKT signaling, but the *Nrf1/2^+/^^+^*-tumor growth is unaffected by constitutive activation of Nrf2 when compared with *caNrf2^ΔN^*-tumor. Consistently, it has been recently showed that Nrf2 also acts as a tumor-promoting player, depending upon aberrant activation of the PI3K-AKT signaling pathway, albeit it serves as a tumor-preventing player by activating ARE-driven cytoprotective genes under normal activation conditions [21]. As such, a similar subset of ARE-driven genes is also highly expressed in *Nrf1α^−/^^−^* and *caNrf2^ΔN^* cell lines. However, our findings demonstrate that the tumor-promoting role of Nrf2 is determined by loss of Nrf1α function, independent of those cytoprotective gene expressions. Even as a dominant braking control of Nrf2 activity, Nrf1α may play an essential role for ’decision-maker’ or ‘executor’ in the cell senescence and cancer progression, since a secretory phenotype of senescent cells occurs by a Nrf2-independent mechanism [95], albeit the relevance to Nrf1 needs to be verified.

In summary, this study provides a panoramic view of the mutual inter-regulatory cross-talks between Nrf1α and Nrf2 in order to determine quantitative expression of distinct downstream genes that are involved in different patho-physiological processes. Significantly, the axiomatic rationale underlying distinct animal xenograft tumor phenotypes has also been unraveled by transcriptomic analysis of the genome-wide gene expression in *Nrf1α^−/^^−^*, *Nrf1α^−/^^−^+siNrf2*, *Nrf2^−/^^−ΔTA^* and *caNrf2^ΔN^* cell lines, when compared with wild-type *Nrf1/2^+/^^+^*. Notably, an overwhelming majority of the PTEN- directed PI3K-AKT signaling cascades are strikingly activated in *Nrf1α^−/^^−^*, but rather repressed in *Nrf2^−/^^−ΔTA^* cells. Silencing of Nrf2 leads to opposing expression of 87 genes in between *Nrf1α^−/^^−^* and *Nrf1α^−/^^−^+siNrf2* cell lines. Although most of cognate genes are, to different extents, co-regulated by Nrf1α and Nrf2, this study has highlighted about 30 of Nrf1α-specific downstream genes, and 38 of Nrf2-specific downstream genes (Figure 7G). Among Nrf1α-regulated genes, those encoding A2M, EPHA8, FBXO2, KCND1, SLC2A3, SORL1, OLIG2, and RAPGEF4 should be responsible for the nervous system, although it is unclear whether they are relevant to those phenotypes of *Nrf1α^−/^^−^* -leading neurodegenerative diseases as reported by [34,35]. Only expression of *ACSS2, FA2H,* and *KLF15* genes are associated with lipid metabolism, but it is required to determine their roles in relevant phenotypes, as described by [27,36,40,41]. By contrast, a portion of Nrf2-specific genes are critical for the development of various tissues and organs, neurons and cardiomyocytes, but none of the specific physio-pathological phenotypes in the *Nrf2^−/^^−ΔDBD^* mice are observed, implying that their functions can be compensated by Nrf1 or other factors. As such, the other Nrf2-specific genes may be involved in development, movement and adhesion of epithelial cells, but it is unknown whether these gene functions enable Nrf2 to be endowed with its potent tumor-promoting roles in cancer progression and metastasis. 

## 4. Materials and Methods

### 4.1. Cell lines, Culture and Transfection

All four cell lines *Nrf1α^−/^^−^*, *Nrf1α^−/^^−^*+siNrf2, *Nrf2^−/^^−^^ΔTA^* and *caNrf2^ΔN^* were created in this study. Their progenitor cells are the human hepatocellular carcinoma (HepG2) or another non-cancerous human liver (HL7702) cell lines. The latter two lines HepG2 and HL7702 are wild-type (*Nrf1/2^+/^^+^*, *Keap1^+/^^+^*) cells, because not any mutants in the *Nrf1*, *Nrf2* and *Keap1* genes are therein confirmed by sequencing. However, it is important to note that Nrf1 and its long TCF11 isoform are co-expressed at a ratio of 1:1 in HL7702 cells. By contrast, significantly decreased expression of Nrf1 is observed in HepG2 cells (Ren et al., 2016), while almost no expression of its longer TCF11 transcripts were detected. For relevant identification of these cell lines, see Figure 1 and Figures S1 and S9.

Experimental cells were allowed for growth in DMEM supplemented with 5 mM glutamine, 10% (v/v) foetal bovine serum (FBS), 100 units/mL of either of penicillin and streptomycin, in the 37 °C incubator with 5% CO_2_. The cells were transfected with indicated plasmids alone or in combination for 8 h, using Lipofectamine® 3000 Transfection Kit (Invitrogen, Carlsbad, CA, USA), and then allowed for 24-h recovery from transfection in the fresh medium before being subjected to indicated experiments.

### 4.2. Expression Constructs and other Oligos Used for siRNA and miRNA

Expression constructs for human Nrf1, Nrf2, JUN and FOS were made by cloning each of their full-length cDNA sequences into a pcDNA3 vector, respectively. The other plasmids specifically for the genome-editing of *Nrf1* or *Nrf2* by Talens or CRISPR/Cas9 were created and identified (as shown in Figure 1 and Figures S1 and S9). Further, we also made four specific luciferase reporters, which were driven by distinct gene promoter regions from the human *Nrf1*, *Nrf2*, *COX1* and *COX2*. Different lengths of these gene promoter regions were amplified by PCR from their genomic loci and inserted into the PGL3-basic vector. In addition to these intact reporter genes *P_Nrf1_-Luc, P_Nrf2_-Luc, P_COX1_-Luc, P_COX2_-Luc* and *miR22-ARE-Luc*, all these relevant ARE-specific mutant reporters were engineered. Moreover, double fluorescent reporters (i.e., *PTEN-miR22b* and *COX1-miR22b*) were also created by cloning the 3’ UTR region sequences of *COX1* and *PTEN*, that were amplified from reverse transcription PCR products and then ligated into the psiCHECK2 vector. All primers and other oligos used for siRNAs and miR-RNAs (Table 1) were synthesized by Sangon Biotech (Shanghai, China). The fidelity of all constructs used in this study was confirmed to be true by sequencing.

### 4.3. Subcutaneous Tumor Xenografts in Nude Mice

Mouse xenograft models were here made by subcutaneous heterotransplantation of the human hepatoma HepG2 (i.e., *Nrf1/2^+/^^+^* or each derivate of *Nrf1α^−/^^−^*, *Nrf1α^−/^^−^*+siNrf2, *Nrf2^−/^^−ΔTA^* and *caNrf2^ΔN^* cell lines into nude mice, as described [96]. Experimental cells (1 × 10^7^) were allowed for growth in the exponential phase) and then suspended in 0.2 mL of serum-free DMEM, before being inoculated subcutaneously into the right upper back region of male nude mice (BALB/C*nu*/*nu*, 6 weeks, 18 g, from HFK Bioscience, Beijing, China) at a single site. The procedure of injection into all experimental mice was completed within 30 min, and subsequent formation of the subcutaneous tumour exnografts was observed. Once the tumor exnografts emerged, their sizes were successively measured once every two days, until the 32nd day when these mice were sacrificed and their transplanted tumors were excised. The sizes of growing tumors were calculated by a standard formula (i.e., V = ab^2^/2) and then are shown graphically (*n* = 6 per group). Thereafter, the tumor tissues were also subjected to the histopathological examination by the routine hematoxylin-eosin staining. 

Notably, all the relevant animal experiments in this study were indeed conducted according to the valid ethical regulations that have been approved. All mice were maintained under standard animal housing conditions with a 12-h dark cycle and allowed access *ad libitum* to sterilized water and diet. All relevant studies were carried out on 8-week-old male mice (with the license No. PIL60/13167) in accordance with the United Kingdom Animal (Scientific Procedures) Act (1986) and the guidelines of the Animal Care and Use Committees of Chongqing University and the Third Military Medical University, both of which were subjected to the local ethical review (in China). All relevant experimental protocols were approved by the University Laboratory Animal Welfare and Ethics Committee (with two institutional licenses SCXK-PLA-20120011 and SYXK-PLA-20120031). 

### 4.4. Histology and Immunohistochemistry

The xenograft tumor tissues were immersed in 4% paraformaldehyde overnight before being transferred to 70% ethanol. Individual tumor tissues were placed in the processing cassettes, dehydrated through a serial of alcohol gradient, and embedded in paraffin wax blocks. These, paraffin-embedded samples were then sectioned into a series of 5-μm-thick slides. Before staining, the tissue sections were de-waxed in xylene, rehydrated through decreasing concentrations of ethanol, and washed in PBS. Lastly, they were stained with routine hematoxylin and eosin (H&E), and visualized by microscopy. For immunohistochemical staining, the slides of tumor tissues were de-paraffinized in a solution of xylene and then dehydrated in the concentration-graded ethanol before inactivation of endogenous peroxidase activity. Subsequently, the samples were allowed for boiling in microwave for 15 min in a citrate buffer (pH 6.0) so to retrieve antigen, and then blocked within 1% BSA for 60 min. Thereafter, the sample sections were incubated with the primary antibodies against CD31 (dilution 1:100) at 4 °C overnight, and then re-incubated for 60 min with the biotin-conjugated secondary antibody at room temperature, before being visualized by DAB staining. The resultant images presented were acquired under a light microscope (Leica DMIRB, Leica, Frankfurt, Germany) equipped with a DC350F digital camera.

### 4.5. Immunocytochemistry and Confocal Microscopy

Experimental cells (2 × 10^5^) that had been allowed for 24-h growth on a cover glass placed in each of 6-well plates, the cells were fixed for 15 min with 4% paraformaldehyde. The cells were permeabilized for 10 min with 0.1% Triton X-100 in PBS, before immunocytochemistry with the primary antibodies against Nrf1 (dilution 1:50) and Nrf2 (dilution 1:500) incubated at 4 °C overnight. The immunostained cells were then visualized by further incubation with the Alexa Fluor 488- conjugated goat anti-rabbit IgG (dilution 1:200) for 1 h at room temperature in the dark, followed by DAPI staining of the nuclear DNAs for 5 min. The resulting fluorescence images were observed and photographed under a confocal microscope (Leica).

### 4.6. Subcellular Fractionation

Equal numbers (1 × 10^6^) of different cell lines were seeded into each of 6-cm dishes and allowed for growth for 24 h before being harvested by incubation with ice cold Nuclei EZ lysis buffer (1 mL added to each dish). The lysates were subjected to subcellular fractionation by centrifuging at 500× *g* for 5 min at 4 °C. The supernatants were collected as the non-nuclear cytoplasmic fractions, while the sediment were subsequently washed with the above lysis buffer for two times, each time when 0.5 mL of the nuclei EZ lysis buffer was added into the sediment. The final nuclear pellets were collected by centrifuging at 500× *g* for 5 min at 4 °C. These fractions were then evaluated by Western blotting.

### 4.7. Lipid Staining

Experimental cells were seeded in 6-well plates and cultured in a medium containing 200 μM sodium oleate (Solarbio, Beijing, China). The cells were fixed for 30 min with 4% paraformaldehyde (AR1068, Boster Biological Technology, Wuhan, China) and then stained for 30 min with a solution of 3 g/L oil red O (A600395, Sangon Biotech, Shanghai, China). The stained cells were rinsed 3 times with 60% of isopropyl alcohol (Kelong, Chengdu, China) before the red lipid droplets were visualized by microscopy.

### 4.8. Intracellular ROS staining 

Experimental cells were allowed for growth to an appropriate confluence in 6-well plates and then incubated in a serum-free medium containing 10 μmol/L of 2′,7′-Dichlorodihydrofluorescein diacetate (DCFH-DA) (S0033, Beyotime, Shanghai, China) at 37 °C for 20 min. Thereafter, these cells were washed three times with a fresh serum-free medium, before the green fluorescent images were achieved by microscopy.

### 4.9. Luciferase Reporter Assay

Equal numbers (1.0 × 10^5^) of experimental cells were seeded into each well of the 12-well plates. After reaching 80% confluence, the cells were transfected by using a Lipofectamine 3000 mixture with luciferase plasmids alone or plus other expression plasmids. In the pGL3 plasmid system, the Renilla expression by pRL-TK plasmid serves as an internal control for transfection efficiency. And in the psi-CHECK2 plasmid system, the Pyralis-luciferase activity is also an internal control, while the Renilla-luciferase activity is the experimental test object. The luciferase activity was measured by the dual-luciferase reporter assay system (E1910, Promega, Madison, WI, USA). The resultant data were normalized and calculated as a fold change (mean ± S.D) relative to the activity of the control group (at a given value of 1.0). All the data presented in this study represent at least three independent experiments undertaken on separate occasions that were each performed in triplicate. Significant differences in the transcriptional activity were subjected to statistical analysis.

### 4.10. Real-Time Quantitative PCR

Experimental cells were subjected to isolation of total RNAs by using the RNAsimple Kit (Tiangen Biotech Co., Beijing, China). Then, 500 ng of total RNAs were added in a reverse-transcriptase reaction to generate the first strand of cDNA (with the Revert Aid First Strand Synthesis Kit from Thermo, Waltham, MA, USA). The synthesized cDNA was served as the template for qPCR, in the GoTaq® qPCR Master Mix (from Promega,), before being deactivated at 95 °C for 10 min, and then amplified by 40 reaction cycles of the annealing at 95 °C for 15 s and then extending at 60 °C for 30 s. The final melting curve was validated to examine the amplification quality, whereas the mRNA expression level of β-actin served as an optimal internal standard control.

### 4.11. Western Blotting

Experimental cells were harvested in a lysis buffer (0.5% SDS, 0.04 mol/L DTT, pH 7.5), which was supplemented with the protease inhibitor cOmplete Tablets EASYpack or phosphatase inhibitor PhosSTOP EASYpack (either one tablet per 10 mL of lysis buffer, Roche, Mannheim, Germany). The lysates were denatured immediately at 100 °C for 10 min, sonicated sufficiently, and diluted in 3× loading buffer (187.5 mmol/L Tris-HCl, pH 6.8, 6% SDS, 30% Glycerol, 150 mmol/L DTT, 0.3% Bromphenol Blue) at 100 °C for 5 min. Subsequently, equal amounts of protein extracts were subjected to separation by SDS-PAGE containing 4–15% polyacrylamide, and subsequent visualization by Western blotting with distinct antibodies as indicated. On some occasions, the blotted membranes were stripped for 30 min and then re-probed with additional primary antibodies. β-actin served as an internal control to verify equal loading of proteins in each of electrophoretic wells.

### 4.12. Flow Cytometry Analysis of Cell Cycle and Apoptosis 

Experimental cells (5 × 10^5^) were allowed for growth in 60-mm cell culture plate for 48 h and synchronization by 12-h starvation in a serum-free medium, before being treated with 10 μmol/L BrdU for 12 h. The cells were fixed for 15 min with 100 μL of BD Cytofix/Cytoperm buffer (containing a mixture of the fixative paraformaldehyde and the detergent saponin) at room temperature and permeabilized for 10 min with 100 μL of BD Cytoperm permeabilization buffer plus (containing fetal bovine serum as a staining enhancer) on ice. Thereafter, the cells were re-fixed and treated with 100 μL of DNase (at a dose of 300 μg/mL in PBS) for 1 h at 37 °C, in order to expose the incorporated BrdU, followed by staining with FITC (fluorescein isothiocyanate) conjugated anti-BrdU antibody for 60 min at room temperature. Subsequently, the cells were suspended in 20 μL of 7-amino-actinomycin D solution for 20 min of the DNA staining and re-suspended in 0.5 mL of a staining buffer (i.e., 1 × DPBS containing 0.09% sodium azide and 3% heat-inactivated FBS), prior to the cell cycle analysis by flow cytometry. Furthermore, additional fractions of cells (5 × 10^5^) were allowed for 48-h growth in 60-mm cell culture plate before being used for apoptosis analysis. The cells were pelleted by centrifuging at 1000× *g* for 5 min and washed by PBS for three times, before being incubated for 15 min with 5 μL of Annexin V-FITC and 10 μL of propidium iodide (PI) in 195 μL of the binding buffer, prior to flow cytometry analysis of cell apoptosis. The results were further analyzed by the FlowJo 7.6.1 software (FlowJo, Ashland, OR, USA) before being presented.

### 4.13. Key Resources Used for ’Wet Experiments’ 

Key resources used for ’Wet Experiments’ are shown in Table 1.

### 4.14. The Genome-Wide Transcriptomic Analysis

Total RNAs were subjected to the transcriptomic sequencing by the Beijing Genomics Institute (BGI, www.genomics.org.cn) on the platform of BGISEQ-500 (contract No. is F17FTSCCWLJ1161). After removing the ‘dirty’ raw reads with data filtering, the clean reads were generated and mapped to the reference by using both HISAT [100] and Bowtie2 [101] tools. Of note, gene expression levels were calculated by using the FPKM (Fragments Per Kilobase of exon model per Million mapped fragments) method combined with RSEM [102]. Then, differentially expressed genes (DEGs) were identified with the criteria Fold-change ≥2 and another diverge probability ≥0.8 by using the NOISeq tool [103]. For the functional annotation, all DEGs were mapped to the gene ontology (GO) terms in the database (http://www.geneontology.org/) and the pathway enrichment analysis of DEGs was also performed by using KEGG software (Kanehisa Laboratories, Kyoto, Japan) [104].

### 4.15. Statistical Analysis

Significant differences were statistically determined using the Student’s *t*-test and Multiple Analysis of Variations (MANOVA), except for somewhere indicated. The data are here shown as a fold change (mean ± S.D.), each of which represents at least three independent experiments that were each performed in triplicate.

## 5. Conclusions

Altogether, our present study demonstrates that the malfunction of Nrf2 is defined as a tumor promoter, but it is predominantly suppressed by Nrf1α, that acts as a dominant tumor repressor, specifically through transcriptional regulation of the 26S proteasome-mediated Nrf2 degradation pathways. This complicated process is governed by endogenous inter-regulatory networks between Nrf1α and Nrf2 from multiple signaling pathways towards distinct gene expression. On the inside, there exist mutual opposing and unifying cross-talks between Nrf1α and Nrf2 at distinct levels (i.e., transcript and protein). Notably, Nrf2 can also directly mediate the transcription of the *Nrf1* gene to form a coupled positive and negative feedback circuit, in order to quantitatively monitor both Nrf1 and Nrf2 functioning towards precision expression of distinct downstream genes. This is evidenced by such observations that the malignant growth of *Nrf1α^−/^^−^* -derived tumor is almost prevented by silencing of Nrf2, and *Nrf1α^+/^^+^*-tumor growth is also repressed by the inactive *Nrf2^−/^^−ΔTA^,* but almost unaltered by constitutive activation of *caNrf2^ΔN^* in the presence of Nrf1α. Further evidence has been provided revealing that the hyperactivation of Nrf2 by *Nrf1α^−/^^−^* results from substantial decreases in the expression of Keap1, PTEN and most of 26S proteasomal subunits. Therefore, in view of mutual inter-regulation by between Nrf1α and Nrf2, it should hence be taken severe cautions to interpret the experimental results from loss of Nrf1α, Nrf2 or both, as well as the other data obtained from gains of their functions. Meanwhile, this also poses a great challenge to re-interpret or re-evaluate those relevant data that had been previously published in the past two decades.

## Figures and Tables

**Table 1:** The key resources used in this work.

Reagent or Resource	Source	Identifier
Antibodies		
AKT1	Abcam	ab32505
ALOX5	Sangon Biotech	D220061
CD31	Servicebio	GB11063-3
COX1	Sangon Biotech	D260197
COX2	Abcam	ab62331
Fos	Abcam	ab134122
Fra1	Abcam	ab124722
GCLM	Abcam	ab126704
GSK3β	Sangon Biotech	D160468
HIF1α	Abcam	ab51608
Histone 3	Bioss	bs-0349R
HO-1	Abcam	ab52947
JNK (Anti-JNK1+JNK2+JNK3)	Abcam	ab208035
Jun	Proteintech	10024-2-AP
KEAP1	Sangon Biotech	D154142
NQO1	Abcam	ab80588
Nrf1	Zhang’s [97]	N/A
Nrf2	Abcam	ab62352
p-JNK (Anti-JNK1+JNK2+JNK3 (phospho T183+T183+T221))	Abcam	ab124956
p-S6K1( Anti-RPS6KB1(Phospho-Thr389/412))	Sangon Biotech	D151473
PTEN	Abcam	ab32199
Ubiquitin	Cell Signaling Technology	3933S
Alexa Fluor 488 - Conjugated Goat anti-rabbit IgG	ZSGB-BIO	ZF-0511
α-Tubulin	Beyotime	AF0001
β-actin	ZSGB-BIO	TA-09
β-TrCP	Sangon Biotech	D154110
Biological Samples: Cell Lines		
HepG2	Cell bank of the Chinese Academy of Sciences	TCHu72
*Nrf1α^-/-^*	this paper	NA
*Nrf2^-/-^* *^ΔTA^*	this paper	NA
*caNrf2^ΔN^*	this paper	NA
HepG2^Keap1-/-^	this paper	NA
HL7702	Cell bank of the Chinese Academy of Sciences	GNHu 6
HL7702^Nrf1α-/-^	this paper	NA
MEF	courtesy of Akira Kobayashi	NA
MEF^Nrf1-/-(^^ΔDBD)^	courtesy of Akira Kobayashi	NA
MEF^Nrf2-/-(^^ΔDBD)^	courtesy of John D. Hayes	NA
MEF^Keap1-/-^	courtesy of John D. Hayes	NA
Chemicals, Peptides, and Recombinant Proteins		
12-O-Tetradecanoylphorbol-13-acetate (TPA)	Beyotime	S1819
BAPTA-Acetoxymethyl ester (BAPTA-AM)	Cayman Chemical	15551
Caffeic Acid Phenethyl Ester (CAPE)	Selleck	S7414
cOmplete Tablets EASYpack	Roche	4693116001
cycloheximide (CHX)	Solarbio	C8030
H-89	Beyotime	S1643
JSH-23	Selleck	S7351
MG132	Sigma Aldrich	M7449
oil red O	Sangon Biotech	A600395
paraformaldehyde	Boster Biological Technology	AR1068
PhosSTOP EASYpack	Roche	4906845001
Rapamycin (RAPA)	Sigma Aldrich	37094
sodium oleate	Solarbio	N/A
SP600125	Selleck	S1460
SR11302	Cayman Chemical	11302
*tert*-Butylhydroquinone (tBHQ)	Sigma Aldrich	112941
Thapsigargin (TG)	Sangon Biotech	A616759
Vitamin C (VC)	Sigma Aldrich	33034
Deposited Data		
Oligonucleotides for siRNA or miRNA		
siNrf2 FW	Sangon Biotech	GUAAGAAGCCAGAUGUUAAdTdT
siNrf2 REV	Sangon Biotech	UUAACAUCUGGCUUCUUACdTdT
siJUN FW	Sangon Biotech	GCAUGGACCUAACAUUCGAdTdT
siJUN REV	Sangon Biotech	UCGAAUGUUAGGUCCAUGCdTdT
siFra1 FW	Sangon Biotech	CAAACUGGAAGAUGAGAAAdTdT
siFra1 REV	Sangon Biotech	UUUCUCAUCUUCCAGUUUGdTdT
has-miR-22-3p FW	Sangon Biotech	AAGCUGCCAGUUGAAGAACUGU
has-miR-22-3p REV	Sangon Biotech	AGUUCUUCAACUGGCAGCUUUU
Normal control FW	Sangon Biotech	UUCUCCGAACGUGUCACGUdTdT
Normal control REV	Sangon Biotech	ACGUGACACGUUCGGAGAAdTdT
Oligonucleotides for qPCR		
ALOX5 FW	Tsingke	GCTGCCCCAGCCAGATGGACTC
ALOX5 REV	Tsingke	CTGCTTGGTGTGGAAATGCTGA
COX1 FW	Tsingke	CGCCAGTGAATCCCTGTTGTT
COX1 REV	Tsingke	AAGGTGGCATTGACAAACTCC
COX2 FW	Tsingke	AAGTCCCTGAGCATCTACGGTTT
COX2 REV	Tsingke	GTTGTGTTCCCTCAGCCAGATT
FLAP FW	Tsingke	TCAGCGTGGTCCAGAATGG
FLAP REV	Tsingke	GCAAGTGTTCCGGTCCTCT
FOS FW	Tsingke	CACCGACCTGCCTGCAAGAT
FOS REV	Tsingke	GCTGGGAACAGGAAGTCATCAA
FOSB FW	Tsingke	GCTGCAAGATCCCCTACGAAG
FOSB REV	Tsingke	ACGAAGAAGTGTACGAAGGGTT
Fra1 FW	Tsingke	CCTGCCGCCCTGTACCTTGT
Fra1 REV	Tsingke	GTCTCCGCTGCTGCTGCTACTC
Fra2 FW	Tsingke	CACCATCAACGCCATCACGA
Fra2 REV	Tsingke	CGACGCTTCTCCTCCTCTTCAG
GCLC FW	Tsingke	TCAATGGGAAGGAAGGTGTGTT
GCLC REV	Tsingke	TTGTAGTCAGGATGGTTTGCGA
GCLM FW	Tsingke	GTGTGATGCCACCAGATTTGAC
GCLM REV	Tsingke	CACAATGACCGAATACCGCAGT
HO-1 FW	Tsingke	CAGAGCCTGGAAGACACCCTAA
HO-1 REV	Tsingke	AAACCACCCCAACCCTGCTAT
JUN FW	Tsingke	ATGGAAACGACCTTCTATGACGA
JUN REV	Tsingke	CGTTGCTGGACTGGATTATCA
JUNB FW	Tsingke	AGCCACCTCCCGTTTACACCAA
JUNB REV	Tsingke	ACGGTCTGCGGTTCCTCCTTGA
JUND FW	Tsingke	ATCGACATGGACACGCAGGAGC
JUND REV	Tsingke	GCTGTTGACGTGGCTGAGGACT
KEAP1 FW	Tsingke	AACAACTCGCCCGACGGCAACAC
KEAP1 REV	Tsingke	CATCCCGCTCTGGCTCATACCTC
LPIN1 FW	Tsingke	TGACCAATCGCCAACTCTGG
LPIN1 REV	Tsingke	TCAGCACCAAGATGTCGGCT
mir-22 FW	Tsingke	GCAAGCTGCCAGTTGAAG
mir-22 REV	Tsingke	GTGCAGGGTCCGAGGT
mir-22-RT	Tsingke	GTCGTATCCAGTGCAGGGTCCGAGGTATTCGCACTGGATACGACACAGTT
NQO1 FW	Tsingke	AAGAAGAAAGGATGGGAGGTGG
NQO1 REV	Tsingke	GAACAGACTCGGCAGGATACTGA
Nrf1 FW	Tsingke	TGGAACAGCAGTGGCAAGATCTCA
Nrf1 REV	Tsingke	GGCACTGTACAGGATTTCACTTGC
Nrf2 FW	Tsingke	AATTGCCTGTAAGTCCTGGTCAT
Nrf2 REV	Tsingke	TCATTGAACTGCTCTTTGGACAT
Nrf2^-/-ΔTA^ FW	Tsingke	CGACGGAAAGAGTATGAGCTGGA
Nrf2^-/-ΔTA^ REV	Tsingke	ACTGGTTTCTGACTGGATGTGCT
PGC1βFW	Tsingke	TGGTGAGATTGAGGAGTGCGA
PGC1βREV	Tsingke	GCCTTGTCTGAGGTATTGAGGTATTC
PSMB6 FW	Tsingke	TCAAGAAGGAGGGCAGGTGT
PSMB6 REV	Tsingke	GTAAAGTGGCAACGGCGAA
PTEN FW	Tsingke	TTTGAAGACCATAACCCACCAC
PTEN REV	Tsingke	ATTACACCAGTTCGTCCCTTTC
β-actin FW	Tsingke	CATGTACGTTGCTATCCAGGC
β-actin REV	Tsingke	CTCCTTAATGTCACGCACGAT
Oligonucleotides for construct		
COX1-LUC FW	Tsingke	GCCTCGGTACCCTGCCTGCTCTCTC
COX1-LUC REV	Tsingke	GATGAGAAGCTTACTACTCCTCAGACAGATC
COX1-UTR FW	Tsingke	GCAGGAAAGCAGCATTCTCGAGGGGAGAGCTTTGTGCTTGTC
COX1-UTR REV	Tsingke	CACTGATTAAAAGTCCCTCGCGGCCGCTAAAGTGCTTGTGTC
COX1-UTR-M FW	Tsingke	GTCTTGACTCATGTTTCTCATGAAGCTAATAAAATTCGC
COX1-UTR-M REV	Tsingke	AGCTTCATGAGAAACATGAGTCAAGACCTGGATG
COX2-LUC FW	Tsingke	CTACAAATTGAGGTACCTGGTGTAG
COX2-LUC REV	Tsingke	AATTGGAAGCTTACCGAGAGAACCTTC
COX2-LUC-M FW	Tsingke	GAGCAGATATACAGCCTATTAAGCGTATTAACTAAAACATAAAACATGTCAGCC
COX2-LUC-M REV	Tsingke	GGCTGACATGTTTTATGTTTTAGTTAATACGCTTAATAGGCTGTATATCTGCTC
FOS FW	Tsingke	GCTTTGCCTAAGCTTCACGATGATGTTCTCG
FOS REV	Tsingke	TTCCCTGAATTCTCACAGGGCCAGCAGCGTG
JUN FW	Tsingke	CACGTGAAGCTTCGGACTGTTCTATGACTGC
JUN REV	Tsingke	CGACGGTCTGAATTCAAAATGTTTGCAACTG
Keap1 sgRNA FW	Tsingke	AAACACCGTATGAGCCAGAGCGGGATG
Keap1 sgRNA REV	Tsingke	CTCTAAAACCATCCCGCTCTGGCTCATA
MIR-22-LUC FW	Tsingke	CAGTCCTCTGGGTTGAACAGAGCTATCTCAGACAGAGGAAGGTCGGACGGA
MIR-22-LUC REV	Tsingke	GATCTCCGTCCGACCTTCCTCTGTCTGAGATAGCTCTGTTCAACCCAGAGGACTGGTAC
MIR-22-LUC-M FW	Tsingke	CAGTCCTCTGGGTTGAACAGAAATATTTCAGACAGAGGAAGGTCGGACGGA
MIR-22-LUC-M REV	Tsingke	GATCTCCGTCCGACCTTCCTCTGTCTGAAATATTTCTGTTCAACCCAGAGGACTGGTAC
Nrf1 FW	Tsingke	CGGGGTACCATGCTTTCTCTGAAGAAATACTTAACGGAAGG
Nrf1 REV	Tsingke	GCTCTAGACACTTTCTCCGGTCCTTTGGCTTCC
Nrf1-LUC-#1 FW	Tsingke	CCTAGGCCTGCTAGCGCGACTGAGTTTGTCTCTACACCT
Nrf1-LUC-#1 REV	Tsingke	CTTCAGAGAAAAGCTTGCTGAAGGACCAGAATGTTTATGCT
Nrf1-LUC-#2 FW	Tsingke	CCTAGGCCTGCTAGCGCGACTGAGTTTGTCTCTACACCT
Nrf1-LUC-#2 REV	Tsingke	CGAACAAGTGAAGCTTCCCTGGCCTTGAC
Nrf1-LUC-#3 FW	Tsingke	CACCCAACGCGCTAGCCCACTAACATCG
Nrf1-LUC-#3 REV	Tsingke	CTTCAGAGAAAAGCTTGCTGAAGGACCAGAATGTTTATGCT
Nrf1-LUC-#4 FW	Tsingke	CACCCAACGCGCTAGCCCACTAACATCG
Nrf1-LUC-#4 REV	Tsingke	ACTGCACTCAAGCTTGGGCAACAAGAGCAA
Nrf1-LUC-#5 FW	Tsingke	CACCCAACGCGCTAGCCCACTAACATCG
Nrf1-LUC-#5 REV	Tsingke	CTACTAAGCTTGACTATTCCGTCCA
Nrf1-LUC-#6 FW	Tsingke	CACCCAACGCGCTAGCCCACTAACATCG
Nrf1-LUC-#6 REV	Tsingke	GTTCAAGCTTCCGGACAAAGTC
Nrf1-LUC-#7 FW	Tsingke	CACCCAACGCGCTAGCCCACTAACATCG
Nrf1-LUC-#7 REV	Tsingke	CTGGTAAGCTTCTGCCCGGATAC
Nrf2 FW	Tsingke	GAGCCCGGTACCACGGTCCACAGCTC
Nrf2 REV	Tsingke	AAAACTAGCTCGAGAAAGGTCAAATCCTCCT
Nrf2 sgRNA-1 FW	Tsingke	AAACACCGTATTTGACTTCAGTCAGCGA
Nrf2 sgRNA-1 REV	Tsingke	CTCTAAAACTCGCTGACTGAAGTCAAATA
Nrf2 sgRNA-2 FW	Tsingke	AAACACCGTGCATACCGTCTAAATCAAC
Nrf2 sgRNA-2 REV	Tsingke	CTCTAAAACGTTGATTTAGACGGTATGCA
Nrf2 sgRNA-3 FW	Tsingke	AAACACCGTGGATTTGATTGACATACTT
Nrf2 sgRNA-3 REV	Tsingke	CTCTAAAACAAGTATGTCAATCAAATCCA
Nrf2-LUC FW	Tsingke	CCAGGAGTTTGGTACCAGCCTGGGCAACATAGTGA
Nrf2-LUC REV	Tsingke	CCAGCTCCAAGTAGATCTTGATGAGCTGTGGA
PTEN-LUC FW	Tsingke	GGTACTTGGAGGCTGGTACCATATTCTAGCAC
PTEN-LUC REV	Tsingke	CGGGAGATCTGAGGGCAGGGCAGGGCA
PTEN-LUC-M1 FW	Tsingke	GAGCATTGTTTTCACCTGGTCCTTTTCACCTGTGCACAGGTAACCTCAG
PTEN-LUC-M1 REV	Tsingke	GTGCGTTGAGCAGTGTCACTGACTCGAGTCTGAGGTTACCTGTG
PTEN-LUC-M2 FW	Tsingke	GAGCAGCGTGGTCACCTGGTCCTTTTCACCTGTGCACAGGTAACCTCAG
PTEN-LUC-M2 REV	Tsingke	GTGCGTTGATTAGTTTCACTGACTCGAGTCTGAGGTTACCTGTG
PTEN-UTR FW	Tsingke	ATGGCAATAGGACCTCGAGTCAGATTACCAGTTATAGGAACAATTCTC
PTEN-UTR REV	Tsingke	CTTTCCATATGCTGGCGGCCGCGTACAGAATAATAGACAAAAGC
PTEN-UTR-M FW	Tsingke	CATATTGGTGCTAGAAAAAAGGAAGTGAATCTGTATTGGGGTACAG
PTEN-UTR-M REV	Tsingke	TGTACCCCAATACAGATTCACTTCCTTTTTTCTAGCACCAATATGCT
Recombinant DNA		
pARE-luc	Zhang’s [97]	N/A
pcDNA3.1	invitrogen	V79020
pGL3-Basic	Promega	VQP0121
pGL3-promoter	Promega	VQP0124
pRL-TK	Promega	VQP0126
psiCHECK2	Promega	C8021
Software and Algorithms		
Canvas X	Canvas GFX, Inc.	https://www.canvasgfx.com/
Chromas 2.4.1	Technelysium Pty Ltd.	http://technelysium.com.au/wp/chromas/
cytoscape	[98]	http://www.cytoscape.org/
Excel	Microsoft	https://www.microsoft.com/
FlowJo 7.6.5.	FlowJo	https://www.flowjo.com/
KEGG	Kanehisa Laboratories	https://www.kegg.jp/
Primer Premier 5	PREMIER Biosoft International	https://www.PremierBiosoft.com/
Targetscan 7.2	[99]	http://www.targetscan.org/vert_72/
Venny 2.1.0	BioinfoGP, CNB-CSIC	http://bioinfogp.cnb.csic.es/tools/venny/index.html
Others		
Cas9/gRNA Construct Kit	v-solid	VK001
KeyGEN DAPI staining kit	KeyGEN BioTECH	KGA215
DAB kit	Boster Biological Technology	AR1022
Dual-luciferase reporter assay system	Promega	E1910
FastTALETM TALEN Assembly Kit	SIDANSAI	2801
GoTaq® qPCR Master Mix	Promega	A6001
Hematoxylin and Eosin Staining Kit	Beyotime	C0105
Lenti-Pac HIV Expression Packaging Kit	Gene Copoeia	HPK-LvTR
Lipofectamine® 3000 Transfection Kit	Invitrogen	L3000-015
Nuclei Isolation Kit	Sigma	NUC101-1KT
Reactive Oxygen Species Assay Kit	Beyotime	S0033
Revert Aid First Strand Synthesis Kit	Thermo	K1622
RNAsimple Total RNA Kit	Tiangen Biotech	DP419

## Supplementary Materials

The following are available online at http://www.mdpi.com/2072-6694/10/12/520/s1, Figure S1: The human Nrf1- and Nrf2-specific gene-editing constructs, Figure S2: Distinct cellular responses of the PCOX2-luc reporter gene to TPA, Figure S3: The JNK inhibitor blocks the Nrf1α^−/^^−^ -leading increase of COX2, Figure S4: Activation of some AP-1 components in Nrf1α^−/^^−^ cells, Figure S5: Cross-talks between Nrf1 and Nrf2 to regulate COX2, Figure S6: Distinctions in subcellular distributions of Nrf1 and Nrf2 in different cell lines, Figure S7: Genetic analysis of COX1 regulation, Figure S8: Differences in transcriptional expression of proteasomal subunits regulated by Nrf1 and Nrf2, Figure S9: Validation of cross-talks between Nrf1 and Nrf2 signaling consistently in distinct cell lines, Figure S10: Subtle nuances in distinct cell cycles and apoptosis processes, Figure S11: Opposite changes in DEGs measured from transcriptome in distinct cell lines, Figure S12: Opposite alterations in DEGs measured from transcriptome in Nrf2^−/^^−ΔTA^ and caNrf2^ΔN^ cells, Table S1: KEGG pathway enrichment analysis of DEGs in Nrf1α^−/−^ vs. WT cells, Table S2: KEGG pathway enrichment analysis of DEGs in Nrf1α^−/^^−^+siNrf2 vs. WT cells, Table S3: KEGG pathway enrichment analysis of DEGs in Nrf2^−/^^−ΔTA^ vs. WT cells.
