# Supplementary material for: Oncogenic Activation of Nrf2, Though as a Master Antioxidant Transcription Factor, Liberated by Specific Knockout of the Full-Length Nrf1α that Acts as a Dominant Tumor Repressor
Source: Cancers (Basel). 2018 Dec 17;10(12):520. doi: 10.3390/cancers10120520 (PMC6315801; doi:10.3390/cancers10120520)
Supplement: Supplementary file 1 [file cancers-10-00520-s001.pdf]

Supplementary Materials: Oncogenic Activation of Nrf2, Though as a Master Antioxidant Transcription Factor, Liberated by Specific Knockout of the Full-Length Nrf1α that Acts as a Dominant Tumor Repressor

Lu Qiu, Meng Wang, Shaofan Hu, Xufang Ru, Yonggang Ren, Zhengwen Zhang, Siwang Yu and Yiguo Zhang

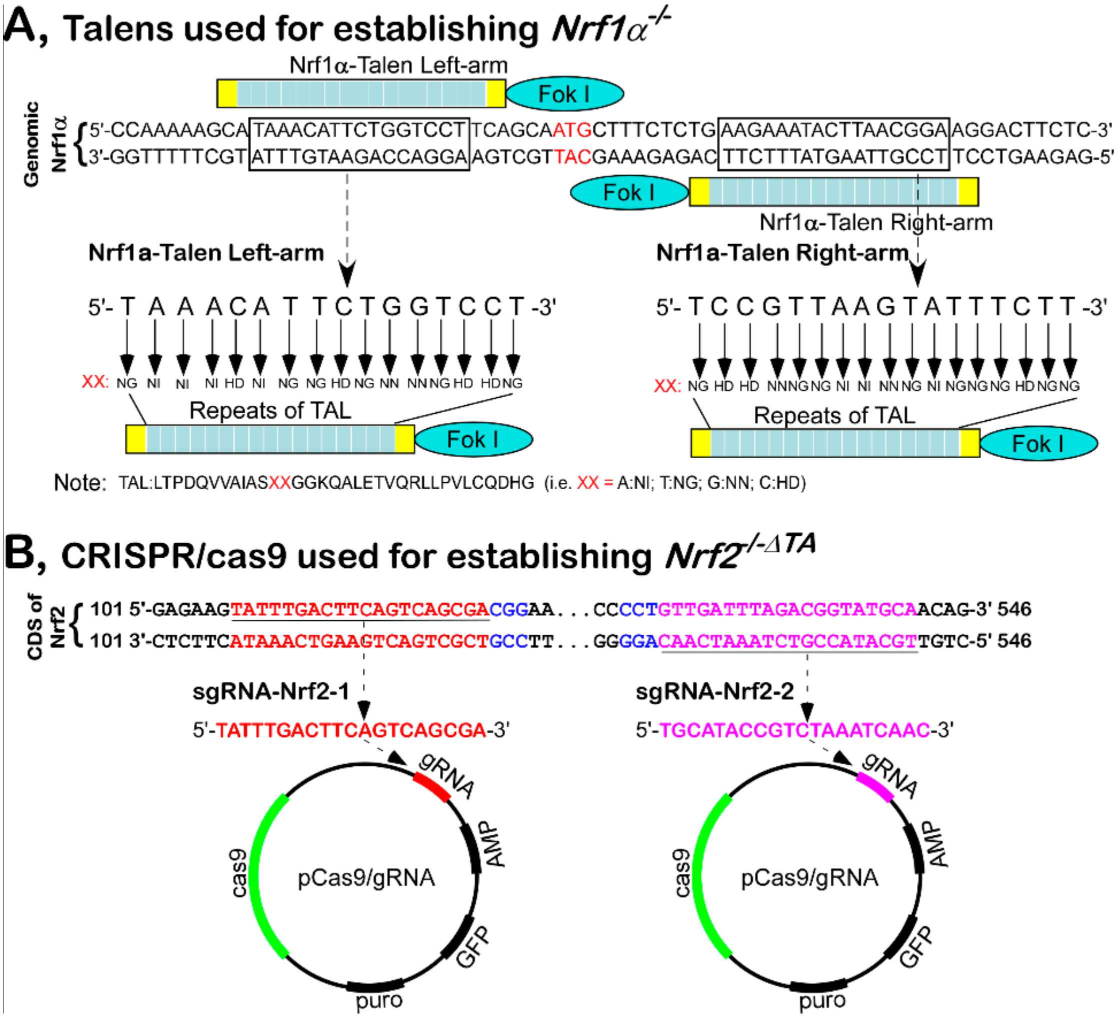

Figure S1. *Cont.*

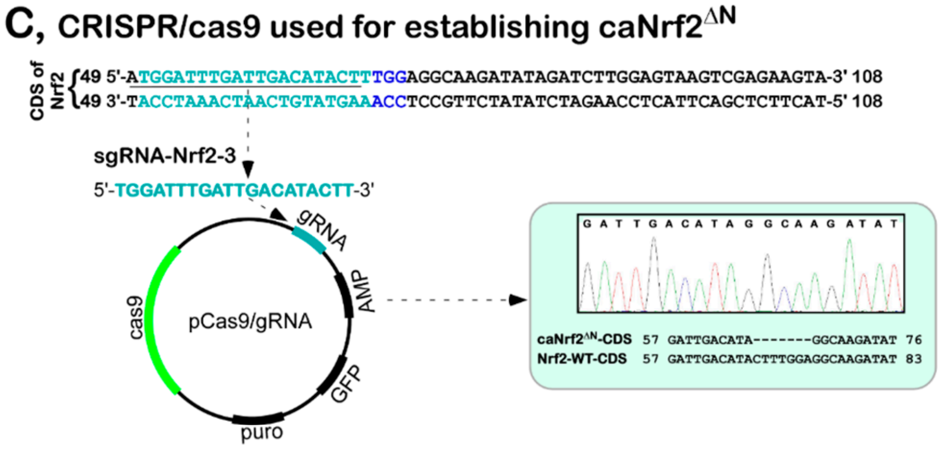

Figure S1. The human *Nrf1-* and *Nrf2-specific* gene-editing constructs. (**A**) Nrf1α-specific targeting constructs for TALEN-mediated gene editing. Both its left- and right- arms were designed for deletion of the first translation initiation codons of the *Nrf1* gene (i.e., *Nrf1α*$^{-/-}$). (**B**) Nrf2-specific constructs for CRISPR/CAS9-directed gene editing. They were designed for deleting a fragment of the *Nrf2* gene encoding most of both Neh4 and Neh5 domains (to yield an inactive *Nrf2*$^{-/-\Delta TA}$). (**C**) Another Nrf2-specific editing construct by CRISPR/CAS9. It was designed for the dominant-active mutant of Nrf2, so as to delete the sequence encoding the N-terminal Keap1-binding domain. The resulting mutant (i.e., *caNrf2*$^{\Delta N}$) was aligned with wild-type nucleotide sequence of *Nrf2*.

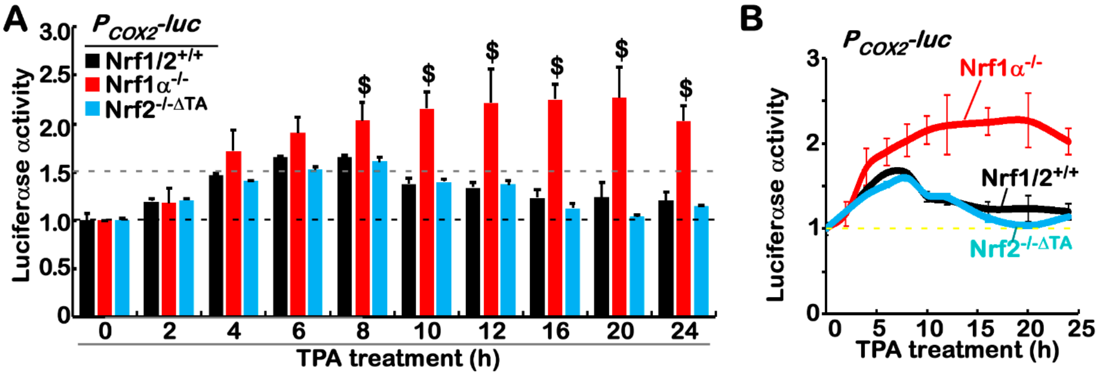

Figure S2. Distinct cellular responses of the *P*COX2*-luc* reporter gene to TPA. (**A**) *Nrf1/2*$^{+/+}$, *Nrf1α*$^{-/-}$ and *Nrf2*$^{-/-\Delta TA}$ cells were transfected with the *P*COX2*-luc* and *pRL-TK* reporters for 12 h, and then treated with100 nM of TPA for indicated lengths of time, before being measured for the luciferase activity. The data are shown as mean ± SEM ($n = 3 \times 3$; $, $p < 0.01$ compared with the untreated control values). (**B**) The above data are shown graphically.

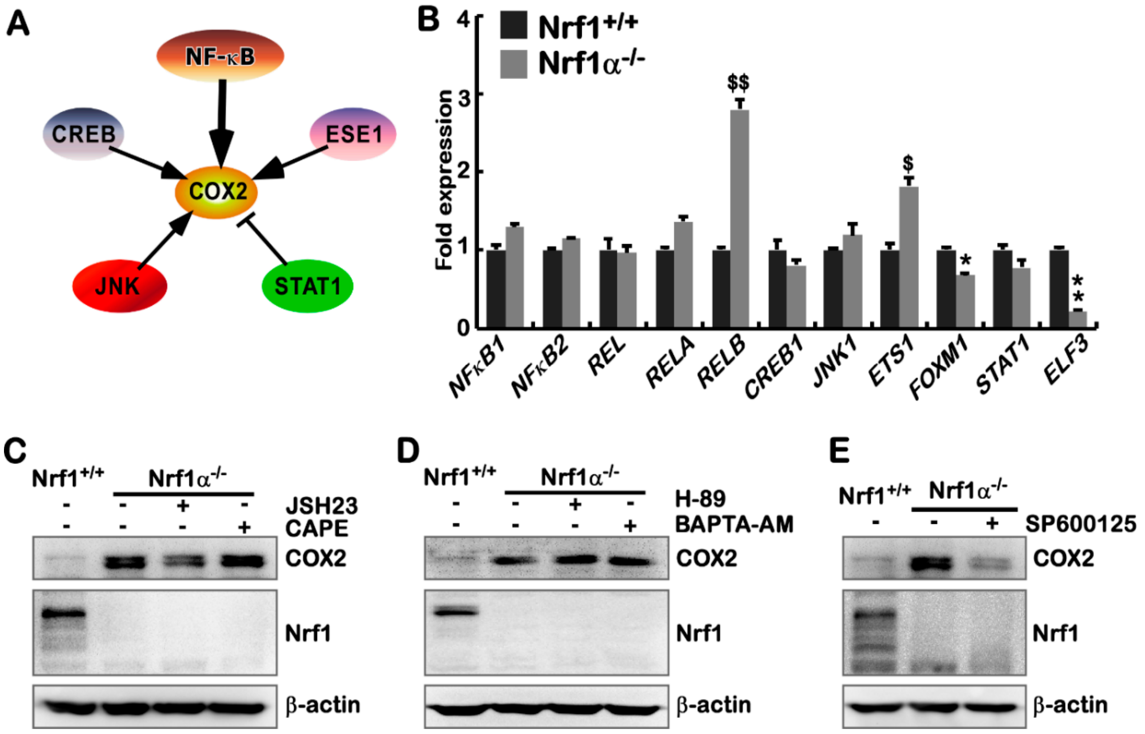

Figure S3. The JNK inhibitor blocks the *Nrf1α*$^{-/-}$ -leading increase of COX2. (**A**) Schematic representation of potential upstream signaling to regulate COX2. (**B**) Alterations in the indicated gene expression in *Nrf1α*$^{-/-}$, compared with *Nrf1/2*$^{+/+}$, cells. The data were obtained from transcriptome and are shown as mean ± SEM (*n* = 3; * *p* < 0.01; ** *p* < 0.001; $, *p* < 0.01; $$, *p* < 0.001). (**C**–**E**) *Nrf1α*$^{-/-}$ cells were treated for 24 h with (*C*) 20 μM of JSH23, 25 μM of CAP, (*D*) 10 μM of H-89, 1 μM of BAPTA-AM, or (**E**) 20 μM of SP600125, before COX2 was examined by western blotting.

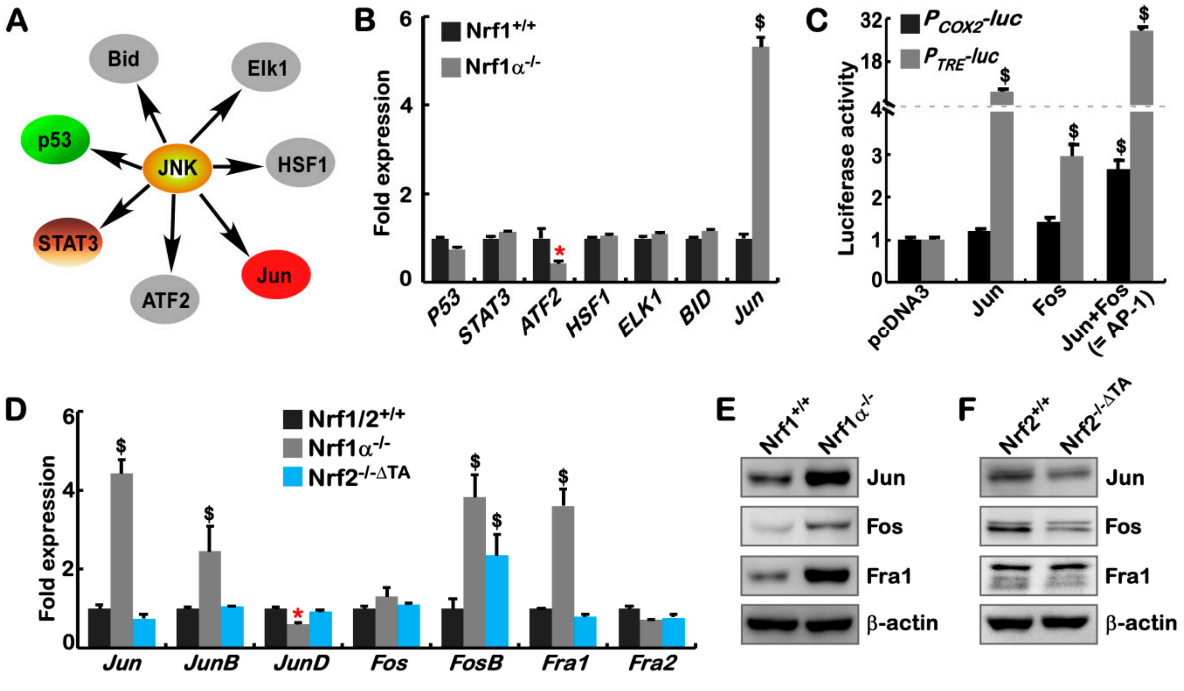

Figure S4. *Cont.*

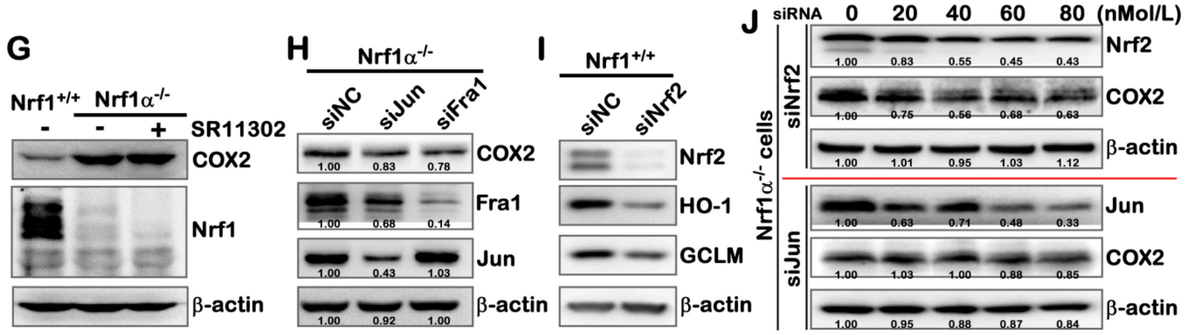

Figure S4. Activation of some AP-1 components in $Nrf1\alpha^{-/-}$ cells. (**A**) The cartoon shows possible JNK signaling to downstream targets. (**B**) The transcriptome analysis of major downstream genes regulated by JNK signaling. The data are shown as mean ± SEM ($n = 3$, * $p < 0.01$; $$, $p < 0.001$ compared with wild-type values). (**C**) Either *P*COX2*-luc* or PTRE-luc together with *pRL-TK* were co-transfected with each of indicated expression constructs or empty pcDNA3 vector and allowed for 24-h recovery, before being determined. The data are shown as mean ± SEM ($n = 3 \times 3$; $, $p < 0.01$; $$, $p < 0.001$). (**D**) The real-time qPCR analysis of distinct AP-1 subunits at their mRNA levels in $Nrf1/2^{+/+}$, $Nrf1\alpha^{-/-}$ and $Nrf2^{-/-\Delta TA}$ cells. The data are shown as mean ± SEM ($n = 3 \times 3$, * $p < 0.01$, $ $p < 0.01$; $$ $p < 0.001$). (**E**) Western blotting of JUN, FOS, and Fra1 abundances in $Nrf1\alpha^{-/-}$ and $Nrf1/2^{+/+}$, cells. (**F**) Abundances of JUN, FOS, and Fra1 was visualized western blotting of $Nrf2^{-/-\Delta TA}$ and $Nrf1/2^{+/+}$ cells. (**G**) $Nrf1\alpha^{-/-}$ cells were treated with 4 μM of SR11302 for 24 h before COX2 were examined by western blotting. (**H**) $Nrf1\alpha^{-/-}$ cells were allowed for knockdown by siJUN (60 nM) and siFOSL1 (60 nM) for 24 h, respectively, before COX2, Fra1 and JUN were determined by western blotting. (**I**) $Nrf1/2^{+/+}$ cells were subjected to silencing of siNrf2 (60 nM) and allowed for 24-h recovery, before Nrf2, HO1 and GCLM were visualized by immunoblotting. (**J**) $Nrf1\alpha^{-/-}$ cells were subjected to silencing of Nrf2 or Jun by their specific siRNAs at indicated doses (20, 40, 60, 80 nM) and then allowed for 24-h recovery from transfection, before Nrf2, Jun and COX2 were determined by western blotting.

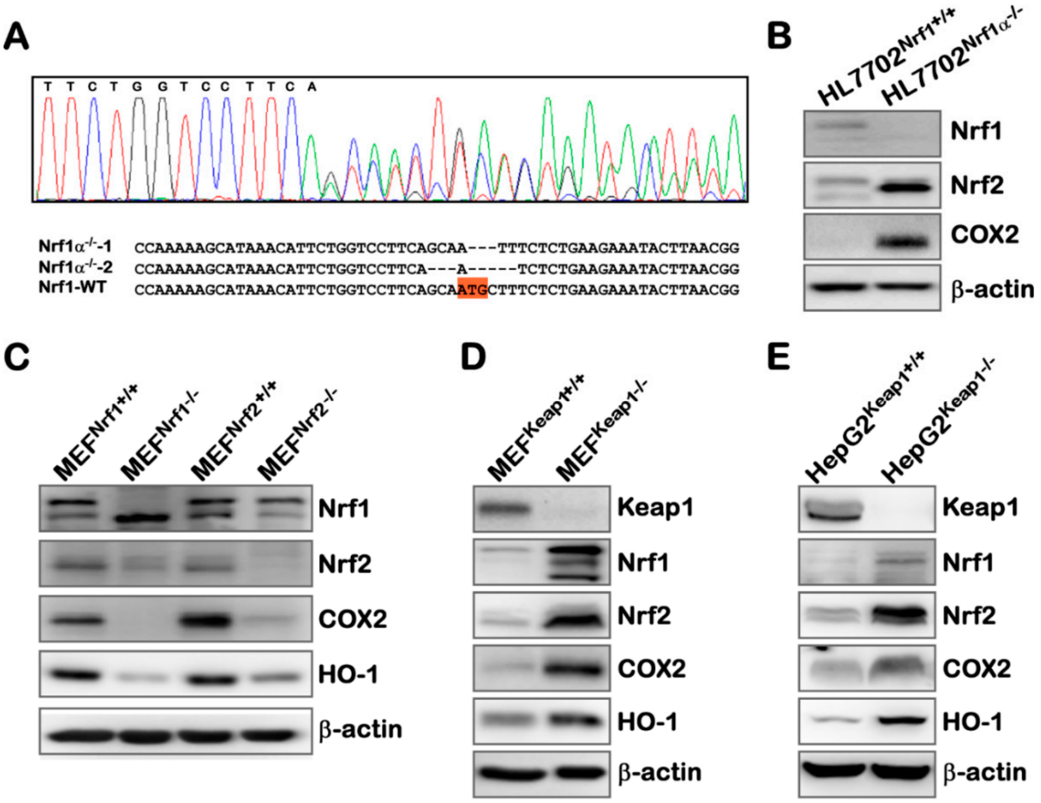

Figure S5. *Cont.*

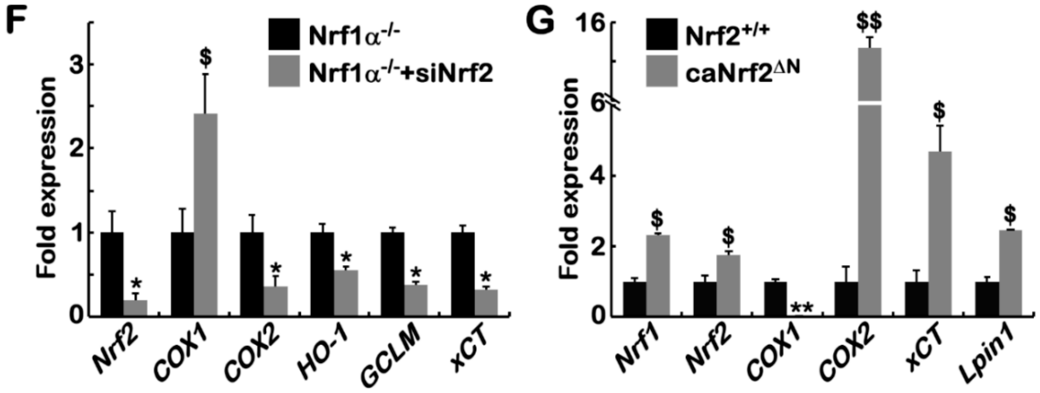

Figure S5. Cross-talks between Nrf1 and Nrf2 to regulate COX2. (**A**) Identification of HL7702$^{Nrf1\alpha-/-}$ by the genomic site-specific sequencing. The resulting mutant of *Nrf1α* was aligned with the wild-type nucleotide sequence. (**B**) Distinctions of Nrf1, Nrf2 and COX2 in between HL7702$^{Nrf1\alpha-/-}$ and HL7702$^{Nrf1+/+}$ cells was observed by western blotting. (**C**) Subtle nuances in the abundances of Nrf1, Nrf2, COX2 and HO-1 in between MEF $^{Nrf1+/+}$, MEF $^{Nrf1-/-}$, MEF $^{Nrf2+/+}$ and MEF $^{Nrf2-/-}$ were determined by Western blotting. (**D**) Alterations in the expression of Keap1, Nrf1, Nrf2, COX2 and HO-1 in between MEF $^{Keap1+/+}$ and MEF$^{Keap1-/-}$ were detected by western blotting. (**E**) Differences of Keap1, Nrf1, Nrf2, COX2, HO-1 abundances in between HepG2 $^{Keap1+/+}$ and HepG2$^{Keap1-/-}$ were visualized by western blotting. (**F**) Differential expression of *Nrf2, COX1, COX2, HO-1, GCLM* and *xCT* at mRNA levels in *Nrf1α*$^{-/-}$ and *Nrf1α*$^{-/-}$+siNrf2 cells were determined by the transcriptome. The data are shown as mean ± SEM ($n = 3$, * $p < 0.01$; $ $p < 0.01$). (**G**) Both *Nrf2*$^{-/-\Delta TA}$ and *caNrf2*$^{\Delta N}$ cell lines differentially expressed mRNA levels of *Nrf1, Nrf2, COX1, COX2, xCT* and *Lpin1.* The transcriptome FPKM data are shown as mean ± SEM ($n = 3$, ** $p < 0.001$; $ $p < 0.01$, $$ $p < 0.001$).

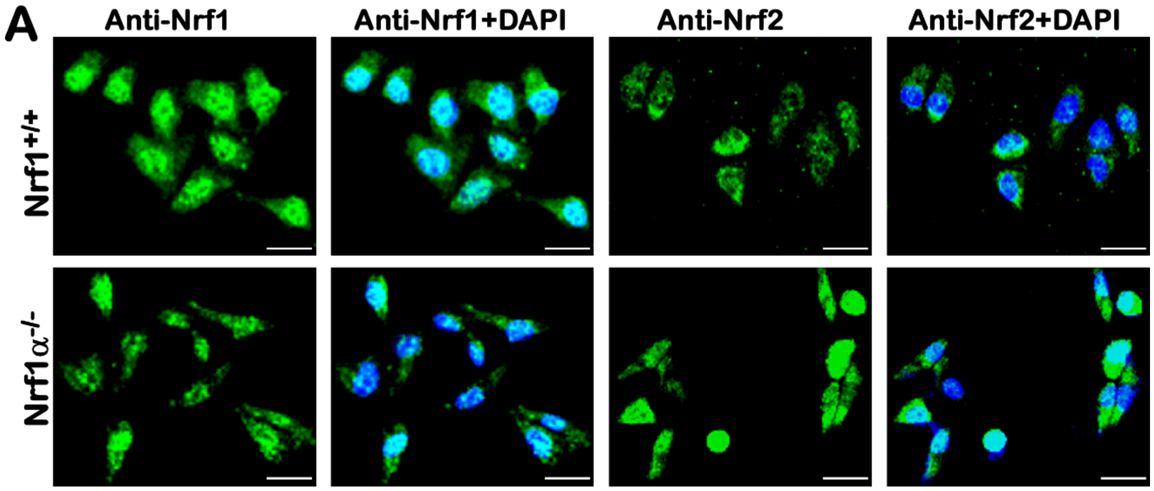

Figure S6. *Cont.*

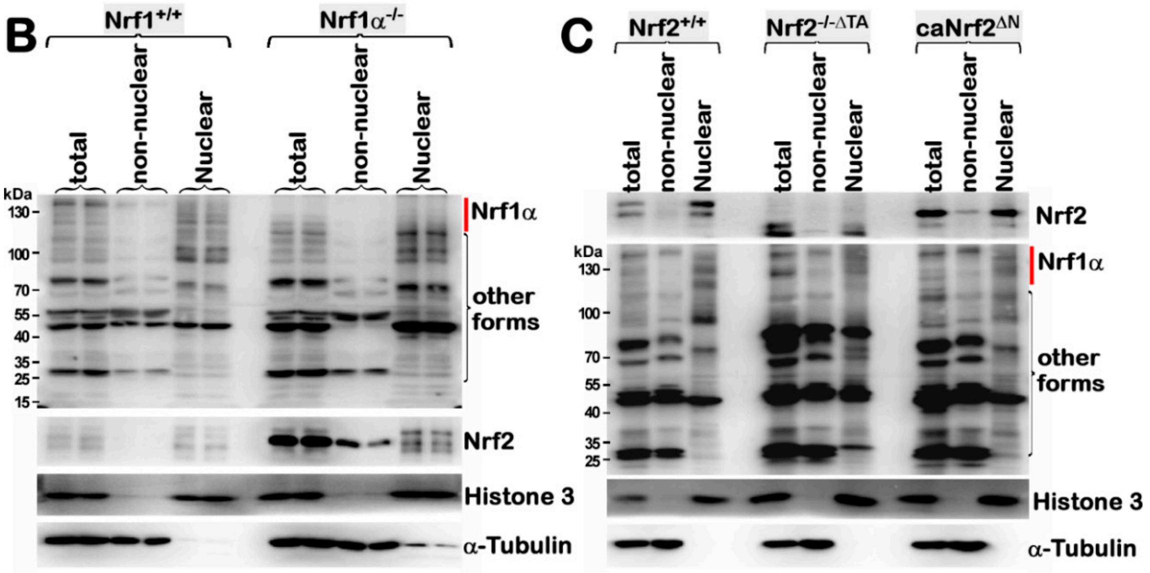

Figure S6. Distinctions in subcellular distributions of Nrf1 and Nrf2 in different cell lines. (**A**) Confocal images of Nrf1 and Nrf2 in distinct subcellular distributions were acquired by immuocytochemistry with their primary antibodies, along with FITC-labeled second antibody. The nuclear DNA was stained by DAPI. Scale bar: 5 μm. (**B**) Subcellular fractionation of *Nrf1α*$^{+/+}$ and *Nrf1α*$^{-/-}$ cell lines were subject to evaluation of the nuclear-cytoplasmic distribution of Nrf1 and Nrf2 by Western blotting. Additional antibodies against Histone H3 and β-tubulin were used as two distinct markers of nuclear and cytoplasmic proteins, respectively. (**C**) Subcellular distributions of Nrf1 and Nrf2 were also examined by fractionation of *Nrf2*$^{+/+}$, *Nrf2*$^{-/-ΔTA}$, and *caNrf2*^{ΔN} cell lines.

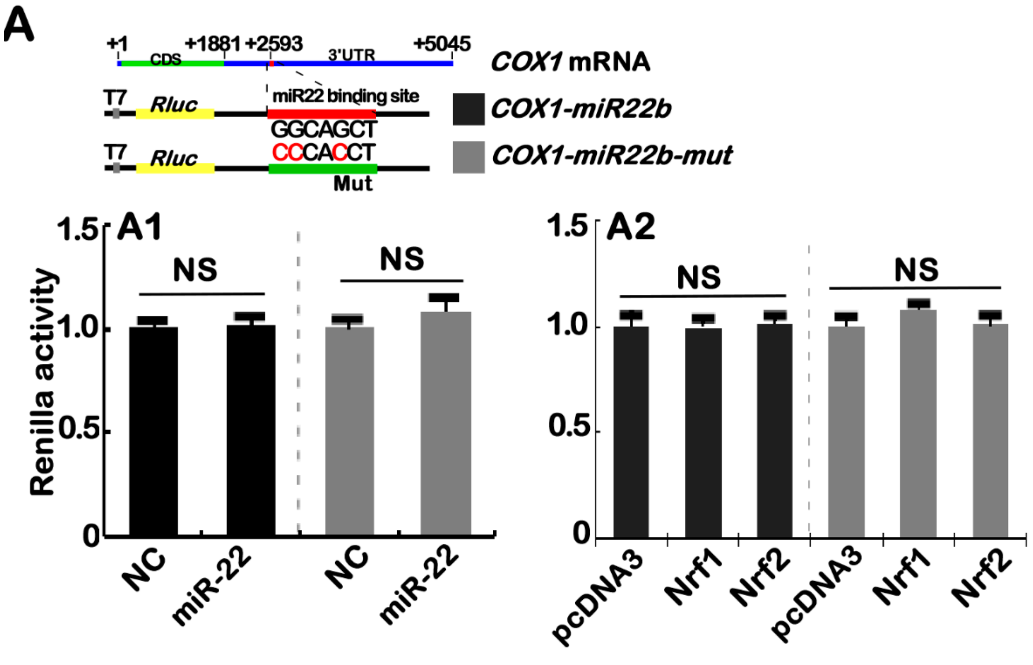

Figure S7. *Cont.*

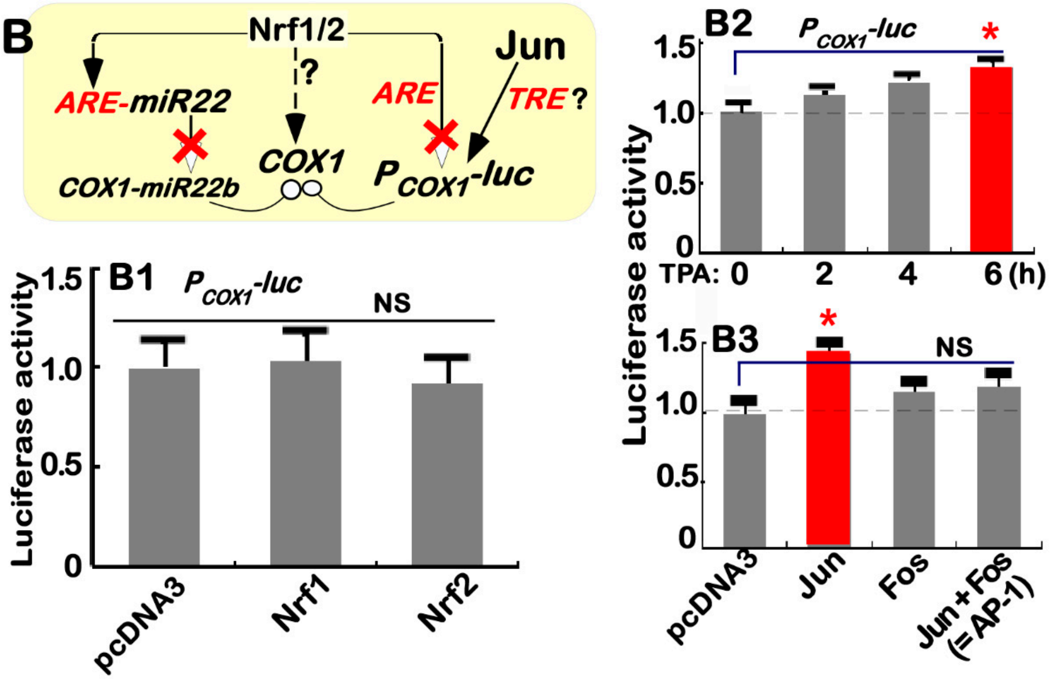

Figure S7. Genetic analysis of COX1 regulation. (**A**) The *COX1-miR22b* was constructed as above, which contains miR-22 binding site which in the COX1's 3'UTR region(upper). *Nrf1/2*$^{+/+}$ cells were co-transfected with *COX1-miR22b* or *COX1-miR22b-mut*, together with miR-22 or NC plasmids (A1), or pcDNA3, an expression construct for Nrf1 or Nrf2 (A2), and then allowed for 24-h recovery before being determined. The data are shown as mean ± SEM ($n = 3 \times 3$, NS = no statistical difference). (**B**) *Nrf1/2*$^{+/+}$ cells were co-transfected with the *P*$_{COX1}$*-luc* and *pRL-TK* (**B1** to **B3**), plus pcDNA3 or indicated expression constructs for Nrf1, Nrf2 (**B1**), Jun, Fos or Jun+pFos (**B3**), and allowed for 24-h recovery, before being treated (**B2**), or were not treated (**B1**,**B3**), with 100 nM of TPA for 2–6 h, prior to being measured for the luciferase activity. The data are shown as mean ± SEM ($n = 3 \times 3$, * $p < 0.01$, NS = no statistical difference).

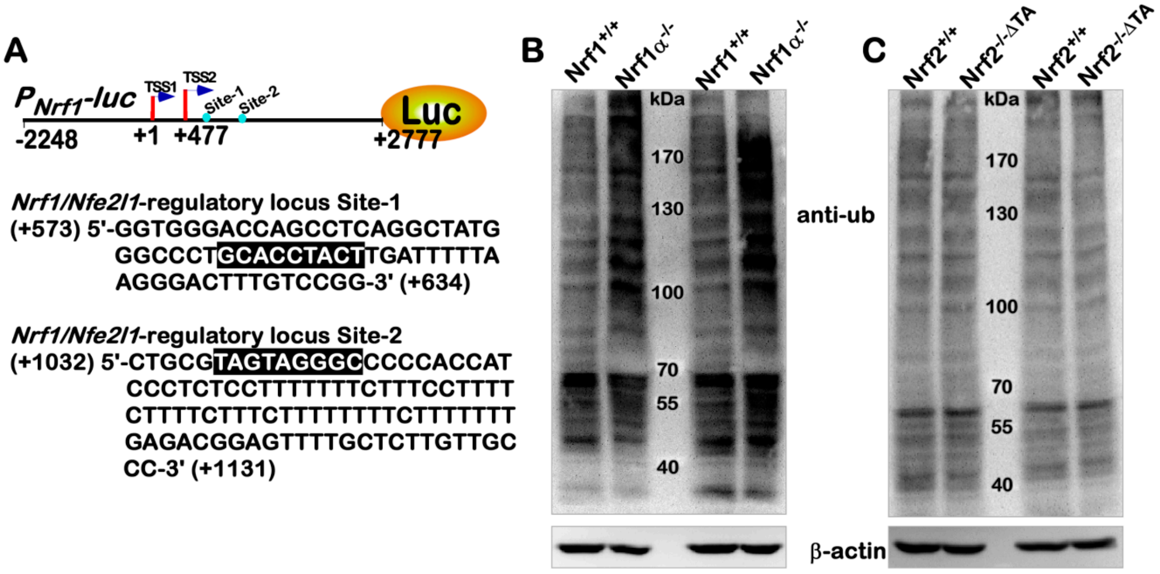

Figure S8. *Cont.*

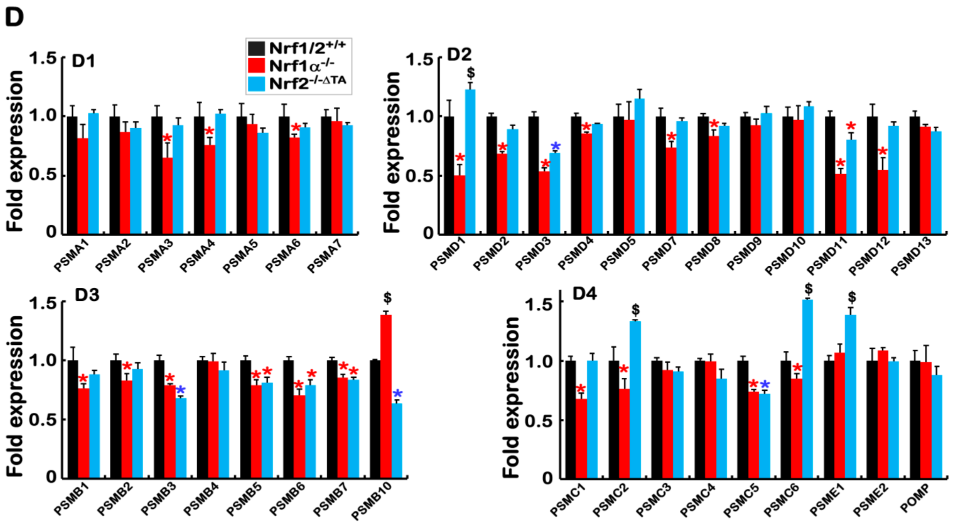

Figure S8. Differences in transcriptional expression of proteasomal subunits regulated by Nrf1 and Nrf2. (**A**) Two *cis-Nrf1/Nef2l1*-regulatory locus sites (i.e., Site-1 and Site-2) exist in this gene promoter, as located (*upper*). The nucleotide sequence of both Site-1 and Site-2 are shown. (**B**) Immunoblotting with antibodies against ubiquitinated proteins (i.e., anti-ub) in *Nrf1/2*$^{+/+}$ and *Nrf1α*$^{-/-}$ cells. (**C**) Almost no or less anti-ub cross-reactivity with ubiquitinated proteins in *Nrf1/2*$^{+/+}$ and *Nrf2*$^{-/-ΔTA}$ cells was observed. (**D**) Significant decreases in the expression of most of the 26S proteasomal subunits and related proteins were detected in *Nrf1α*$^{-/-}$ cells when compared with those in *Nrf1/2*$^{+/+}$. By contrast, almost no changes in the transcriptional expression of most proteasomal and related genes were compared in *Nrf2*$^{-/-ΔTA}$ with *Nrf1/2*$^{+/+}$ cells. The transcriptome data are shown as mean ± SEM (n = 3, * $p < 0.01$; $ $p < 0.01$).

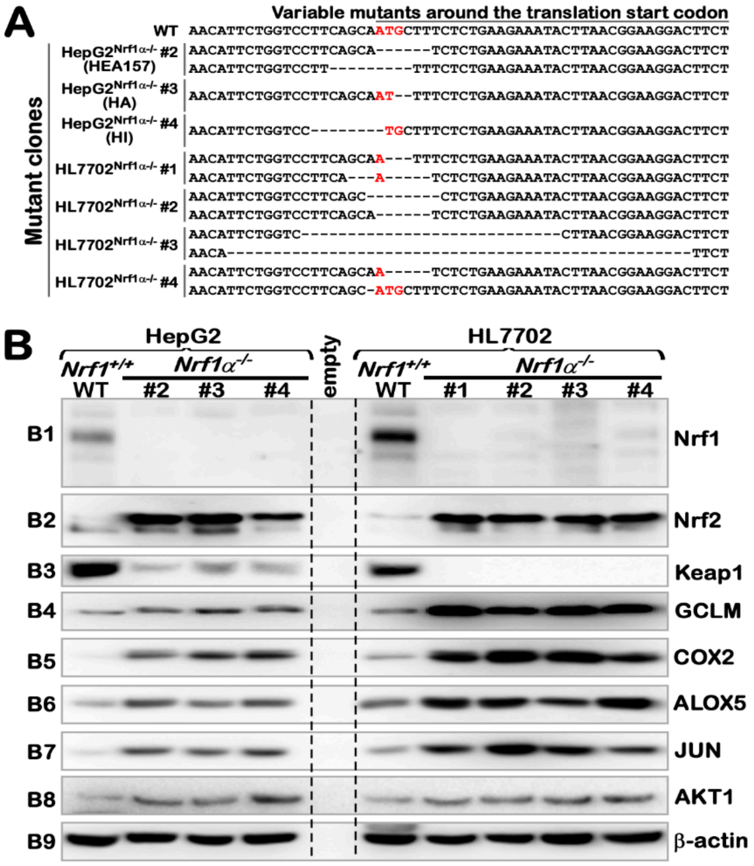

Figure S9. Validation of cross-talks between Nrf1 and Nrf2 signaling consistently in distinct cell lines. (**A**) The nucleotide alignment of the human wild-type (WT) *Nrf1* and its allelic mutants around the

translation start codons, all of which were confirmed to be true by DNA sequencing. (**B**) Consistent expression of Nrf1, Nrf2, Keap1, GCLM, COX2, ALOX5, JUN, and AKT1 was determined by Western blotting of distinct monoclonal cell lines of *Nrf1α*$^{-/-}$, which were derived from two progenitor HepG2 and HL7702 cell lines as indicated.

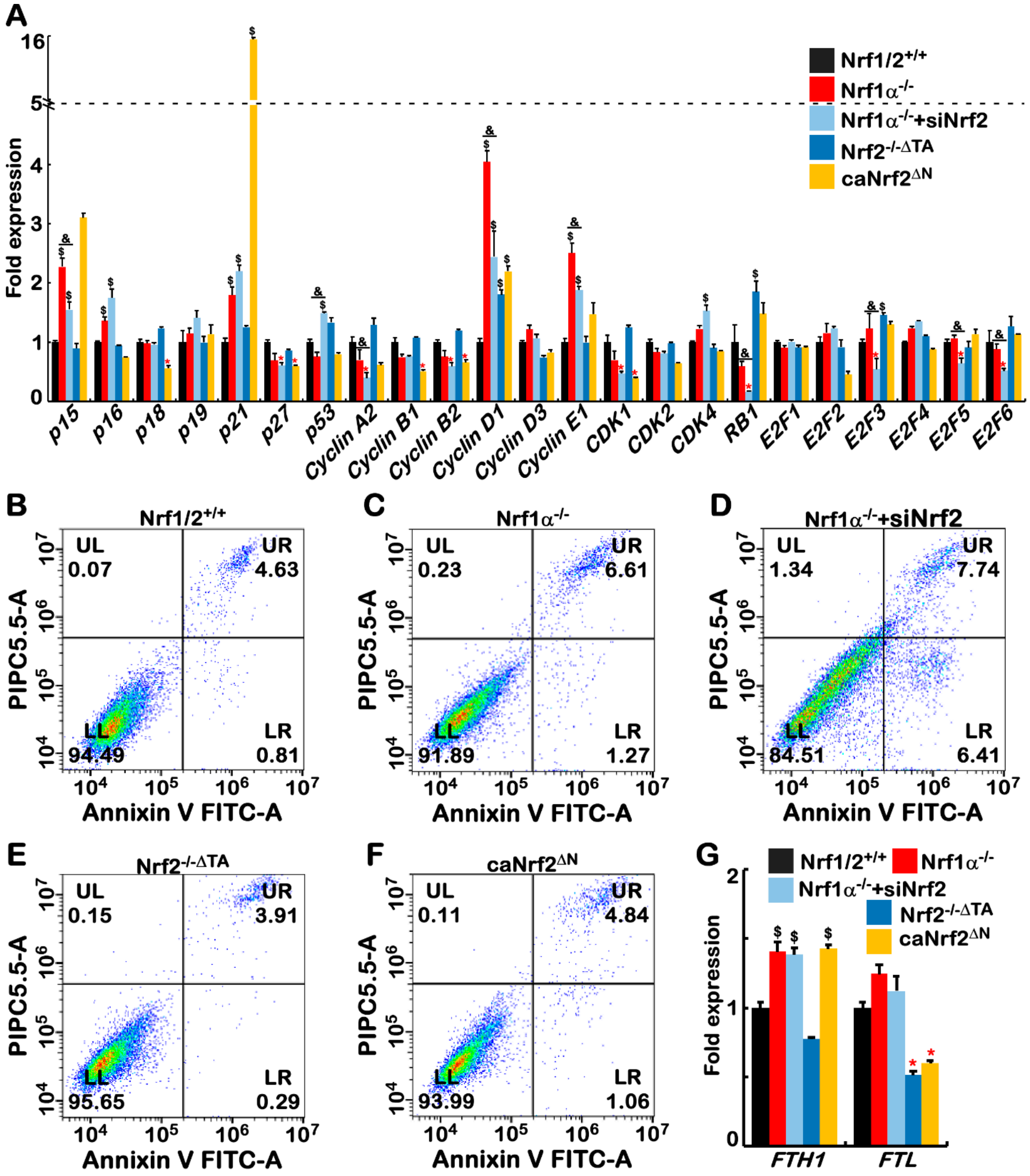

Figure S10. Subtle nuances in distinct cell cycles and apoptosis processes. (**A**) Changes in expression of cell cycle-related genes in five distinct cell lines as indicated. The transcriptome data are shown as mean ± SEM ($n = 3$, * $p < 0.01$; $ $p < 0.01$). (**B** to **F**) Flow cytometry analysis of apoptosis in five distinct cell lines as indicated. Abbreviations: UL, necrotic cells; UR, early apoptotic cells; LL, normal cells; LR, late apoptotic cells. (**G**) The expression of *FTH1* and *FTL* genes were detected by transcriptome sequencing. The data are shown as mean ± SEM ($n = 3$, * $p < 0.01$; $ $p < 0.01$).

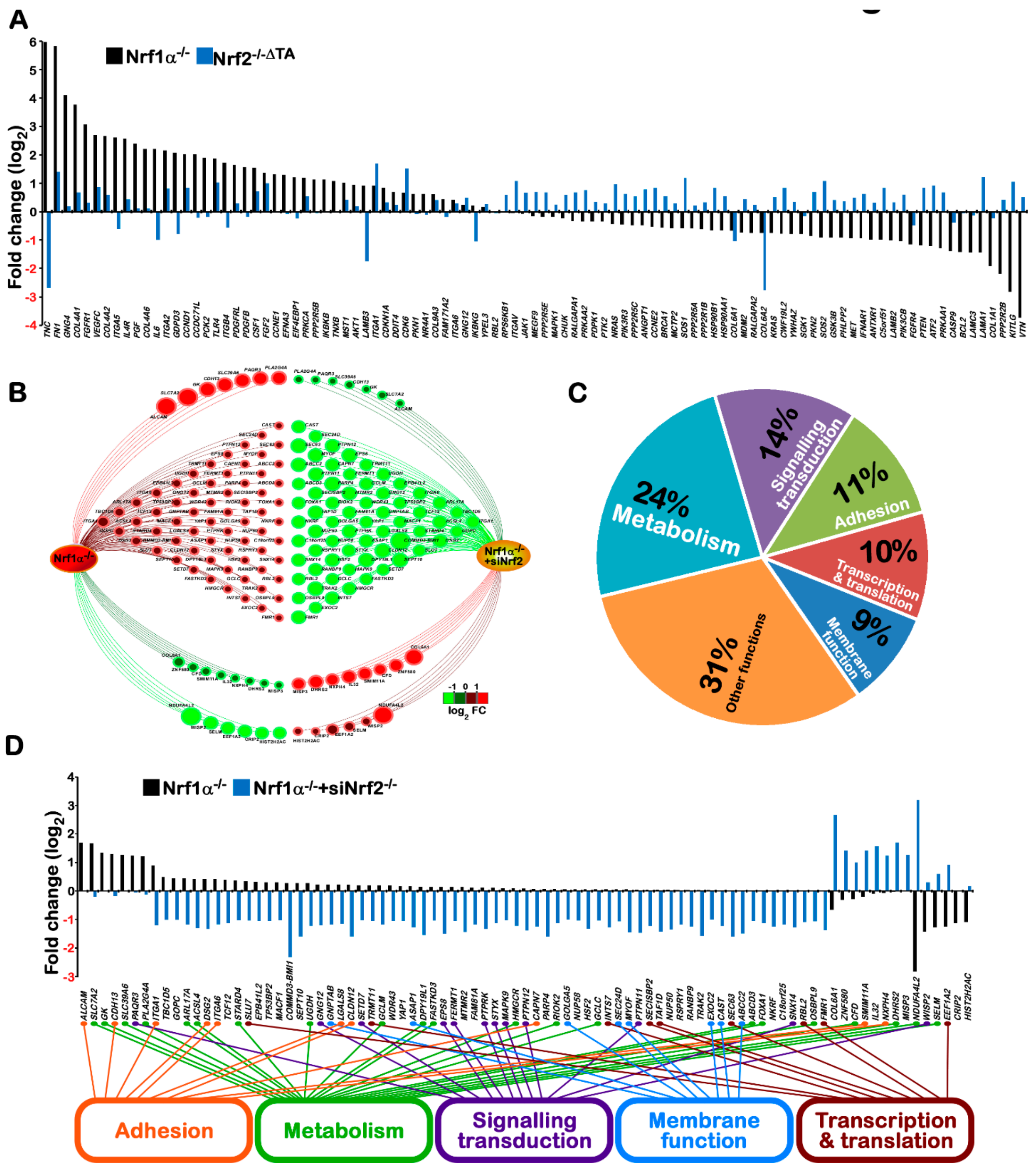

Figure S11. Opposite changes in DEGs measured from transcriptome in distinct cell lines. (**A**) Significant differences in the indicated DEGs responsible for PTEN-directed PI3K-AKT signaling pathways (also shown in Figure 7**B**,**C**) in between *Nrf1α*$^{-/-}$ and *Nrf2*$^{-/-\Delta TA}$ cell lines are shown graphically, after normalization to relevant values measured from *Nrf1/2*$^{+/+}$ cells by transcriptome sequencing ($n = 3$). (**B** to **D**) Opposite alterations in DEGs in between *Nrf1α*$^{-/-}$ and *Nrf1α*$^{-/-}$+siNrf2 cell lines after being normalized to those in *Nrf1/2*$^{+/+}$ cells are shown in different ways. The major functions of these genes are also classified.

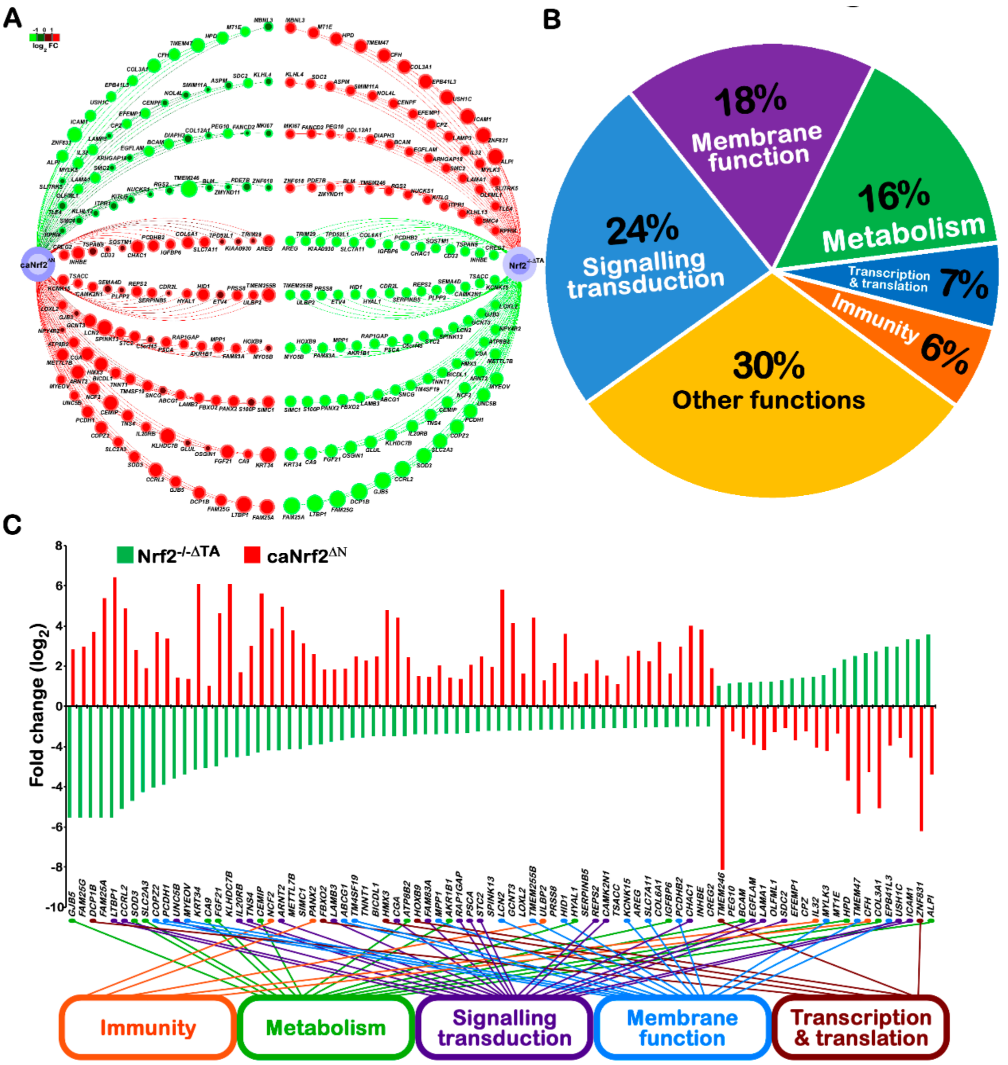

Figure S12. Opposite alterations in DEGs measured from transcriptome in *Nrf2*$^{-/-}$ and *caNrf2*$^{\Delta N}$ cells. These genes display opposite trends in their expression levels in between *Nrf2*$^{-/-\Delta TA}$ and *caNrf2*$^{\Delta N}$, after normalization to relevant values measured from *Nrf1/2*$^{+/+}$ cells by transcriptome sequencing (n = 3). The major functions of these genes are also classified.

Table S1. KEGG pathway enrichment analysis of DEGs in *Nrf1α*$^{-/-}$ vs. WT cells.

No.	Pathway	DEGs Genes with pathway annotation (1080)	All Genes with Pathway Annotation (19718)	*p*-Value	Pathway ID	Level 1
1	AGE-RAGE signaling pathway in diabetic complications	26 (2.41%)	145 (0.74%)	8.01E-08	ko04933	Human Diseases
2	Malaria	16 (1.48%)	63 (0.32%)	1.88E-07	ko05144	Human Diseases
3	Rheumatoid arthritis	19 (1.76%)	106 (0.54%)	4.39E-06	ko05323	Human Diseases
4	p53 signaling pathway	19 (1.76%)	114 (0.58%)	1.31E-05	ko04115	Cellular Processes
5	Small cell lung cancer	18 (1.67%)	115 (0.58%)	5.22E-05	ko05222	Human Diseases
6	Cytokine-cytokine receptor interaction	32 (2.96%)	279 (1.41%)	6.42E-05	ko04060	Environmental Information Processing
7	African trypanosomiasis	10 (0.93%)	43 (0.22%)	8.53E-05	ko05143	Human Diseases
8	NOD-like receptor signaling pathway	25 (2.31%)	211 (1.07%)	0.000236	ko04621	Organismal Systems
9	ECM-receptor interaction	21 (1.94%)	166 (0.84%)	0.000299	ko04512	Environmental Information Processing

10	Leishmaniasis	15 (1.39%)	100 (0.51%)	0.000346	ko05140	Human Diseases
11	TNF signaling pathway	19 (1.76%)	145 (0.74%)	0.000364	ko04668	Environmental Information Processing
12	Amoebiasis	20 (1.85%)	159 (0.81%)	0.000444	ko05146	Human Diseases
13	PI3K-Akt signaling pathway	45 (4.17%)	492 (2.5%)	0.00054	ko04151	Environmental Information Processing
14	Type I diabetes mellitus	11 (1.02%)	66 (0.33%)	0.000848	ko04940	Human Diseases
15	Focal adhesion	35 (3.24%)	363 (1.84%)	0.000861	ko04510	Cellular Processes
16	Legionellosis	13 (1.2%)	87 (0.44%)	0.000872	ko05134	Human Diseases

Table S2. KEGG pathway enrichment analysis of DEGs in *Nrf1α*$^{-/-}$+*siNrf2* vs. WT cells

**No.**	**Pathway**	**DEGs Genes with Pathway Annotation (2795)**	**All Genes with Pathway Annotation (19718)**	***p*-Value**	**Pathway ID**	**Level 1**
1	Cell cycle	45 (1.61%)	162 (0.82%)	4.48E-06	ko04110	Cellular Processes
2	FoxO signaling pathway	45 (1.61%)	181 (0.92%)	9.04E-05	ko04068	Environmental Information Processing
3	AGE-RAGE signaling pathway in diabetic complications	38 (1.36%)	145 (0.74%)	9.52E-05	ko04933	Human Diseases
4	Protein processing in endoplasmic reticulum	51 (1.82%)	220 (1.12%)	0.000215	ko04141	Genetic Information Processing
5	NOD-like receptor signaling pathway	49 (1.75%)	211 (1.07%)	0.00027	ko04621	Organismal Systems
6	Apoptosis - fly	24 (0.86%)	83 (0.42%)	0.000375	ko04214	Cellular Processes
7	Epithelial cell signaling in Helicobacter pylori infection	24 (0.86%)	83 (0.42%)	0.000375	ko05120	Human Diseases
8	Small cell lung cancer	30 (1.07%)	115 (0.58%)	0.000537	ko05222	Human Diseases
9	Epstein-Barr virus infection	58 (2.08%)	272 (1.38%)	0.000816	ko05169	Human Diseases
10	TNF signaling pathway	35 (1.25%)	145 (0.74%)	0.00092	ko04668	Environmental Information Processing

Table S3. KEGG pathway enrichment analysis of DEGs in *Nrf2*$^{-/-\Delta TA}$ vs. WT cells.

**No.**	**Pathway**	**DEGs Genes with Pathway Annotation (498)**	**All Genes with Pathway Annotation (19718)**	***p*-value**	**Pathway ID**	**Level 1**
1	Regulation of actin cytoskeleton	17 (3.41%)	331 (1.68%)	0.004692	ko04810	Cellular Processes
2	Axon guidance	14 (2.81%)	261 (1.32%)	0.006802	ko04360	Organismal Systems

© 2018 by the authors. Licensee MDPI, Basel, Switzerland. This article is an open access article distributed under the terms and conditions of the Creative Commons Attribution (CC BY) license (http://creativecommons.org/licenses/by/4.0/).
